# Targeting yeast topoisomerase II by imidazo and triazoloacridinone derivatives resulting in their antifungal activity

**DOI:** 10.1038/s41598-024-54252-0

**Published:** 2024-02-13

**Authors:** Kamila Rząd, Iwona Gabriel, Ewa Paluszkiewicz, Aleksandra Kuplińska, Mateusz Olszewski, Agnieszka Chylewska, Aleksandra M. Dąbrowska, Katarzyna Kozłowska-Tylingo

**Affiliations:** 1 Department of Pharmaceutical Technology and Biochemistry, Faculty of Chemistry and BioTechMed Center, Gdansk University of Technology, 11/12 Narutowicza Str., 80-233 Gdansk, Poland; 2 Department of Bioinorganic Chemistry, Faculty of Chemistry, University of Gdansk, Wita Stwosza 63, 80-308 Gdansk, Poland

**Keywords:** Antimicrobials, Biofilms, Fungi, DNA, Enzymes

## Abstract

Fungal pathogens are considered as serious factors for deadly diseases and are a case of medical concern. Invasive fungal infections also complicate the clinical course of COVID-19, leading to a significant increase in mortality. Furthermore, fungal strains' multidrug resistance has increased the demand for antifungals with a different mechanism of action. The present study aimed to identify antifungal compounds targeting yeast topoisomerase II (yTOPOII) derived from well-known human topoisomerase II (hTOPOII) poisons **C-1305** and **C-1311**. Two sets of derivatives: triazoloacridinones (**IKE1-8**) and imidazoacridinones (**IKE9-14**) were synthetized and evaluated with a specific emphasis on the molecular mechanism of action. Our results indicated that their effectiveness as enzyme inhibitors was not solely due to intercalation ability but also as a result of influence on catalytic activity by the formation of covalent complexes between plasmid DNA and yTOPOII. Lysine conjunction increased the strength of the compound's interaction with DNA and improved penetration into the fungal cells. Triazoloacridinone derivatives in contrast to starting compound **C-1305** exhibited moderate antifungal activity and at least twice lower cytotoxicity. Importantly, compounds (**IKE5-8**) were not substrates for multidrug ABC transporters whereas a derivative conjugated with lysine (**IKE7**), showed the ability to overcome *C. glabrata* fluconazole-resistance (MIC 32–64 µg mL^−1^).

## Introduction

In the last few years increasing importance is attached to problems caused by fungal pathogens. The diagnosis of fungal infections is not easy, and the therapy is complicated^1^. The fungistatic effect of fluconazole and its frequent use led to the increased resistance of *Candida* spp. strains^2,3^. Among 5 million fungal species, around 300 cause human diseases^4^. Fungemia is mainly caused by *Candida* spp. (60–80% cases)^5^, of which *C. albicans* constitutes 35% to 60% of isolates^6,7^. The *C. albicans* strain is a natural microflora in humans, but under certain conditions, it can pose a serious threat to health due to the possibility of causing opportunistic infections. There are no available vaccines against *C. albicans* infection and the preventability of invasive candidiasis is low. The *C. albicans* strain was included in the Critical Priority Group on the first WHO fungal priority pathogens list issued to highlight the problem of growing fungal resistance and to strengthen the global response to fungal infections^8^. Several antifungal compounds are highly effective in some cases, however, they have limitations in use: long half-life, widespread in tissues, and high toxicity^9^. The antifungal medications currently available for the treatment of invasive fungal infections belong to only four drug classes (azoles, echinocandins, pyrimidines, and polyenes), whereas two of which target ergosterol, a component of the fungal cell membrane^10^. Right now, there are known cases of fungi resistant to all approved oral drugs^11^. Therefore, a novel antifungal drug with different mechanisms of action is urgently needed.

Research indicates that fungal topoisomerases are crucial for the survival of some fungal strains, mammalian and fungal enzymes show diversity in terms of molecular structures and properties as well as differences in sensitivity to some human topoisomerase II (hTOPOII) inhibitors^12^. For example, previous research showed that *Candida* TOPOII demonstrates less sensitivity for m-AMSA (Amsacrine; 4′-(9-Acridinylamino)methanesulfon-m-anisidide, acridine-compound) and etoposide than human enzyme while podophyllotoxins are inactive against mammalian enzyme but inhibit the activity of fungal TOPOII^13^. Also, our results obtained for antitumor imidazoacridinone **C-1311** and triazoloacridinone **C-1305**, whose molecular mechanism of action is related to hTOPOII poisoning^14,15^, indicated that the fungal enzyme is much less sensitive to both compounds than the human one^16^. Moreover, our preliminary studies showed that acridine/acridone derivatives may effectively inhibit yeast TOPOII (yTOPOII), which could be related to their antifungal activity^16–18^. However, due to the existence of fungal cell wall and differences in membrane permeability, some yTOPOII inhibitors like 5-[(2-aminoethyl)amino]-8-hydroxy-6*H*-imidazo[4,5,1-de]acridin-6-one (previous described as Compound 1^16^), **C-1305**, **C-1311** cannot pass through the fungal cell wall and access their molecular target inside the cell. With this in mind, we have decided to modify the structures of the previously studied compounds^16^ including **C-1305**, **C-1311**, as well as **C-1330**, and more thoroughly analyzed the properties of a new group of triazoloacridinones (**IKE1-8**) **C-1305** derivatives and imidazoacridinones (**IKE9-14**) **C-1311** derivatives in terms of their influence on yTOPOII activity as well as their ability to affect fungal growth. Modifying the structure of a potential drug compound by conjugation with lysine is also proposed as an efficient approach to increase the ability to penetrate the fungal cell and decrease cytotoxicity^19,20^.

## Results

Our preliminary studies indicated that targeting yTOPOII by acridine/acridone derivatives might not always result in an antifungal effect. Unfortunately, due to the presence of a fungal cell wall and variances in membrane permeability, certain potent yTOPOII inhibitors face challenges in reaching their intended molecular target^16–18^. Unfavorable properties can be eliminated by modifying the lead structure for example by changing the lipophilicity or combining it with amino acids, which increases their bioavailability and decreases cytotoxicity^17,19–21^. An example of such an approach is the modification of nitroacridine derivative by lysine moiety that led to increased activity of the compound against fluconazole-resistant fungal cells^17^. Thus, the compounds analyzed by us exhibit variations in lipophilicity (having OCH_3_ or CH_3_ R groups instead of OH, as seen in **C-1305** and **C-1311** compounds, that cannot penetrate the fungal cell) and feature different substituents on the nitrogen atom (R^1^, R^2^: ethyl groups rather than methyl, similar to compound **C-1330** which effectively enters the fungal cell^22^). Additionally, some of them, incorporating lysine conjugation, are proposed as an efficient strategy to enhance fungal cell penetration while minimizing cytotoxicity. The structures of derivatives that were synthesized in this study are divided into two groups: **C-1305** analogs (triazoloacridinones group I) and **C-1311** derivatives (imidazoacridinones group II) (Table 1).

**Table 1:** The list of triazoloacridinone (group I) and imidazoacridinone (group II) analogs.


Compound	R	R^1^	R^2^
Group (I)			
** IKE1**	CH_3_	CH_3_	CH_3_
** IKE2**	CH_3_	CH_2_CH_3_	CH_2_CH_3_
** IKE3**	OCH_3_	CH_3_	CH_3_
** IKE4**	OCH_3_	CH_2_CH_3_	CH_2_CH_3_
** IKE5**	OCH_3_	H	H
** IKE6**	CH_3_	H	H
** IKE7**	OCH_3_	H	
** IKE8**	CH_3_	H	
** C-1305**	OH	CH_3_	CH_3_
Group (II)			
** IKE9**	OCH_3_	CH_3_	CH_3_
** IKE10**	CH_3_	H	H
** IKE11**	CH_3_	H	
** IKE12**^16^	OH	H	H
** IKE13**	OCH_3_	H	H
** IKE14**	OCH_3_	H	
** C-1330**	OCH_3_	CH_2_CH_3_	CH_2_CH_3_
** C-1311**	OH	CH_2_CH_3_	CH_2_CH_3_

### Synthesis

Triazolo- and imidazoacridinone derivatives with 1-diaminoalkyl substituent with two or three methylene groups between two amino centers, and with the distal amino group bearing Lys were synthesized. The first group, 5-[(aminoalkyl)amino]-8-substituted-6*H*-[1,2,3]triazolo[4,5,1-de]acridin-6-one presented in Figure S1 (collected in Supplementary Information, SI) (**IKE1-4**) and **C-1305** were prepared according to the previously reported procedure^23,24^. Acridone derivatives with a side chain with an unsubstituted amino terminal group (**IKE5-6**), were obtained by condensation of 1-chloroacridinone derivatives with an excess of the corresponding amine in DMA (*N*,*N*-Dimethylacetamide), and at the next stage these compounds were condensed with protected amino acid in the presence of coupling agents in DMSO/TEA (Triethylamine) and then deprotection of nitrogen atoms (**IKE7-8**).

The second group of synthesized compounds were imidazoacridinone derivatives (**IKE9-14**) presented in Figure S1 (collected in Supplementary Information, SI). Compounds **C-1311**, **C-1330**, **IKE9-10**, and **IKE12-13** were obtained from 1-chloro-7-substituted-4-nitro-9(10H)-acridinone, through the substitution with an excess of aliphatic amines, reduction and cyclization of the resulting derivatives. This method of synthesis was described previously^25,26^. Compounds **IKE10** and **IKE13** were used as a starting material for the synthesis of derivatives **IKE11** and **IKE14**. The procedure was the same as for the analogous triazoloacridinone derivatives. All derivatives, except for **C-1305**, **C-1311**, **C-1330**, and **IKE12** are the newly synthesized ones^14–16^.

### Inhibition of yeast topoisomerase II in vitro

Inhibition of the catalytic activity of yeast topoisomerase II was analyzed with the use of two methods: the relaxation of supercoiled plasmid DNA as well as decatenation of kinetoplast DNA in vitro. With the use of the second method, we exclude the possibility of tricking the activity of the enzyme by drug only through direct drug/DNA interaction (intercalation of the drug to DNA) which can result in the unwinding of DNA^27^.

The relaxation of supercoiled plasmid DNA by yeast enzyme was studied in the presence of different concentrations of compounds with the use of a gel-based assays that allow to determine IC_50_ (Table 2).

**Table 2:** The inhibition activity of the analyzed derivatives is determined by densitometry quantification of the transition from supercoiled to relaxed forms and is expressed in relation to the control.

IC_50_ ± SD μM	
Group (I)	
** IKE1**	13.4 ± 1.1
** IKE2**	13.3 ± 1.1
**IKE3**	13.2 ± 1.1
** IKE4**	13.3 ± 1.2
** IKE5**	7.7 ± 1.1
** IKE6**	10.9 ± 1.1
**IKE7**	13.4 ± 1.1
** IKE8**	15.2 ± 1.2
** C-1305**	18.2 ± 1.4
Group (II)	
** IKE9**	14.3 ± 1.1
**IKE10**	15.5 ± 1.3
** IKE11**	14.9 ± 1.1
** IKE12**	10.9 ± 1.1
** IKE13**	6.2 ± 1.2
** IKE14**	14.7 ± 1.1
** C-1330**	13.7 ± 1.1
** C-1311**	16.6 ± 1.3

The half maximal inhibitory concentration (IC_50_) refers to the concentration of a drug, which inhibited the relaxation at 50%. The experiments were performed at least in three replicates. Representative results demonstrating yeast topoisomerase II inhibition are presented in data set ^28^ and Figure S1 (collected in Supplementary Information, SI).

**Figure 1:**
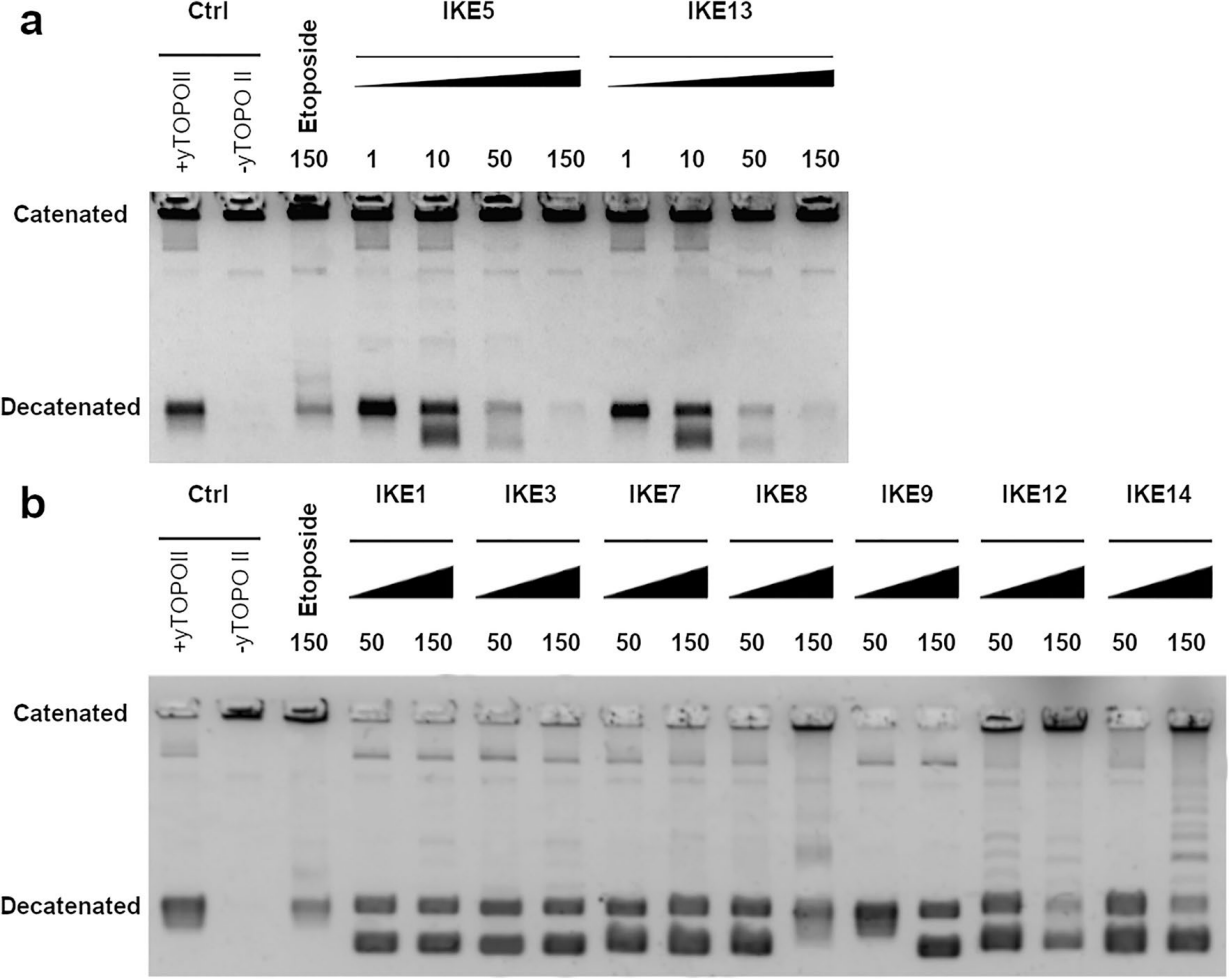
Inhibition of the catalytic activity of purified yeast topoisomerase II by selected compounds as measured decatenation of kinetoplast DNA (catenated). The influence of (**a**) **IKE5, IKE13** at 1, 10, 50, 150 µM and (**b**) **IKE1, IKE3, IKE7, IKE8, IKE9, IKE12** and **IKE14** at 50, 150 µM concentration on the yeast topoisomerase II decatenation ability were tested. Kinetoplast DNA (-yTOPOII) was decatenated by yeast topoisomerase II in the absence (+ yTOPOII) or presence of etoposide (lane 3, 150 µM) or selected compounds. DNA was separated in a 1% agarose gel. The data shown are typical of three independent experiments. The gels were cropped to improve the clarity and conciseness of the presentation. Original gels are presented in Figure S4 (SI).

The IC_50_ values determined for **IKE1-IKE14** against yeast topoisomerase II were compared with those determined for starting compounds **C-1305** and **C-1311** , which are well-known human topoisomerase II (hTOPOII) poisons that inhibit the enzyme through the formation of TOPO II-stabilizing complexes, along with intercalation with DNAs^14,15^. The ability of both derivatives to inhibit yTOPOII relaxation activity was also previously established^16^. The most effective inhibitors towards yeast topoisomerase II synthesized by us were **IKE5** from the group (I) and **IKE13** from the group (II). As previously reported, IC_50_ for **C-1305** and **C-1311** against human topoisomerase II-mediated relaxation was determined at a concentration of 2.5 μM and 6.5 μM, respectively^14,15^. In our hands, the yeast equivalent of this enzyme was much less sensitive to both anticancer compounds (Table 2). Moreover, yTOPOII-relaxation inhibitory activities of the compounds **IKE5** and **IKE13** were found to be in accordance with the results when kinetoplast DNA was used as a substrate for decatenation (Fig. 1A). Both compounds **IKE5** and **IKE13** were more effective in inhibiting decatenation than the other derivatives (Fig. 1B). Although no complete inhibition of decatenation activity was observed, in the presence of compounds **IKE1, IKE3, IKE7, IKE8, IKE9**, and **IKE12** as well as **IKE14** more decatenation products (partially decatenated mini circles) were observed than for positive control. Results indicated some decatenation activity perturbations.

Studies using purified topoisomerase II from yeast confirmed the ability of **IKE5** and **IKE13** to inhibit the catalytic activity of topoisomerase II.

**Figure 2:**
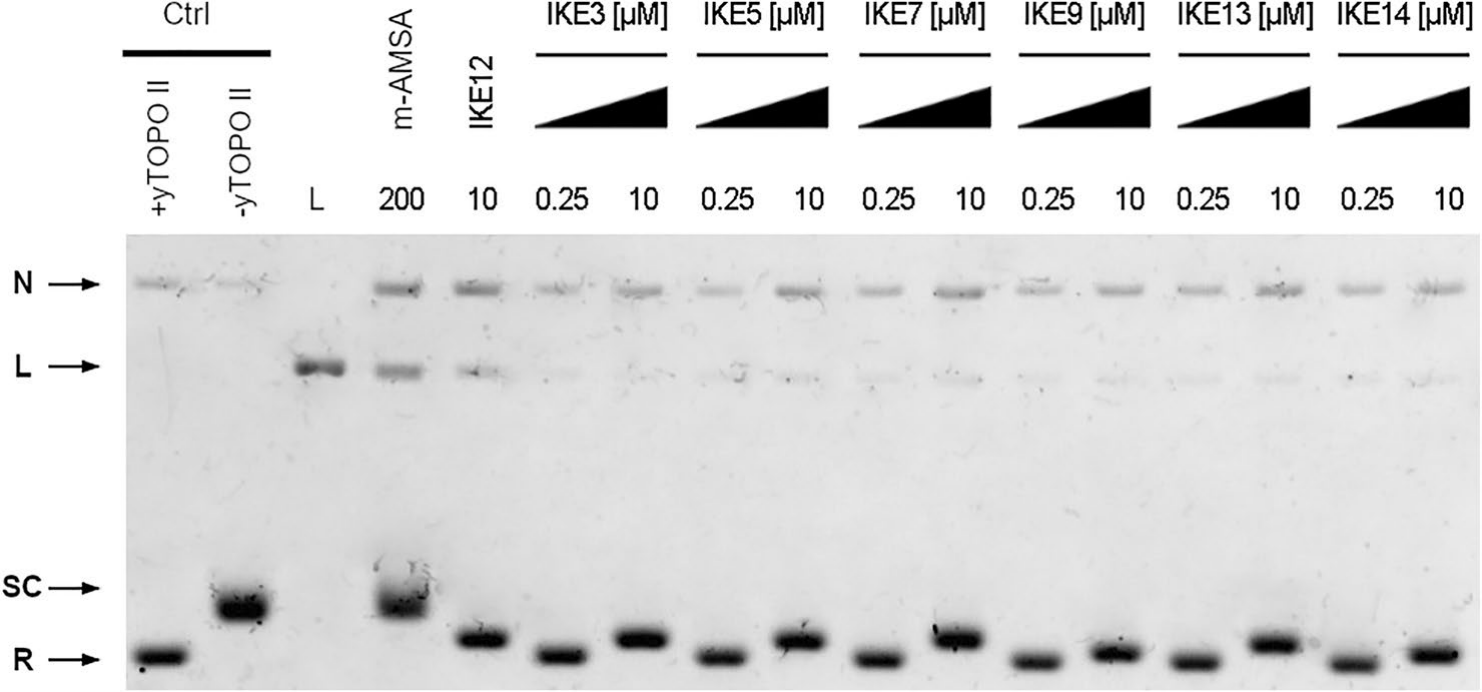
The influence of **IKE3, IKE5, IKE7, IKE9, IKE12, IKE13**, and **IKE14** on the formation of covalent DNA-yeast topoisomerase II complexes. Supercoiled pBR322 plasmid DNA (lane 2, -yTOPOII) was incubated with purified yeast topoisomerase II in the absence (lane 1, + yTOPOII) or in the presence of 200 µM m-AMSA (lane 4) or with 0.25 µM or 10 µM of selected compounds (lanes 5–17). After forming DNA/yeast topoisomerase II complexes, proteinase K was used for digestion. Subsequently, the various topological forms of DNA were separated in a 1.2% agarose gel containing 0.1 µg mL^−1^ ethidium bromide. The data shown are typical of three independent experiments. Lane 3, linearized pBR322 DNA. SC, supercoiled DNA; R, relaxed DNA; L, linear DNA; N, nicked circular DNA. The gel was cropped to improve the clarity and conciseness of the presentation. Original gels are presented in Supplementary Fig. S4.

To analyze whether newly synthesized compounds, similar to the 5-[(2-aminoethyl)amino]-8-hydroxy-6*H*-imidazo[4,5,1-de]acridin-6-one (**IKE12**) and **C-1311** (starting compound)^16^, may also influence the formation of covalent DNA–yeast topoisomerase II complexes we have performed linearization assay tests (Fig. 2).

As we demonstrated earlier, compounds **IKE12** and **C-1311** (the starting compound) with a similar structure to the investigated, impact the formation of covalent complexes between pBR322 plasmid DNA and yeast topoisomerase II^16^. The same mode of action is also known for compound **C1305** (the starting compound), inhibiting human topoisomerase II^15^. As it was expected all analyzed by us compounds indicated the formula of yeast enzyme-stabilizing complex. However, this effect was reduced compared to **IKE12**^16^.

### Selected compound’s interaction with DNA

**Figure 3:**
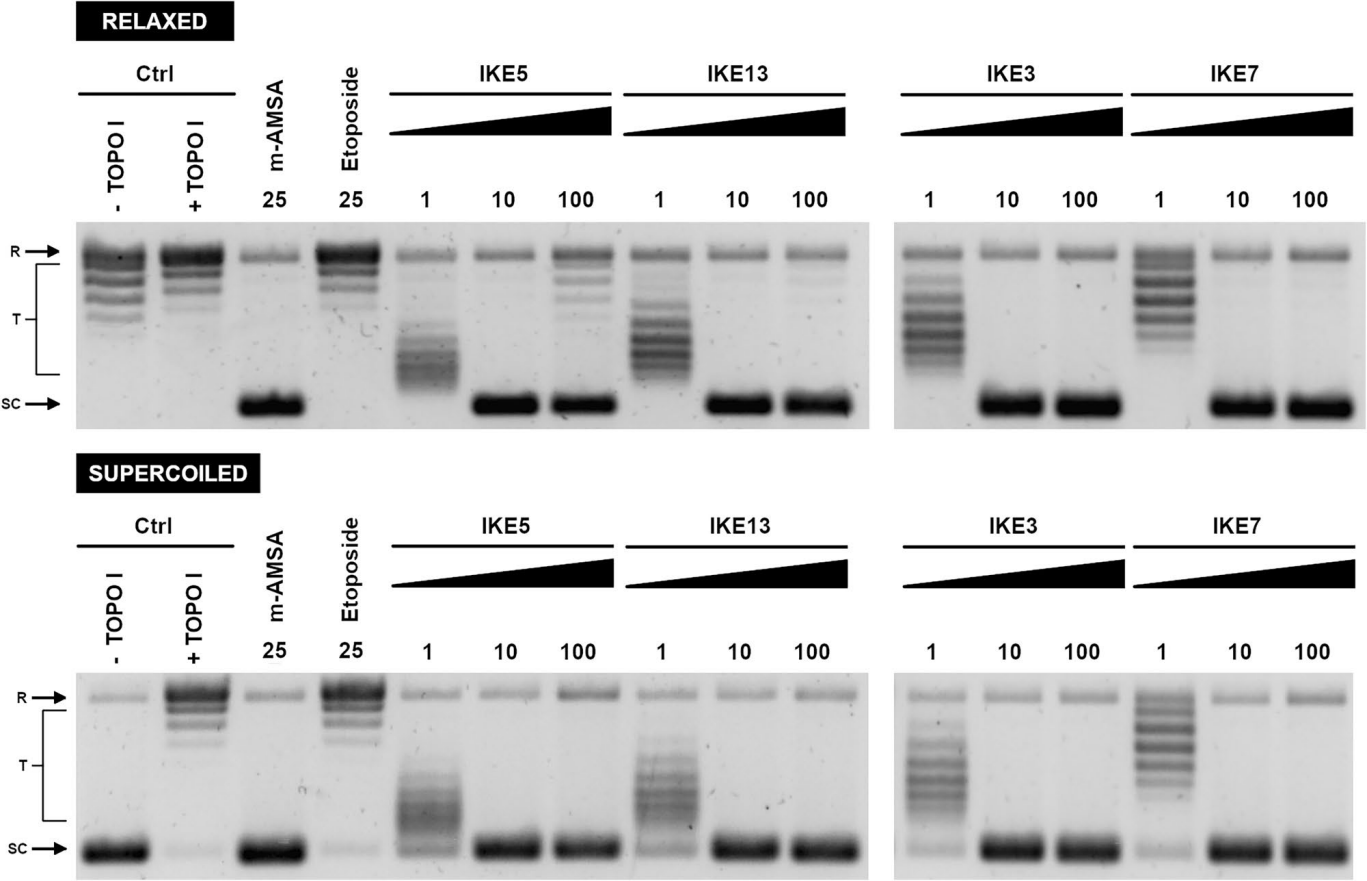
DNA Unwinding Assay. Supercoiled (or relaxed) pBR322 plasmid DNA (lane 1,—Wheat germ TOPO I) was incubated in the absence (lane 2, + Wheat germ TOPO I) or presence of analyzed compounds **IKE3, IKE5, IKE7**, and **IKE13** prior to incubation with wheat germ TOPO I. The ability to intercalate into DNA was investigated. m-AMSA was used to show the ability of intercalation, etoposide was used as an example of a non-intercalator. SC, supercoiled DNA; R, relaxed DNA; T, DNA topoisomers. DNA was separated in a 1% agarose gel. The data shown are typical of three independent experiments. The gels were cropped to improve the clarity and conciseness of the presentation. Original gels are presented in Supplementary Fig. S4.

The ambiguous results obtained for decatenation assay prompted us to conduct additional experiments on the possible compounds intercalation role in yeast topoisomerase II inhibition by performing DNA unwinding assays (Fig. 3).

As indicated in Fig. 3, selected compounds **IKE3, IKE5, IKE7**, and **IKE13** inhibited TOPO I-mediated DNA unwinding in a dose-dependent manner. Both derivatives' behaviour seemed to be the same as for m-AMSA, an eminent DNA intercalating human TOPOIIα poison^29^. Thus, the probable mode of action of analyzed by us derivatives in the context of the importance of the intercalation step could be similar.

**Figure 4:**
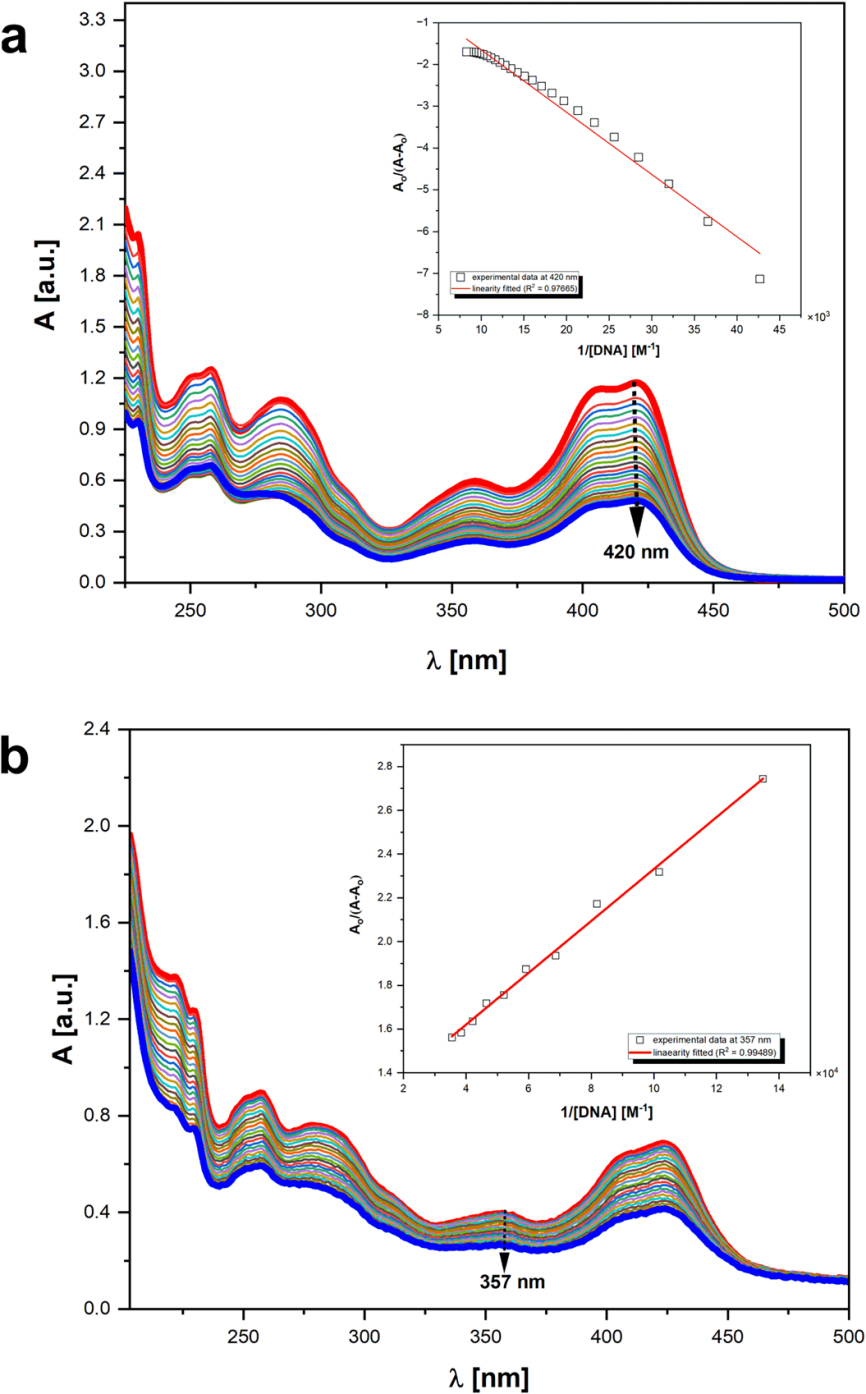
Absorption spectra of **IKE1** (**a**) and **IKE8** (**b**) in the presence of increasing amounts of *CT*-DNA. Arrows indicate that absorbance changes upon increasing *CT*-DNA concentrations. Inset: plot of Ao/(A–Ao) = f(1/[DNA]) established as a result of both compounds’ interactions through titration with *CT*-DNA in Tris–HCl buffer (5 mM/50 mM NaCl; pH 7.43).

Due to the probable role of intercalation of analyzed compounds into DNA in enzyme inhibition, we have decided to perform a more detailed analysis of selected compounds' interaction with calf thymus DNA (*CT*-DNA) by UV–Vis absorption spectroscopy. The effect of varying concentrations of *CT*-DNA on the absorption spectra of **IKE1** and **IKE8** is shown in Fig. 4 and for the other studied compounds **IKE3, IKE7, IKE9, IKE14** is shown in Figs. S14–S15 of SI. With an increasing concentration of *CT*-DNA, the absorption bands of the compounds were affected, resulting in the tendency of hypochromism. The strong absorption of these compounds in the near UV region (240–325 nm) is attributed to the aromatic system^30^. The binding compounds to *CT*-DNA caused hypochromism. This hypochromic effect is thought to be due to the interaction between the electronic states of the intercalating chromophore and those of the DNA bases and caused the conformational changes of *CT*-DNA^31–33^ Hypochromism results from the contraction of the helix of *CT*-DNA. It was observed that a continuous decrease in the absorbance of acridines was followed by the gradually increasing concentration of *CT*-DNA in the solution. A slight bathochromic shift (3–5 nm) was observed with increasing concentrations of *CT*-DNA for **IKE1, IKE8,** and **IKE14** (Figs. S7, S10 and S13 of SI). These spectral characteristics (Table S1 of SI) could indicate their stronger binding to *CT*-DNA than those observed for **IKE7**, **IKE3**, and **IKE9**, respectively. Changes in the position of analyzed compounds' absorption bands as a result of their interaction with DNA are presented in Table S1.

Taking all results together for compounds analyzed by us we can observe intercalator-induced topoisomerase II-mediated DNA cleavage.

### Antifungal activity

**Figure 5:**
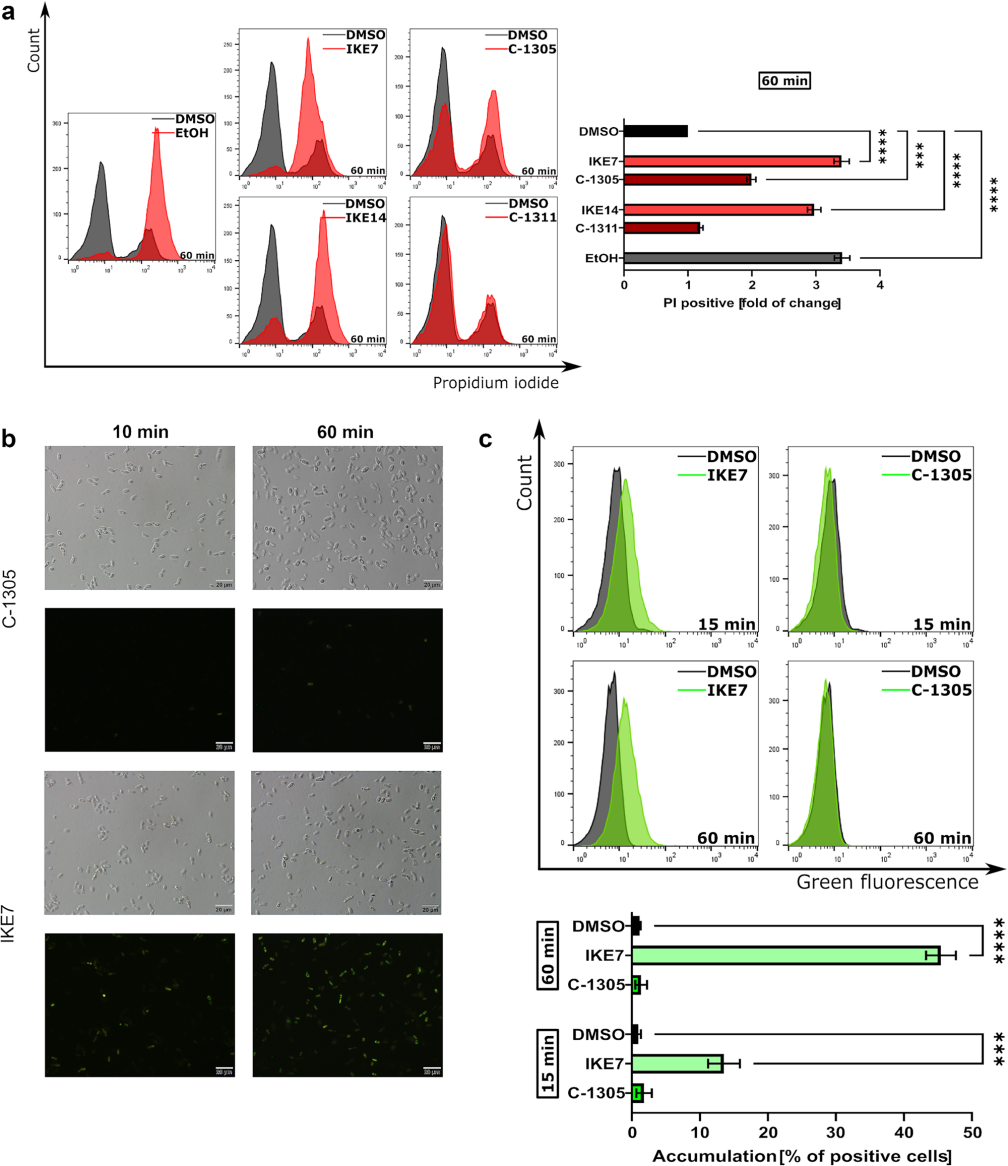
(**a**) Representative histograms and quantification of flow cytometry analysis of fungi labeled with 20 µg mL^−1^ of propidium iodide to detect membrane permeability in *S. cerevisiae* ATCC 9763 cells after treatment with **IKE7, IKE14, C-1305**, and **C-1311** at concentration 64 µg mL^−1^. Error bars represent the mean ± (SD) of the data collected from three independent events. To determine the statistical significance, a one-way analysis of variance (ANOVA) test was conducted. (**b**) Fluorescence microscopic analysis of uptake and accumulation of **IKE7** and **C-1305** in *S. cerevisiae* ATCC 9763 cells. Cells were suspended in phosphate-buffered saline and incubated in the presence of compounds in 100 µM concentration for an appropriate period. Scale bars correspond to 20 µm. (**c**) Representative histograms and quantification of flow cytometry analysis for the accumulation of **IKE7** and **C-1305** at concentration 64 µg mL^-1^ in *S*. *cerevisiae* ATCC 9763 cells. Incubation times of 15 and 60 min were considered. Error bars represent the mean ± (SD) of the data collected from three independent events. To assess statistical significance, a one-way analysis of variance (ANOVA) test was performed.

**IKE1-IKE14** derivatives were tested for their in vitro antifungal activity against five reference strains. Minimal inhibitory concentrations (MICs) of the studied compounds determined by the microplate serial dilution method are shown in Table 3. To establish the possible mode of action we analyzed the killing activity and determined the minimal fungicidal concentrations (MFCs) for selected derivatives as well as performed some flow cytometry analysis with propidium iodide uptake as a cell viability indicator (Fig. 5a).

**Table 3:** Antifungal activity of analyzed derivatives against reference strains. MIC_90_, minimal inhibitory concentrations at which 90% of cells were inhibited.

Compound	*MIC_90_ µg mL^−1^					MFC µg mL^-1^
	*Saccharomyces cerevisiae* ATCC 9763	*Candida glabrata* ATCC 90030	*Candida krusei* ATCC 6258	*Candida parapsilosis* ATCC 22019	*Candida albicans* ATCC 10231	*Saccharomyces cerevisiae* ATCC 9763
Group (I)						
** IKE1**	16	16	32	64	> 64	128
** IKE2**	32	32	32	> 64	> 64	128
** IKE3**	16	32	> 64	> 64 (128)	> 64	64
** IKE4**	32	> 64	> 64	> 64	> 64	-
** IKE5**	32	64	64	64	> 64	-
** IKE6**	32	64	> 64	64	> 64	-
** IKE7**	32	64	64	32	> 64 (128)	64
** IKE8**	32	64	> 64	32	> 64	-
** C-1305**	> 64	> 64	> 64	> 64	> 64	-
Group (II)						
** IKE9**	16	64	> 64	> 64	> 64	64
** IKE10**	64	> 64	> 64	> 64	> 64	-
** IKE11**	64	> 64	> 64	64	> 64	-
** IKE12**^16^	64	> 64 (128)	> 64	> 64	> 64	-
** IKE13**	64	> 64	> 64	64	> 64	-
** IKE14**	32	> 64	> 64	64	> 64	-
** C-1311**	> 64	> 64	> 64	> 64	> 64	-
** C-1330**	32	64	> 64	> 64	> 64	64
** Amphotericin B**	0.5	1	1	1	0.5	2

MFC, a minimal fungicidal concentration that kills 99% of cells. * > Means no activity at the concentration mentioned. For some compounds, higher concentrations were used to determine MIC_90_ (in rounded brackets). In this assay, the MIC_90_ and MFC values of amphotericin B were recorded as positive control. The experiments were performed at least in five replicates.

In contrast to initial compounds **C-1305** and **C-1311** some newly synthetized derivatives exhibited moderate antifungal activity. Comparison of triazoloacridinones (**IKE1-8**) and imidazoacridinones (**IKE9-14**) antifungal activity showed better activity of the former. Our results also indicated that the mode of action of those compounds is fungicidal. The most active compounds from the first group were chosen to test antifungal activity against *C. glabrata* clinical strains, fluconazole-resistant^34^. Results showed that the addition of l-lysine moiety to the structure of **IKE7** compound improved its antifungal activity and allowed to overcome fluconazole resistance (Table 4).

**Table 4:** Antifungal activity of selected compounds against fluconazole-resistant clinical strains in comparison with *C. glabrata* ATCC 90030.

Compound	*MIC_90_ µg mL^−1^				
	*C. glabrata* *CZD 373*	*C. glabrata CZD 377*	*C. glabrata CZD 513*	*C. glabrata* *Gd 310*	*C. glabrata* ATCC 90030
**IKE1**	> 64	> 64	> 64	> 64	16
**IKE2**	> 64	> 64	> 64	> 64	32
**IKE3**	> 64	> 64	> 64	> 64	32
**IKE5**	> 64	> 64	> 64	> 64	64
**IKE6**	> 64	> 64	> 64	> 64	64
**IKE7**	64	32	32	32	64
**Fluconazole**	> 256	> 256	> 256	> 256	32

* > Means no activity at the concentration mentioned. The experiments were performed at least in five replicates.

Our results also indicated that the higher antifungal activity of **IKE7** against fluconazole-resistant clinical strains might be related to the inability to efflux drugs by multidrug-resistance pumps. We used *S. cerevisiae* AD mutants modified by deleting seven multi drug resistance (MDR) transporters and their transformants overexpressing genes encoding CDR1, CDR2, and MDR1 *C. albicans* drug-efflux pumps^35,36^. Derivatives without lysine conjunction **IKE1-3** did not show any activity against *S. cerevisiae* AD mutants at the tested concentration whereas MIC values for **IKE5, 6** were in the range of 64–32 µg mL^−1^. Interestingly, the MIC values for compounds modified by lysine conjunction, **IKE7**, and **IKE8**, were lower. Furthermore, both the parental strain *S. cerevisiae* AD1-8u^−^ and the pump-expressing strains exhibited similar susceptibility to **IKE7** and **IKE8** compounds, indicating that the antifungal activity was not affected by the expression of certain efflux pumps in the strains (Table 5).

**Table 5:** Antifungal activity of **IKE1-3**, **IKE5, 6,** and derivatives modified by lysine conjunction, **IKE7**, and **IKE8**, against *S. cerevisiae* AD mutants and ATCC strain.

Compound	*MIC_90_ µg mL^−1^ determined after 48 h				
	*S. cerevisiae* AD1-8u^-^	*S. cerevisiae* AD-CDR1-GFP	*S. cerevisiae* AD-CDR2-GFP	*S. cerevisiae* AD-MDR1-GFP	*S. cerevisiae* ATCC 9763
**IKE1**	> 64	> 64	> 64	> 64	> 64
**IKE2**	> 64	> 64	> 64	> 64	> 64
**IKE3**	> 64	> 64	> 64	> 64	> 64
**IKE5**	32	32	32	32	64
**IKE6**	32	64	32	32	> 64
**IKE7**	16	16	16	16	32
**IKE8**	16	16	16	16	32

MIC_90_ was determined in assay with YNB medium supplemented with yeast synthetic drop-out medium and uracil after 48 h incubation at 30 °C. * > Means no activity at the concentration mentioned. The experiments were performed at least in five replicates.

Further analysis indicated that the antifungal activity of **IKE7** was related to compound accumulation in the cells (Fig. 5b,c).

### The influence on fungal virulence factors

The ability of fungal cells to perform morphological transformation as well as biofilm formation on abiotic (prostheses, catheters) and biotic (mucous membranes) surfaces are important factors for virulence^37,38^. To check whether the presence of analyzed compounds affects *C. albicans* ATCC 10231 yeast-to-mycelium transformation, fungal cells were incubated under conditions stimulating this process and in the presence of selected derivatives. The results were evaluated by light microscopy, only hyphal inhibitors (**IKE1, IKE3, IKE7, IKE9, IKE14**) and controls are shown (Fig. S16 of SI). Our results indicated that compounds **IKE1, IKE3**, and **IKE7** were able to completely block hyphal formation in Spider medium. To examine the inhibitory effects against *C. albicans* biofilms, we used a microtiter-plate biofilm model (Fig. S17 of SI). Compounds **C-1330, IKE1, IKE2**, and **IKE9** were the only ones that inhibited *C. albicans* ATCC10231 biofilm formation. Surprisingly, although synthesized by us derivatives exhibited no antifungal activity against *C. albicans* (MICs > 64 μg mL^−1^, Table 3) our results indicated that in the presence of selected compounds, some candidal virulence factors might be strongly affected.

### Cytotoxicity of selected derivatives

To understanding the balance between the effectiveness of the analyzed compounds against fungi and their impact on non-target human cells, selected derivatives were screened for their in vitro antiproliferative activity against mammalian cell lines, including normal human embryonic cells (HEK293), and human liver cancer cells (HepG2) using colorimetric MTT ((3-(4,5-dimethylthiazol-2-yl)-2,5-diphenyltetrazolium bromide) assay. The results were calculated after 72 h of treatment and expressed as a half-inhibitory growth concentration (EC_50_) (Table 6).

**Table 6:** The in vitro antiproliferative activity of selected compounds against human embryonic kidney cells (HEK-293) and human liver cancer cells (HEPG2) was evaluated after 72 h of treatment.

Compound	HepG2 EC_50_ ± SD μM	HEK293 EC_50_ ± SD μM
Group (I)		
** IKE1**	5.46 ± 0.41	3.96 ± 0.38
** IKE2**	6.83 ± 0.13	3.58 ± 0.27
** IKE3**	5.39 ± 0.71	7.51 ± 1.17
** IKE7**	12.19 ± 1.21	27.61 ± 1.67
** IKE8**	16.15 ± 0.63	12.57 ± 1.73
** C-1305**	5.07 ± 0.48	0.12 ± 0.05
Group (II)		
** IKE9**	4.82 ± 0.63	4.14 ± 0.85
** IKE12**^16^	0.364 ± 0.013	0.550 ± 0.125
** IKE14**	4.32 ± 0.43	4.49 ± 0.23
** C-1311**^16^	0.129 ± 0.037	0.008 ± 0.007
** C-1330**	4.80 ± 0.70	4.31 ± 0.23

The results are presented as EC_50_ ± SD (µM) and were obtained from three replicate experiments.* EC_50_—the half-maximal inhibitory concentration.

Compounds differ in their cytotoxic activity against human cells. Initial compounds, **C-1305, C-1311**^16^ showed the greatest cytotoxicity whereas the most effective antifungal compound synthetized by us, **IKE7** exhibited the lowest suppressive effect on HEK293 cells. To estimate the selectivity concerning mammalian cells mycostatic selectivity index (MSI) for *S. cerevisiae* cells was calculated as the ratio of EC_50_–MIC_90_ values after converting the MIC_90_ value to micromolar concentrations. The results are presented in Table 7.

**Table 7:** Mycostatic selectivity index values were determined for *S. cerevisiae* ATCC 9763 in relation to mammalian cell lines HEK293 and HEPG2.

Compound	EC_50_HEK293/MIC_90_	EC_50_HEPG2/MIC_90_
**IKE1**	0.083	0.114
**IKE2**	0.041	0.078
**IKE3**	0.165	0.118
**IKE7**	0.389	0.172
**IKE8**	0.171	0.220
**C-1305**	< 0.001	< 0.027

Positive mycostatic selectivity index values (MSI = EC_50_/MIC_90_) were obtained for all selected triazoloacridinone derivatives. The highest level of selectivity was obtained for **IKE7** and **IKE8**.

## Discussion

The emergence of drug resistance among various fungal pathogens has highlighted the urgent need to explore alternative approaches to drug design. While topoisomerases II have been recognized as molecular targets for several chemotherapies, their potential as targets against fungal genera has not been explored. Moreover, the barrier of the fungal cell wall blocks the possibility of using the already discovered, effective fungal enzyme inhibitors.

The present study aimed to identify antifungal compounds targeting yeast topoisomerase II (yTOPOII) derived from human topoisomerase II (hTOPOII) poisons **C-1305** and **C-1311**. Both starting compounds are known as cleavable, complex stabilizing drugs. Our previously published results obtained for **C-1311** and **IKE12** also indicated the same mechanism of action for yeast enzyme^16^. Accordingly to newly synthetized compounds the formation of cleavable complexes between yeast topoisomerase II and pBR322 plasmid DNA was also observed. Although all analyzed by us derivatives induced fewer cleavable complexes than m-AMSA or **IKE12** it seemed that the mechanism of action could be the same. The ability of selected compounds to intercalate with DNA was confirmed by performing DNA unwinding assays as well as a detailed UV–Vis spectral analysis. Interestingly, the stimulation of the *CT*-DNA interaction affinity was proved in the case of derivatives with the lysine moiety. The mentioned conclusion was formulated based on the detailed comparative analyses of the results reported herein. The significantly higher values of binding constants (K_bind_) established spectrophotometrically (Table 8), for derivatives **IKE8**, **IKE7**, and **IKE14** bound *CT*-DNA in comparison to those values of K_bind_ determined for their analogs (**IKE1, IKE3, IKE9**), unequivocally prove stronger interaction mode of the first ones to bind with *CT*-DNA.

**Table 8:** Values of binding constants determined for the interaction between acridines (**IKEs**) and *CT*-DNA.

Compound acronyms	K_*bind*_ [M^−1^]	logK_*bind*_	λ_max_ [nm]	R^2^ (correlation)	Isosbestic points (λ [nm])
**IKE1**	1.58·10^4^	4.19	420	0.97665	ND*
**IKE8**	9.68·10^4^	4.98	357	0.99489	ND*
**IKE3**	1.83·10^5^	5.26	408	0.97753	295, 351, 443
**IKE7**	3.80·10^5^	2.17	410	0.99273	450
**IKE9**	1.60·10^4^	4.20	427	0.97634	340, 468
**IKE14**	9.53·10^5^	5.97	432	0.97634	258, 286, 474

Based on the data collected in Table 8, the specific relation can be formulated. **IKE8** derivative interacted with *CT*-DNA 6 times more strongly than the compound **IKE1. IKE7** showed 2 times stronger affinity than **IKE3**. Interestingly, the K_bind_ values obtained for pair **IKE9** and **IKE14** interacted with a biomolecule, identifying 60 times stronger **IKE14**-*CT*-DNA adduct formation. UV–Vis spectroscopy experiments showed bathochromic shifts and hypochromic effects of the absorption spectra of analyzed triazolo- and imidazoacridinones after interaction with *CT*-DNA, indicative of **IKE** derivatives being deeply buried in the *CT*-DNA structure which was also proved by the high K_bind_ values determined. The constant ionic strength and pH (7.43) in the experimental procedure led to indicating that all compounds investigated behaved as *CT*-DNA intercalators. Due to the K_bind_ values reported, DNA was confirmed as a cellular target for all compounds studied. Finally, we found that the intercalation model best suited to reproduce our registered experimental UV–Vis data for all compounds was in analogy to well-known **C-1305** and its affinity to *CT*-DNA^39^. Moreover, we suggested that for **IKE8, IKE7**, and **IKE14** this mode of binding with *CT*-DNA is also supported electrostatically. Thus, for derivatives modified by lysine conjunction (showed higher K_bind_ values) the hybrid type of interactions were suggested: electrostatic as well as intercalation with *CT*-DNA binding. On the other hand, even 60 times stronger binding affinity with *CT*-DNA determined for **IKE14** than **IKE9** does not result in increased inhibitory activity against yTOPOII. Thus, one may conclude that intercalation ability is not the only reason for being effective as an enzyme inhibitor.

The most effective yTOPOII relaxation activity inhibitors are **IKE5** and **IKE13**, although those compounds' antifungal effect seems to be moderate (**IKE5**) or very low (**IKE13**). Additionally **IKE5** was not active against fluconazole-resistant *C. glabrata* cells. Preliminary structure–activity relationships (SARs) show a correlation between in vitro antifungal activities and chemical structures. Overall, compounds containing triazole ring attached to an acridinone core (triazoloacridinones) inhibited fungal growth better than the corresponding analogues contain an imidazole ring (imidazoacridinones). Moreover, the introduction of lysine moiety within the diaminoalkyl side chain of triazoloacridinones resulted in greater antifungal activity and decreased cytotoxicity. The demonstrated killing activity of the examined derivatives, which increases the likelihood of quickly defeating the infection and decreases the probability of persistent or recurrent infection and resistance development, is also their undoubted advantage. Moreover, triazoloacridinone combined with lysine moiety (**IKE7**) exhibited antifungal activity against *C. glabrata* fluconazole-resistant cells and was not recognised as a substrate by *C. albicans* CDR1p, CDR2p, and MDR1p efflux pumps. Lysine conjunction also resulted in increased uptake and accumulation of **IKE7** in fungal cells.

Our results indicate the possibility of using triazoloacridinone derivatives conjugated with lysine moiety as efficient antifungal agents capable of overcoming fluconazole resistance in *C. glabrata*. However, further research is required to enhance their selectivity. Compound **IKE7** is the most interesting, for which a higher level of MSI using HEK293 cells was obtained.

## Materials and methods

### Chemical synthesis

All compounds described in this work were obtained as hydrochlorides, with a purity of 95–99%. The confirmation of their structures relied on spectral techniques: mass spectrometry ESI–MS, as well as proton and carbon magnetic resonance. The compounds’ purity was determined through the application of thin-layer chromatography (TLC) and high-performance liquid chromatography. All of the synthesized derivatives are hygroscopic. The determination of melting points was conducted using a Stuart SMP30 capillary apparatus, and the values obtained were left uncorrected. The ^1^H NMR and ^13^C NMR spectra were captured using a Varian VXR-S spectrometer operating at a frequency of 500 MHz. Chemical shifts are expressed in δ units, measured in parts per million (ppm), and referenced downfield from internal tetramethylsilane (TMS). The NMR abbreviations utilized in this study include: br.s for broad signal, s for singlet, d for doublet, dd for doublet of doublets, t for triplet, q for quartet, quint for quintet, and m for multiplet. The elemental analyzes' outcomes for individual elements were found to be within a range of ± 0.4% compared to the corresponding theoretical values.

### General procedure for the synthesis of IKE1-IKE6

In a dry 3 mL of DMA suspension, 4.7 mmol of the corresponding amine was introduced. The mixture was then stirred and heated at 60 °C for 3 h after adding 5-chloro-8-methyl-6H-[1–3]-triazolo[4,5,l-de]acridin-6-one (1.5 mmol). Following the reaction, 115 mL of chloroform and water mixture 15:8 (by volume) was combined, and the resulting mixture was vigorously shaken. After separating the organic layer, an additional 75 mL of water was added, and the solution was acidified using l-lactic acid. Subsequently, the water layer was separated, and 1 M NaOH was added to make it basic. The solution was then extracted with chloroform. The organic layer obtained from the extraction was evaporated and dissolved in methanol. Eventually, it was prepared as dihydrochlorides by adding an ether/HCl mixture.

5-[-(3′-Dimethylamino)propylamino]-8-methyl-6*H*-[1–3]-triazolo[4,5,l-*de*]acridin-6-one (**IKE1**).

3-(Dimethylamino)-1-propylamine and 5-chloro-8-methyl-6H-[1–3]-triazolo[4,5,l-*de*]acridin-6-one was used;

Yield 72%; m.p. 180–181 °C;

^1^H NMR (DMSO-*d*_*6*_) δ: 10.98 (br.s, 1H, NH^+^), 9.26 (t, *J* = 5.8 Hz, 1H, 5-*NH*), 8.25–8.29 (m, 2H), 8.04 (s, 1H), 7.76 (d, *J* = 8.3 Hz, 1H), 7.16 (d, *J* = 9.2 Hz, 1H), 3.66 (q, *J* = 6.7 Hz, 2H, NHCH_2_CH_2_), 3.18 (m, 2H, CH_2_CH_2_N(CH_3_)_2_), 2.76 (s, 6H, N(CH_3_)_2_), 2.51 (s, 3H, CH_3_Ar), 2.12 (quint, *J* = 7.3 Hz, 2H, NHCH_2_CH_2_CH_2_N(CH_3_)_2_).

^13^C NMR (DMSO-*d*_*6*_) δ: 177.42, 152.73, 136.94, 135.61, 135.42, 133.16, 132.00, 129.33, 127.46, 124.90, 115.73, 112.41, 99.84, 54.51, 42.43, 34.48, 24.31, 21.40.

ESI–MS m/z: [M + H]^+^ calcd. for C_19_H_21_N_5_O_1_ 335.4, found 336.2

HPLC 96.34%

5-[-(3′-Diethylamino)propylamino]-8-methyl-6H-[1–3]-triazolo[4,5,l-*de*]acridin-6-one (**IKE2**).

3-(Diethylamino)-1-propylamine and 5-chloro-8-methyl-6H-[1–3]-triazolo[4,5,l-*de*]acridin-6-one was used;

Yield 69%; m.p. 194–195 °C;

^1^H NMR (DMSO-*d*_*6*_) δ: 10.42 (br.s, 1H, NH^+^), 9.37 (t, *J* = 6.3 Hz, 1H, 5-*NH*), 8.39 (d, *J* = 8.3 Hz, 1H), 8.35 (d, *J* = 9.3 Hz, 1H), 8.19 (s, 1H), 7.84 (d, *J* = 9.7 Hz, 1H), 7.25 (d, *J* = 9.3 Hz, 1H), 3.69 (q, *J* = 6.8 Hz, 2H, NHCH_2_CH_2_), 3.10–3.18 (m, 6H, CH_2_CH_2_N(CH_2_CH_3_)_2_), 2.54 (s, 3H, CH_3_Ar), 2.10 (quint, *J* = 7.3 Hz, 2H, NHCH_2_CH_2_CH_2_N(CH_2_CH_3_)_2_), 1.22 (t, *J* = 7.3 Hz, 6H, NHCH_2_CH_2_CH_2_N(CH_2_CH_3_)_2_).

^13^C NMR (DMSO-*d*_*6*_) δ: 177.51, 155.78, 136.99, 135.68, 135.45, 133.12, 132.05, 129.40, 127.54, 124.95, 115.78, 112.44, 99.87, 48.31, 46.65, 29.49, 23.54, 21.40, 8.95.

ESI–MS m/z: [M + H]^+^ calcd. for C_21_H_25_N_5_O_1_ 363.4, found 364.3

HPLC 98.98%

5-[-(3′-Dimethylamino)propylamino]-8-methoxy-6H-[1–3]-triazolo[4,5,l-*de*]acridin-6-one (**IKE3**).

3-(Dimethylamino)-1-propylamine and 5-chloro-8-methoxy-6H-[1–3]-triazolo[4,5,l-*de*]acridin-6-one was used;

Yield 77%; m.p. 190–191 °C;

^1^H NMR (DMSO-*d*_*6*_) δ: 10.82 (br.s, 1H, NH^+^), 9.29 (t, *J* = 6.1 Hz, 1H, 5-*NH*), 8.36 (d, *J* = 8.8 Hz, 1H), 8.30 (d, *J* = 8.8 Hz, 1H), 7.71 (d, *J* = 2.9 Hz, 1H), 7.56 (dd, *J*_*1*_ = 2.9 Hz, *J*_*2*_ = 8.8 Hz, Hz, 1H), 7.18 (d, *J* = 9.3 Hz, 1H), 3.93 (s, 3H, OCH_3_), 3.68 (q, *J* = 6.8 Hz, 2H, NHCH_2_CH_2_), 3.18 (m, 2H, CH_2_CH_2_N(CH_3_)_2_), 2.77 (s, 6H, CH_2_CH_2_N(CH_3_)_2_), 2.12 (quint, *J* = 7.8 Hz, 2H, NHCH_2_CH_2_CH_2_N(CH_3_)_2_).

^13^C NMR (DMSO-*d*_*6*_) δ: 176.89, 158.16, 152.77, 135.33, 131.39, 129.39, 129.30, 126.24, 122.43, 117.42, 112.27, 109.40, 99.87, 56.15, 54.53, 42.46, 24.29.

ESI–MS m/z: [M + H]^+^ calcd. for C_19_H_21_N_5_O_2_ 351.4, found 352.1

HPLC 96.85%

5-[-(3′-Diethylamino)propylamino]-8-methoxy-6*H*-[1–3]-triazolo[4,5,l-*de*]acridin-6-one (**IKE4**)

3-(Diethylamino)-1-propylamine and 5-chloro-8-methoxy-6*H*-[1–3]-triazolo[4,5,l-*de*] acridin -6-one was used;

Yield 52%; m.p. 232–233 °C;

^1^H NMR (DMSO-*d*_*6*_) δ: 10.13 (br.s, 1H, NH^+^), 9.37 (m, 1H, 5-*NH*), 8.44 (d, *J* = 8.8 Hz, 1H), 8.36 (d, *J* = 8.8 Hz, 1H), 7.80 (d, *J* = 2.0 Hz, 1H), 7.62 (dd, *J*_*1*_ = 2.4 Hz, *J*_*2*_ = 8.8 Hz, Hz, 1H), 7.24 (d, *J* = 8.8 Hz, 1H), 3.95 (s, 3H, OCH_3_), 3.60–3.71 (m, 2H, NHCH_2_CH_2_), 3.09–3.16 (m, 6H, CH_2_CH_2_N(CH_2_CH_3_)_2_), 2.08–2.11 (m, 2H, NHCH_2_CH_2_CH_2_N(CH_2_CH_3_)_2_), 1.22 (t, *J* = 7.3 Hz, 6H, NHCH_2_CH_2_CH_2_N(CH_2_CH_3_)_2_).

^13^C NMR (D_2_O) low solubility δ: 174.19, 156.35, 151.32, 132.78, 127.92, 129.30, 126.07, 123.07, 115.36, 111.76, 106.57, 96.12, 55.00, 48.88, 39.54, 23.01, 8.22.

ESI–MS m/z: [M + H]^+^ calcd. for C_21_H_25_N_5_O_2_ 379.4, found 380.1

HPLC 97.88%

5-[-(3′-Aminopropyl)amino]-8-methoxy-6H-[1–3]-triazolo[4,5,l-*de*]acridin-6-one (**IKE5**)

1,3-Diaminopropane and 5-chloro-8-methoxy-6H-[1–3]-triazolo[4,5,l-*de*]acridin-6-one was used;

Yield 64%; m.p. 138–139 °C;

^1^H NMR (DMSO-*d*_*6*_) δ: 9.36 (t, *J* = 6.1 Hz, 1H, 5-*NH*), 8.42 (d, *J* = 8.8 Hz, 1H), 8.34 (d, *J* = 9.3 Hz, 1H), 8.08 (br.s, 3H, NH_3_^+^), 7.78 (d, *J* = 2.4 Hz, 1H), 7.61 (dd, *J*_*1*_ = 2.9 Hz, J_2_ = 8.8 Hz, 1H), 7.21 (d, *J* = 9.3 Hz, 1H), 3.94 (s, 3H, OCH_3_), 3.68–3.72 (m, 2H, NHCH_2_CH_2_CH_2_NH_2_), 2.91–2.94 (m, 2H, NHCH_2_CH_2_CH_2_NH_2_), 1.97–2.03 (m, 2H, NHCH_2_CH_2_CH_2_NH_2_).

^13^C NMR (DMSO-*d*_*6*_) δ: 177.11, 158.37, 152.51, 134.94, 130.84, 129.02, 128.19, 125.87, 121.89, 116.56, 111.28, 108.50, 99.07, 54.86, 48.22, 39.57, 37.04, 26.95.

ESI–MS m/z: [M + H]^+^ calcd. for C_17_H_17_N_5_O_2_ 323.3, found 324.2

HPLC 98.6%

5-[-(3′-Aminopropyl)amino]-8-methyl-6*H*-[1–3]-triazolo[4,5,l-*de*]acridin-6-one (**IKE6**)

1,3-Diaminopropane and 5-chloro-8-methyl-6*H*-[1–3]-triazolo[4,5,l-*de*]acridin-6-one was used;

Yield 60%; m.p. 212–213 °C;

^1^H NMR (DMSO-*d*_*6*_) δ: 9.31 (t, *J* = 6.1 Hz, 1H, 5-*NH*), 8.29–8.34 (m, 2H), 8.15 (br.s, 3H, NH_3_^+^), 8.11 (s, 1H), 7.79 (dd, *J*_*1*_ = 1.9 Hz, *J*_*2*_ = 8.3 Hz, 1H), 7.18 (d, *J* = 9.3 Hz, 1H), 3.66–3.70 (m, 2H, NHCH_2_CH_2_CH_2_NH_2_), 2.91–2.95 (m, 2H, NHCH_2_CH_2_CH_2_NH_2_), 2.52 (s, 3H, CH_3_-Ar), 1.98–2.04 (m, 2H, NHCH_2_CH_2_CH_2_NH_2_).

^13^C NMR (DMSO-*d*_*6*_) δ: 177.30, 152.65, 136.80, 135.46, 135.33, 133.04, 131.88, 129.17, 127.34, 124.78, 115.60, 112.30, 99.74, 36.84, 27.28, 21.40.

ESI–MS m/z: [M + H]^+^ calcd. for C_17_H_17_N_5_O_1_ 307.3, found 308.1

HPLC 98.5%

#### General procedure for the synthesis of *IKE7-IKE8*

Example synthesis **IKE7**

To a stirred solution of N-BOC-Lys(Z)-OH (1 mmol) in DMSO (Dimethylsulfoxide) (10 mL) 1 mmol of NHS (N-hydroxysuccinimide), 1 mmol DCC (N,N′-dicyclohexylcarbodiimide), 0.65 mmol triazoloacridinone derivative (**IKE5**) in the presence of a few drops of TEA. After 24 h of stirring at room temperature, the reaction mixture was filtered, washed with DMSO, and then water was added to the filtrate. The precipitate was filtered off and then dried. Yield 86% (0.37 g). The obtained compound was dissolved in 5 mL of glacial AcOH and 1.5 mL of 33% HBr in acetic acid was added. At ambient temperature, the reaction mixture was stirred for a duration of 0.5 h and then the resulting mixture was poured into approximately 15 mL of diethyl ether and stirred again for another 0.5 h. The precipitate was filtered off, washed with ether, and dried. The residue was purified by column chromatography on silica gel. The initial eluent was CHCl_3_/MeOH/NH_3_ (15:1.5:0.15 by volume) and then (10:2:0.2 by volume), (10:3:0.2 by volume). The purified product in the form of a base, was dissolved in methanol (10 mL) and acidified with HCl/diethyl ether. Next, it was precipitated out with diethyl ether.

*N*′-{3-[(8-methoxy-6-oxo-6H-[1,2,3]triazolo[4,5,1-de]acridin-5-yl)amino]propyl} lysinamide (**IKE7**)

Yield 51% m.p. 159–160 °C;

^1^H NMR (DMSO-*d*_*6*_) δ: 9.42 (t, *J* = 6.1 Hz, 1H, 5-*NH*), 8.87 (t, *J* = 5.4 Hz, 1H, NHC(O)), 8.45 (d, *J* = 8.8 Hz, 1H), 8.35 (d, *J* = 9.3 Hz, 1H), 8.32 (br.s, 3H, NH_3_^+^), 7.98 (br.s, 3H, NH_3_^+^), 7.81 (s, 1H), 7.62 (dd, *J*_*1*_ = 2.9 Hz, *J*_*2*_ = 9.3 Hz, 1H), 7.21 (d, *J* = 9.2 Hz, 1H), 3.96 (s, 3H, OCH_3_), 3.78–3.85 (m, 2H, NHCH_2_CH_2_CH_2_NHC(O)CH(NH_2_)), 3.63–3.67 (m, 1H, NHCH_2_CH_2_CH_2_NHC(O)CH(NH_2_)), 3.27–3.33 (m, 2H, NHCH_2_CH_2_CH_2_NHC(O)CH(NH_2_)), 2.76–2.80 (m, 2H, CHCH_2_CH_2_CH_2_CH_2_NH_2_), 1.85–1.92 (m, 2H, NH_2_CH_2_CH_2_CH_2_NH_2_), 1.73–1.80 (m, 2H, CHCH_2_CH_2_CH_2_CH_2_NH_2_), 1.56–1.62 (m, 2H, CHCH_2_CH_2_CH_2_CH_2_NH_2_), 1.35–1.41 2.76–2.80 (m, 2H, CHCH_2_CH_2_CH_2_CH_2_NH_2_).

^13^C NMR (DMSO-*d*_*6*_) δ: 176.85, 169.10, 158.125, 152.91, 135.18, 131.39, 129.39, 129.30, 126.26, 122.41, 117.37, 112.27, 109.40, 99.26, 56.14, 52.43, 40.68, 38.64, 36.62, 30.74, 29.15, 26.69, 21.70.

ESI–MS m/z: calcd. for C_23_H_29_N_7_O_3_ 451.5, found [M + H]^+^ 452.2, [M − H]^−^450.2

HPLC 99.2%

*N*′-{3-[(8-methyl-6-oxo-6H-[1,2,3]triazolo[4,5,1-de]acridin-5-yl)amino]propyl} lysinamide (**IKE8**)

The method of synthesis was similar to that in the case of derivative **IKE7**: derivative **IKE6** was used; Yield 57%; m.p. 128–129 °C;

^1^H NMR (DMSO-*d*_*6*_) δ: 9.41 (t, *J* = 6.1 Hz, 1H, 5-*NH*), 8.81 (t, *J* = 5.8 Hz, 1H, NHC(O)), 8.41 (d, *J* = 8.3 Hz, 1H), 8.35 (d, *J* = 9.2 Hz, 1H), 8.28 (br.s, 3H, NH_3_^+^), 8.21 (s, 1H), 7.93 (br.s, 3H, NH_3_^+^), 7.85 (dd, *J*_*1*_ = 1.9 Hz, *J*_*2*_ = 8.3 Hz, 1H), 7.20 (d, *J* = 9.3 Hz, 1H), 3.76–3.80 (m, 1H, NHCH_2_CH_2_CH_2_NHC(O)CH(NH_2_)), 3.60–3.66 (m, 2H, NHCH_2_CH_2_CH_2_NHC(O)CH(NH_2_)), 3.28–3.31, (m, 2H, NHCH_2_CH_2_CH_2_NHC(O)CH(NH_2_)), 2.76–2.80 (m, 2H, CHCH_2_CH_2_CH_2_CH_2_NH_2_), 1.87–1.91 (m, 2H, NH_2_CH_2_CH_2_CH_2_NH_2_), 1.74–1.77 (m, 2H, CHCH_2_CH_2_CH_2_CH_2_NH_2_), 1.57–1.60 (m, 2H, CHCH_2_CH_2_CH_2_CH_2_NH_2_), 1.36–1.39 (m, 2H, CHCH_2_CH_2_CH_2_CH_2_NH_2_).

^13^C NMR (DMSO-*d*_*6*_) δ: 177.35, 169.05, 152.83, 136.85, 135.51, 135.26, 133.10, 131.94, 129.30, 127.42, 124.86, 115.67, 112.36, 99.66, 52.44, 40.69, 38.69, 36.67, 30.77, 29.16, 26.74, 21.72, 21.41.

ESI–MS m/z: calcd. for C_23_H_29_N_7_O_2_ 435.5, found [M + H]^+^ 436.2, [M − H]^−^ 434.1

HPLC 98.3%

#### General procedure for the synthesis of IKE9-10 and IKE12-13

The synthesis procedure was based on the previously described one for 5-[(2-aminoethyl)amino]-8-hydroxy-6*H*-imidazo[4,5,1-de]acridin-6-one^16^ compound. In summary, the corresponding amine (13.34 mmol) was added to the suspension of 1-chloro-7-substituted-4-nitro-9(10H)-acridone derivatives (3.44 mmol) in 20 mL of DMSO, and the solution was stirred at room temperature for a duration of 2.5 h. Following this, approximately 50 mL of water was added, and the reaction mixture was subjected to stirring for an additional 0.5 h. The precipitate obtained was collected through filtration and then transferred into water, where it was acidified using dilute hydrochloric acid and stirred for 0.5 h. The undissolved material was filtered out, and the resulting solution was evaporated to a reduced volume. To precipitate the product, acetone (approximately 100 mL) was employed, and the precipitate was subsequently filtered. The obtained derivative, along with 10% Pd/C in catalytic quantities and 40 mL of 96% formic acid, was subjected to hydrogenation by passing gaseous hydrogen through them at room temperature for a period of 24 h. After this time, the catalyst was separated by filtration. To the filtrate, 2–3 mL of concentrated HCl was added, and the mixture was heated at 110 °C for 24 h. Following this step, the formic acid was evaporated, and the resulting residue was heated for 3 h in a mixture of water and methanol with a 1:1 ratio (approximately 50 mL). The solvent was evaporated, and the residue was dissolved in methanol and then acidified with concentrated hydrochloric acid. The product was finally crystallized from diethyl ether.

5-{[(2-dimethylamino)ethyl]amino}-8-methoxy-6H-imidazo[4,5,1-de]acridin-6-one (**IKE9**)

*N*,*N*-Dimethylethylenediamine and 1-chloro-7-methoxy-4-nitro-9(10H)-acridone was used;

Yield 71%; m.p. 283–284 °C;

^1^H NMR (DMSO-*d*_*6*_) δ: 10.10 (s, 1H, N10CH), 9.01–9.12 (m, 1H, 5-*NH*), 8.45 (d, *J* = 9.3 Hz, 1H), 8.12 (d, *J* = 9.3 Hz, 1H), 7.81 (d, *J* = 2.9 Hz, 1H), 7.62 (dd, *J*_*1*_ = 2.9 Hz, *J*_*2*_ = 9.3 Hz, 1H), 7.22 (d, *J* = 9.3 Hz, 1H), 3.93 (s, 3H, OCH_3_), 3.93–3.95 (m, 2H, NHCH_2_CH_2_N(CH_3_)_2_), 3.38–3.41 (m, 2H, NHCH_2_CH_2_N(CH_3_)_2_), 2.88 (s, 6H, N(CH_3_)_2_).

^13^C NMR (DMSO-*d*_*6*_) δ: 177.78, 157.81, 149.43, 136.08, 131.91, 129.15, 128.37, 126.53, 122.46, 118.77, 109.38, 108.37, 102.64, 56.18, 55.03, 42.91, 37.98.

ESI–MS m/z: [M + H]^+^ calcd. for C_19_H_20_N_4_O_2_ 336.3, found 337.1

HPLC 98.1%

5-[(2-aminoethyl)amino]-8-methyl-6*H*-imidazo[4,5,1-de]acridin-6-one (**IKE10**)

Ethylenediamine and 1-chloro-7-methyl-4-nitro-9(10H)-acridone was used;

Yield 61%; m.p. 264–265 °C;

^1^H NMR (DMSO-*d*_*6*_) δ: 9.16 (s, 1H, N10CH), 9.02 (m, 1H, 5-*NH*), 8.30 (d, *J* = 8.3 Hz, 1H), 8.15 (s, 1H), 7.97 (d, *J* = 8.8 Hz, 1H), 7.73 (d, *J* = 8.3 Hz, 1H), 6.84 (d, *J* = 9.2 Hz, 1H), 3.36–3.41 (m, 2H, NHCH_2_CH_2_NH_2_), 2.88–2.90 (m, 2H, NHCH_2_CH_2_NH_2_), 2.51 (s, 3H, Ar-CH_3_).

^13^C NMR (DMSO-*d*_*6*_) δ: 177.98, 149.60, 136.23, 135.99, 135.33, 132.88, 132.14, 128.54, 128.14, 127.25, 125.09, 116.88, 108.26, 102.93, 37.98, 21.30.

ESI–MS m/z: [M + H]^+^ calcd. for C_17_H_16_N_4_O_1_ 292.3, found 293.1

HPLC 98.9%

5-[(2-aminoethyl)amino]-8-hydroxy-6*H*-imidazo[4,5,1-de]acridin-6-one (**IKE12**)

Ethylenediamine and 1-chloro-7-hydroxy-4-nitro-9(10H)-acridone was used;

Yield 59%; m.p. 332–334 °C;

^1^H NMR (DMSO-*d*_*6*_) δ: 10.27 (s, 1H, N10CH), 9.11–9.21 (m, 1H, 5-*NH*), 8.34 (d, *J* = 9.3 Hz, 1H), 8.10 (d, *J* = 8.8 Hz, 1H), 7.98 (br.s, 3H, NH_3_^+^), 7.77 (s, 1H), 7.41 (dd, *J*_*1*_ = 2.9 Hz, *J*_*2*_ = 8.8 Hz, 1H), 7.23 (d, *J* = 9.3 Hz, 1H), 3.77–3.80 (m, 2H, NHCH_2_CH_2_NH_2_), 3.09–3.13 (m, 2H, NHCH_2_CH_2_NH_2_).

^13^C NMR (DMSO-*d6* + TFA) δ: 175.41, 155.52, 150.06, 130.73, 127.74, 125.20, 124.22, 123.99, 121.70, 117.84, 116.87, 111.85, 110.34, 99.33, 39.37, 37.50.

ESI–MS m/z: calcd. for C_16_H_14_N_4_O_2_ 294.3, found [M + H]^+^ 295.1, [M-H]^-^ 293.1

HPLC 98.5%

5-[(2-aminoethyl)amino]-8-methoxy-6H-imidazo[4,5,1-de]acridin-6-one (**IKE13**)

Ethylenediamine and 1-chloro-7-methoxy-4-nitro-9(10H)-acridone was used;

Yield 63%; m.p. 336–337 °C;

^1^H NMR (DMSO-*d*_*6*_) δ: 9.57 (s, 1H, N10CH), 8.97 (m, 1H, 5-*NH*), 8.41 (d, *J* = 8.8 Hz, 1H), 8.25 (br.s, 3H, NH_3_^+^), 8.04 (d, *J* = 9.3 Hz, 1H), 7.75 (d, *J* = 2.9 Hz, 1H), 7.58 (dd, *J*_*1*_ = 2.9 Hz, *J*_*2*_ = 8.8 Hz, 1H), 7.06 (d, *J* = 8.8 Hz, 1H), 3.93 (s, 3H, OCH_3_), 3.78–3.85 (m, 2H, NHCH_2_CH_2_NH_2_), 3.08–3.11 (m, 2H, NHCH_2_CH_2_NH_2_).

^13^C NMR (DMSO-*d*_*6*_) δ: 177.61, 157.72, 149.68, 135.95, 131.91, 129.11, 128.51, 128.39, 126.49, 122.31, 118.69, 109.34, 108.09, 102.60, 56.14, 38.00, 36.95.

ESI–MS m/z: [M + H]^+^ calcd. for C_17_H_16_N_4_O_2_ 308.3, found 309.1

HPLC 96.5%

#### General procedure for the synthesis of IKE11 and IKE14

Example synthesis **IKE11**

To a stirred solution of N- BOC-Lys(Z)-OH (1.23 mmol) in DMSO (15 mL) 1.23 mmol of NHS, 1.23 mmol DCC, 0.81 mmol imidazoacridinone derivative (**IKE10**) in the presence of a few drops of TEA. After 24 h of stirring at room temperature, the reaction mixture was filtered, washed with DMSO, and then water was added to the filtrate. The precipitate was filtered off and then dried. Yield 80%. The obtained compound was dissolved in 5 mL of glacial AcOH and 1.5 mL of 33% HBr in acetic acid was added. The reaction mixture was stirred at room temperature for 0.5 h. The resulting mixture was poured into diethyl ether (~ 15 mL) and then stirred for 0.5 h. The precipitate was filtered off, washed with ether, and dried. The residue was purified by column chromatography on silica gel. The initial eluent was CHCl_3_/MeOH/NH_3_ (15:1.5:0.15 by volume) and then (10:2:0.2 by volume), (10:3:0.2 by volume). The purified product in the form of a base, was dissolved in methanol (10 mL) and acidified with HCl/diethyl ether. Next, it was precipitated out with diethyl ether.

*N*-{2-[(8-methyl-6-oxo-6H-imidazo[4,5,1-de]acridin-5-yl)amino]ethyl}lysinamide (IKE11)

Yield 41%; m.p. 148–149 °C;

^1^H NMR (DMSO-*d*_*6*_) δ: 9.32 (s, 1H, N10CH), 8.94 (t, *J* = 5.8 Hz, 1H, NHC(O)), 8.97 (m, 1H, 5-*NH*), 8.37 (d, J = 8.2 Hz, 1H), 8.30 (br.s, 3H, NH_3_^+^), 8.18 (s, 1H), 8.04 (d, *J* = 8.8 Hz, 1H), 7.97 (br.s, 3H, NH_3_^+^), 7.79 (d, *J* = 8.6 Hz, 1H), 7.01 (d, *J* = 9.0 Hz, 1H), 3.35–3.76 (m, 5H, NHCH_2_CH_2_NHC(O)CH(NH_2_)), 2.73–2.77 (m, 2H, CHCH_2_CH_2_CH_2_CH_2_NH_2_), 2.52 (s, 3H, Ar-CH_3_), 1.70–1.80 (m, 2H, CHCH_2_CH_2_CH_2_CH_2_NH_2_), 1.53–1.59 (m, 2H, CHCH_2_CH_2_CH_2_CH_2_NH_2_), 1.36–1.40 (m, 2H, CHCH_2_CH_2_CH_2_CH_2_NH_2_).

^13^C NMR (DMSO-*d*_*6*_) δ: 177.80, 169.51, 150.10, 136.49, 135.60, 135.26, 132.72, 131.87, 127.76, 127.27, 125.18, 116.99, 108.70, 102.39, 52.42, 49.04, 42.00, 38.56, 30.72, 26.69, 21.64, 21.32.

ESI–MS m/z: calcd. for C_23_H_28_N_6_O_2_ 420.5, found [M + H]^+^ 421.2, [M-H]^-^ 419.2

HPLC 98.4%

*N*-{2-[(8-methoxy-6-oxo-6H-imidazo[4,5,1-de]acridin-5-yl)amino]ethyl}lysinamide (IKE14)

The method of synthesis was similar to that in the case of derivative **IKE11**: derivative **IKE13** was used; Yield 45%; m.p. 164–165 °C;

^1^H NMR (DMSO-*d*_*6*_) δ: 9.31 (s, 1H, N10CH), 8.98 (m, NHC(O)), 8.93 (t, *J* = 5.4 Hz, 1H, 5-*NH*), 8.43 (d, *J* = 9.1 Hz, 1H), 8.29 (br.s, 3H, NH_3_^+^), 8.04 (d, *J* = 9.1 Hz, 1H), 7.94 (br.s, 3H, NH_3_^+^), 7.81 (d, *J* = 2.7 Hz, 1H), 7.60 (dd, *J*_*1*_ = 2.9 Hz, *J*_*2*_ = 9.1 Hz, 1H), 7.00 (d, *J* = 9.0 Hz, 1H), 3.94 (s, 3H, OCH_3_), 3.72–3.80 (m, 1H, NHCH_2_CH_2_NHC(O)CH(NH_2_)), 3.58–3.63 (m, 2H, NHCH_2_CH_2_NHC(O)CH(NH_2_)), 3.38–3.42 (m, 2H, NHCH_2_CH_2_NHC(O)CH(NH_2_)), 2.64–2.75 (m, 2H, CHCH_2_CH_2_CH_2_CH_2_NH_2_), 1.70–1.81 (m, 2H, CHCH_2_CH_2_CH_2_CH_2_NH_2_), 1.52–1.58 (m, 2H, CHCH_2_CH_2_CH_2_CH_2_NH_2_), 1.36–1.39 (m, 2H, CHCH_2_CH_2_CH_2_CH_2_NH_2_).

^13^C NMR (DMSO-*d*_*6*_) δ: 177.38, 169.52, 157.86, 150.17, 135.48, 131.54, 128.87, 127.89, 126.57, 122.29, 118.79, 109.21, 108.60, 102.01, 56.18, 52.41, 42.05, 38.55, 30.71, 26.68, 21.62.

ESI–MS m/z: [M + H]^+^ calcd. for C_17_H_16_N_4_O_2_ 364.4, found 437.2

HPLC 98.0%

### Microbiological tests

#### Microorganisms strains and growth conditions

The following fungal strains were used: *Candida albicans* ATCC 10,231, *Candida glabrata* ATCC 90,030, *Candida krusei* ATCC 6258, *Candida parapsilosis* ATCC 22,019, *Saccharomyces cerevisiae* ATCC 9763, *Candida albicans* clinical isolates^40,41^: B3, B4, Gu4, Gu5, F2, F5, *Candida glabrata* clinical isolates^34^: CZD 373, CZD 377, CZD 513, Gd 310, *Saccharomyces cerevisiae* strains^35,36,42^: AD-MDR1-GFP, AD1-8u^−^, AD-CDR1-GFP, AD-CDR2-GFP. Fungal strains were routinely grown over 18 h at 30 °C in YPG liquid medium (1% m/V yeast extract, 1% m/V peptone, 2% m/V glucose) in a shaking incubator (INFORS HT Bottmingen, Switzerland). For growth on solid media, 1.5% m/V agar was added to the YPG medium. For antimicrobial activity assays RPMI-1640 (Sigma-Aldrich, St. Louis, MO, USA) medium buffered to pH 7.0 was used for all strains except for *Saccharomyces cerevisiae* mutants AD-MDR1-GFP, AD1-8u^−^, AD-CDR1-GFP, AD-CDR2-GFP. *Saccharomyces cerevisiae* mutants were grown in 0.67% *m/V* yeast nitrogen base medium without amino acids, folic acid, p-aminobenzoic acid, with ammonium sulphate (MP Biomedicals, Irvine, CA, USA) supplemented with 2% *m/V* glucose, 0.192% *m/V* yeast synthetic drop-out medium supplement without uracil (Sigma-Aldrich, St. Louis, MO, USA) and 0.0076% *m/V* uracil (Sigma-Aldrich, St. Louis, MO, USA).

#### Antifungal activity assays

Determination of in vitro antifungal activity was performed according to the modified M27-A3 specified by the CLSI^43^. Strains (10^4^ CFU (colony-forming unit) mL^−1^) were grown in the appropriate medium in a 96-wells microplate for 24 h (48 h for *S. cerevisiae* mutants) at 30 °C with or without tested compounds. The optical denticity was measured at 600 nm by a microplate reader (TECAN Spark 10 M, Grödig, Austria). The Minimum Inhibitory Concentration (MIC) was determined as the lowest concentration of the drug showing a minimum 90% reduction in turbidity compared to the drug-free control.

The Minimum Fungicidal Concentration (MFC) was defined as the lowest concentration of the test compound in which no recovery of microorganisms was observed. The MFC was determined as described previously^17^ by spot assay.

#### Accumulation of the compounds in fungal cells

The accumulation of the compounds in fungal cells was examined with the Olympus BX-60 fluorescence microscope (λex = 470–490 nm, λem ≥ 500 nm, lens × 40) equipped with the Olympus XC50 digital camera (Olympus Corporation, Tokyo, Japan). The overnight culture of *S. cerevisiae* ATCC 9763 in YPG medium was subjected to two rounds of washing using sterile phosphate-buffered saline (PBS) and resuspended to achieve a cell density of 2 × 10^7^ CFU mL^−1^. Cells were incubated with **IKE5**, **IKE7**, and **C-1305** compounds at 100 µM concentration for 10–60 min. Following the designated time period, a 1 mL aliquot of the cell suspension underwent a triple-washing procedure using sterile phosphate-buffered saline (PBS). Cells were resuspended in 40 µL 90% (by volume) glycerol and 10% (by volume) of tenfold concentrated PBS. Then 3 µL of inoculum was transferred to a microscopic slide.

#### Morphological changes of fungi

*Candida albicans* ATCC 10,231 cells were cultured in 24-well plates with a glass bottom and incubated with either DMSO or the investigated compounds in RPMI-1640 + 10% (by volume) fetal bovine serum or Spider medium (1% *m/V* nutrient broth (Difco), 1% *m/V* D-mannitol (Sigma-Aldrich, St. Louis, MO, USA), 0.2% *m/V* K_2_HPO_4_, pH 7.2) at 37 °C for 3 h. Following the incubation period, the cells were washed three times with PBS and imaged using a light microscope. Specifically, an LSM 800 inverted laser-scanning confocal microscope from Carl Zeiss (ZEISS, Göttingen, Germany), equipped with a × 63 1.4-NA Plan Apochromat objective from Carl Zeiss, was utilized for imaging.

#### The effect of analyzed compounds on *C. albicans* biofilm formation

*Candida albicans* biofilm formation was analyzed with the use of a microtiter plate-based method according to the previously described protocol^17^.

#### Flow cytometry analysis

To evaluate the change in fungal membrane permeability, the propidium iodide influx assay was utilized. The *Saccharomyces cerevisiae* ATCC 9763 cells were cultured in a YPG medium at a concentration of 10^6^ CFU mL^−1^. These cells were then exposed to the investigated compounds for 60 min. After the incubation period, the cells were collected and washed twice with PBS buffer. Subsequently, the cells were resuspended and incubated with 20 µg mL^−1^ of propidium iodide in PBS. Additionally, the accumulation of the compounds within the cells was measured after 15 and 60 min by assessing the intensity of green fluorescence. A cell suspension was analyzed using the Guava easyCyte 8 cell sorter (Merck Millipore, Hayward, CA, USA)), and the data were processed using FlowJo v10.8.0 software (BD Life Sciences, Franklin Lakes, NJ, USA). Each experiment was independently repeated three times.

### Topoisomerase assays

#### Yeast topoisomerase II relaxation assay and inhibition

The inhibition of yeast topoisomerase II was analyzed according to the relaxation assay method described previously.

#### Formation of cleavable complexes in vitro

Cleavage assay of yeast topoisomerase II was performed according to the method described previously^16^.

#### Decatenation assay

Yeast topoisomerase II (*Saccharomyces cerevisiae*, Inspiralis Limited, UK) ability to decatenate kinetoplast DNA (kDNA) from *Crithidia fasciculata* was examined according to a decatenation assay kit from Inspiralis (Inspiralis Limited, Norwich, UK). In summary, for the experimental procedure, a mixture containing 200 ng of kDNA, 1 mM ATP (Inspiralis Limited, UK), and varying concentrations (ranging from 1 to 150 µM) of the analyzed compounds was prepared with reaction buffer (comprising 4 mM Tris–HCl at pH 7.9, 40 mM KCl, 2 mM MgCl_2_, and 0.8% (by volume) glycerol). The enzymatic reaction was initiated by introducing the enzyme and allowed to proceed at a temperature of 30 °C for a duration of 30 min. Termination of the reaction was achieved by adding 40% (*m/V*) sucrose, along with 100 mM Tris–HCl at pH 8, 10 mM EDTA, and 0.5 mg mL^−1^ of Bromophenol Blue. For the subsequent steps, a two-step extraction was performed using chloroform:isoamyl alcohol (in a ratio of 24:1, by volume) and butanol water. The resulting mixtures were then analyzed on a 1% (w/v) agarose gel in 1 × TAE buffer (Tris–acetate-EDTA buffer, NZYTech, Lisbona, Portugal), with an electrophoresis time of 4 h at 4.5 V cm^−1^. The gel was immersed in GelRed 3 × staining solution (Biotium, Fremont, CA, USA) for a duration of 30 min. Subsequently, it was captured using the Gel Doc XR + Gel Documentation System (Bio-Rad: Hercules, CA, USA) to obtain photographs.

#### Unwinding assay

The kit from Inspiralis (Inspiralis Limited, Norwich, UK) was utilized to assess the compounds' capacity to intercalate into the DNA, which subsequently resulted in the unwinding of the DNA strands. Briefly, supercoiled or relaxed pBR322 plasmid (250 ng) was combined with the tested compounds at concentrations ranging from 1 to 100 µM. This mixture was then supplemented with the reaction buffer comprising 25 mM Tris–HCl (pH 7.9), 0.5 mM EDTA, 0.5 mM DTT (ditiotreitol), 10% (by volume) glycerol, and 25 mM NaCl. The assay was initiated by introducing Wheat germ topoisomerase I, and the mixture was then incubated for 30 min at 37 °C. To stop the reaction, 10 µL of water and 25 µL of butanol were added, followed by centrifugation at 16,100 xg for 1 min. Further, 40% (*m/V*) sucrose, 100 mM Tris–HCl at pH 8, 10 mM EDTA, and 0.5 mg mL^−1^ of Bromophenol Blue were added, and extraction with chloroform:isoamyl alcohol (in a ratio of 24:1, by volume) was performed. The mixtures were then analyzed on a 1% (*m/V*) agarose gel in 1 × TAE buffer for 18 h at 1.1 V cm^−1^. GelRed 3 × staining solution (obtained from Biotium, Fremont, CA, USA) was used to stain the gel for 30 min, and the gel was photographed using the Gel Doc XR + Gel Documentation System (Bio-Rad: Hercules, CA, USA).

### Antiproliferative activity determination

#### Cell culture

HEK-293 (human embryonic kidney) and HEPG2 (human liver cancer) cell lines were obtained from ATCC (Manassas, Virginia, USA). HEK-293 cells were cultured in Dulbecco's Modified Eagle Medium (DMEM), while HEPG2 cells were cultured in Minimum Essential Medium Eagle (MEM). Both cell lines received a supplement of 10% (by volume) fetal bovine serum, 2 mM L-glutamine, and antibiotics: penicillin at a concentration of 62.6 µg mL^−1^ and streptomycin at a concentration of 40 µg mL^−1^. The cells were cultivated in a humidified environment with 5% CO_2_ and 95% air. Regular screening for *Mycoplasma w*as carried out to ensure the integrity of the cell lines. All the reagents utilized in this study were purchased from Corning unless otherwise stated.

#### Cell viability

The assessment of antiproliferative activity was carried out using the (3-(4,5-dimethylthiazol-2-yl)-2,5-diphenyltetrazolium bromide (MTT) assay (Sigma-Aldrich in St. Louis, MO, USA). In summary, cells were seeded in 96-well culture plates and allowed to adhere overnight. The compounds under investigation were dissolved in DMSO and added to the wells, resulting in a final DMSO concentration of 1% (by volume). Different concentrations of the compounds were tested. After 72 h of incubation under standard culture conditions, 20 µL of MTT solution (4 µg mL^−1^ in PBS) was added to each well, and the plates were further incubated for 3 h. Subsequently, the medium was removed, and the formazan crystals were dissolved in DMSO. The absorbance of the resulting solution was measured at 540 nm using a microplate reader (Asys UVM 340 Microplate Reader, Biochrom, Cambridge, UK). Each experiment was independently performed three times in triplicate.

#### Statistical analysis

Statistical analysis was conducted using GraphPad Prism 9 software. Uniform significance levels were applied consistently across the entire manuscript as follows: ns (not significant) for *p* > 0.01, * for *p* < 0.01, ** for *p* < 0.001, *** for *p* < 0.0001, and **** for *p* < 0.00001. Statistical significance was determined by comparing the results to the DMSO-treated control using one- or two-way ANOVA.

### Acridine derivatives interactions with Calf thymus DNA

#### Chemicals and UV–Vis measurements preparation of stock solutions

The electronic spectra were recorded on an Evolution 300 spectrophotometer (Thermo Fischer Scientific, Waltham, MA, USA) in the range of 200–600 nm in the case of UV detection of intermolecular interactions with biomolecules together with a spectral band with a 2 nm width. *Calf Thymus* DNA (*CT*-DNA) was dissolved in Tris–HCl buffer (pH 7.43 ± 0.01) at 4 °C for 24 h with occasional stirring to ensure the formation of a homogeneous and stable biomolecule pre-solution. The final concentration of the stock deoxyribonucleic acid solution was determined spectrophotometrically at 260 nm by using a molar extinction coefficient of 6600 cm^−1^ M^−1^ for *CT*-DNA^44^. The concentration of freshly prepared *CT*-DNA dissolved in biological buffer (Tris–HCl 5 mM /50 mM NaCl; pH 7.43) was spectrophotometrically verified. Special attention was focused on the absorbances’ values ratio to be able to confirm the purity of the *CT*-DNA^45^. The *A*_260_*/A*_280_ ratio for *CT*-DNA was 1.84 which proves that the biomolecule studied treated as acridines’ cellular target was sufficiently free from proteins (Figs. S5, S8, and S11, SI). In the next step. the spectrophotometric titrations were performed at room temperature by gradually increasing the *CT*-DNA concentration added to individual acridines’ studied. After each addition of an amount of *CT*-DNA solution (470 μM), electronic absorption spectra of specific adducts formed were recorded by spectrophotometric titrations. Importantly, note that the dilution effects related to the measurement procedure used were strictly eliminated at the preliminary stage of the results processing.

#### Methodology used in binding studies 

The binding ability of six acridine derivatives to interact with *Calf thymus* DNA (*CT*-DNA, Sidma-Aldrich; catalog No. D8661-1ML) was investigated by spectrophotometric titrations. The UV absorbance at 260 nm of a diluted solution (1*/*20) of *CT*-DNA used in our experiments was measured to be 3.102 (path the length equal 1 cm) and the final concentration of the stock *CT*-DNA solution was calculated to be 0.47 mM *CT*-DNA in Tris–HCl. The initial concentrations of all acridine samples were prepared in Tris–HCl buffer [μM]: [**IKE1**] = 93; [**IKE8**] = 71; [**IKE3**] = 79; [**IKE7**] = 35; [**IKE9**] = 146; [**IKE14**] = 91. The time dependency of interaction for each object was different and was established based on the preliminary kinetic measurements (Figs. S6, S9, S12 SI). The *CT*-DNA solution then was added dropwise to the individual acridine solution. The stability of the binding properties of the compounds studied toward *CT*-DNA was examined by taking spectra after the time described in SI, and the same results were obtained for repetition made. Importantly, the preliminary step of results elaboration included the dilution effect elimination. Based upon the variation in absorbance, the association/binding constants of these interactions with *CT*-DNA were determined according to the Benesi–Hildebrand Eq. (1) presented below^46^:

1 $$\frac{{{\text{A}}}_{0}}{{\text{A}}-{{\text{A}}}_{0}}=\frac{{\upvarepsilon }_{{\text{G}}}}{{\upvarepsilon }_{{\text{H}}-{\text{G}}}-{\upvarepsilon }_{{\text{G}}}}+\frac{{\upvarepsilon }_{{\text{G}}}}{{\upvarepsilon }_{{\text{H}}-{\text{G}}}-{\upvarepsilon }_{{\text{G}}}}\cdot \frac{1}{{{\text{K}}}_{bind}[CT-\mathrm{DNA }]}$$

where A_0_ and A are the absorbances of the acridine derivative in the absence and presence of *CT*-DNA, respectively; ε_G_ and ε_H–G_ are the absorption coefficients of the acridine derivative unbounded and its adduct with *CT*-DNA, respectively. Thus, the double reciprocal plot of A_0_*/*(A–A_0_) versus 1/[DNA] is linear and the binding constant (*K*_*bind*_) was estimated based on the linear fit parameters established as the ratio of the intercept to the slope^47,48^.

#### Statistical analysis

All experiments were carried out in duplicates, in two independent experimental sets. The means ± SD were used in the statistical analysis of the data and the graphics.

### Supplementary Information

Supplementary Information.

## Data Availability

Data supporting the reported results are presented in this manuscript and its supplemental material. Raw data supporting our results are also available as data sets: Gabriel, I., Paluszkiewicz, E., Kozłowska-Tylingo, K., Roślik, M., & Rząd, K. (2021). Determination of the minimum inhibitory concentration of **C-1305** derivatives (**IKE1-IKE8**) against Candida strains [Data set]. Gdańsk University of Technology. https://doi.org/10.34808/47q7-jg74; Gabriel, I., Paluszkiewicz, E., Kozłowska-Tylingo, K., & Rząd, K. (2021). **IKE1-IKE3** (**C-1305** derivatives) inhibitory effect of the Yeast Topoisomerase II relaxation activity (Version 2.0) [Data set]. Gdańsk University of Technology. https://doi.org/10.34808/j3t1-qg11; Rząd, K., Panuś, O., Gabriel, I., Paluszkiewicz, E., & Kozłowska-Tylingo, K. (2023). Determination of the minimum inhibitory concentration of **C-1330** derivatives (**IKE9-IKE14**) against *Candida* strains [Data set]. Gdańsk University of Technology. https://doi.org/10.34808/wx9v-ck79;
