# Supplementary material for: Targeting yeast topoisomerase II by imidazo and triazoloacridinone derivatives resulting in their antifungal activity
Source: Sci Rep. 2024 Feb 13;14:3594. doi: 10.1038/s41598-024-54252-0 (PMC10864382; doi:10.1038/s41598-024-54252-0)
Supplement: Supplementary file 1 — Supplementary Information. [file 41598_2024_54252_MOESM1_ESM.pdf]

Supplementary data

Targeting yeast topoisomerase II by imidazo and triazoloacridinone derivatives resulting in their antifungal activity.

Kamila Rząd[a]*, Iwona Gabriel[a], Ewa Paluszkiewicz[a], Aleksandra Kuplińska[a], Mateusz Olszewski[a], Agnieszka Chylewska[b], Aleksandra M. Dąbrowska[b], Katarzyna Kozłowska-Tylingo[a]

[a]*Department of Pharmaceutical Technology and Biochemistry, Faculty of Chemistry and BioTechMed Center, Gdansk University of Technology, 11/12 Narutowicza Str., 80-233 Gdansk, Poland*

[b]*Department of Bioinorganic Chemistry, Faculty of Chemistry, University of Gdansk, Wita Stwosza 63, 80-308 Gdansk, Poland*

Table of contents:

1. **Figure S1 ……………………………………………………………………………………2**
2. **Figure S2………………………………………………………………………………………3**
3. **^{1}H NMR, ^{13}C NMR, ESI-MS and HPLC Spectra for target compounds…………………4-54**
4. **Figure S3……………………………………………………………………………………55**
5. **Figure S4……………………………………………………………………………………56**
6. **Figure S5……………………………………………………………………………………57**
7. **Figure S6……………………………………………………………………………………58**
8. **Figure S7……………………………………………………………………………………59**
9. **Figure S8……………………………………………………………………………………60**
10. **Figure S9……………………………………………………………………………………61**
11. **Figure S10…………………………………………………………………………………62**
12. **Figure S11…………………………………………………………………………………63**
13. **Figure S12…………………………………………………………………………………64**
14. **Figure S13…………………………………………………………………………………65**
15. **Table S1 ……………………………………………………………………………………66**
16. **Figure S14…………………………………………………………………………………67**
17. **Figure S15…………………………………………………………………………………68**
18. **Figure S16…………………………………………………………………………………69**
19. **Figure S17…………………………………………………………………………………70**

* Corresponding author. Tel: +48 583472193; Fax: +48 583471144; E-mail: kamila.rzad@pg.edu.pl

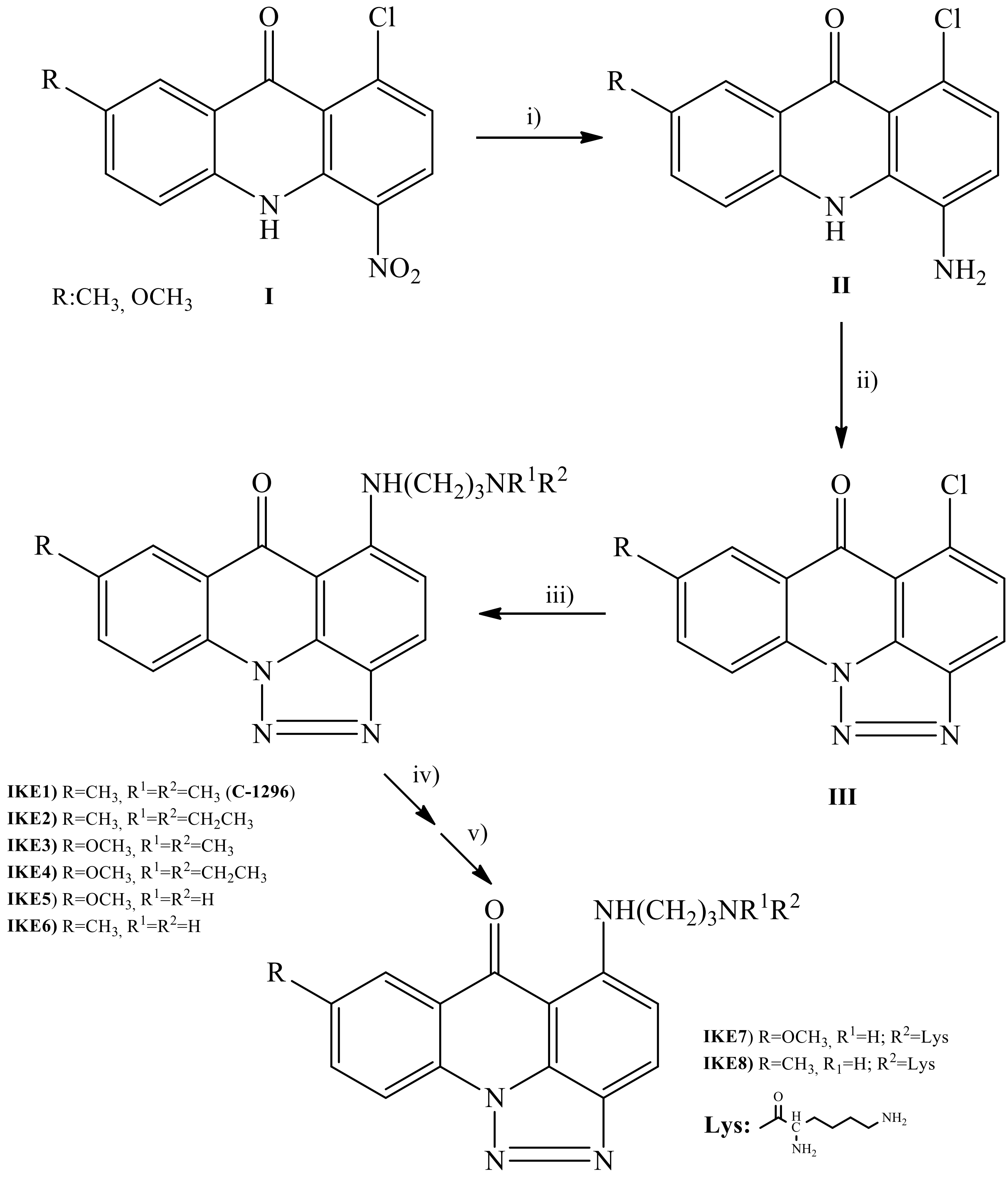

Figure S1. Strategy of the synthesis of IKE1- IKE8. Reagent and conditions: i) concd HCl, EtOH, $SnCl_2$, reflux 6h; ii) $NaNO_2$, concd HCl, 4H, rt; iii) DMA, The corresponding amine, 60°C, 3h; iv) N-BOC-Lys(Z)-OH, NHS, DCC, TEA, DMSO,24h, rt; v) 33% HBr, AcOH

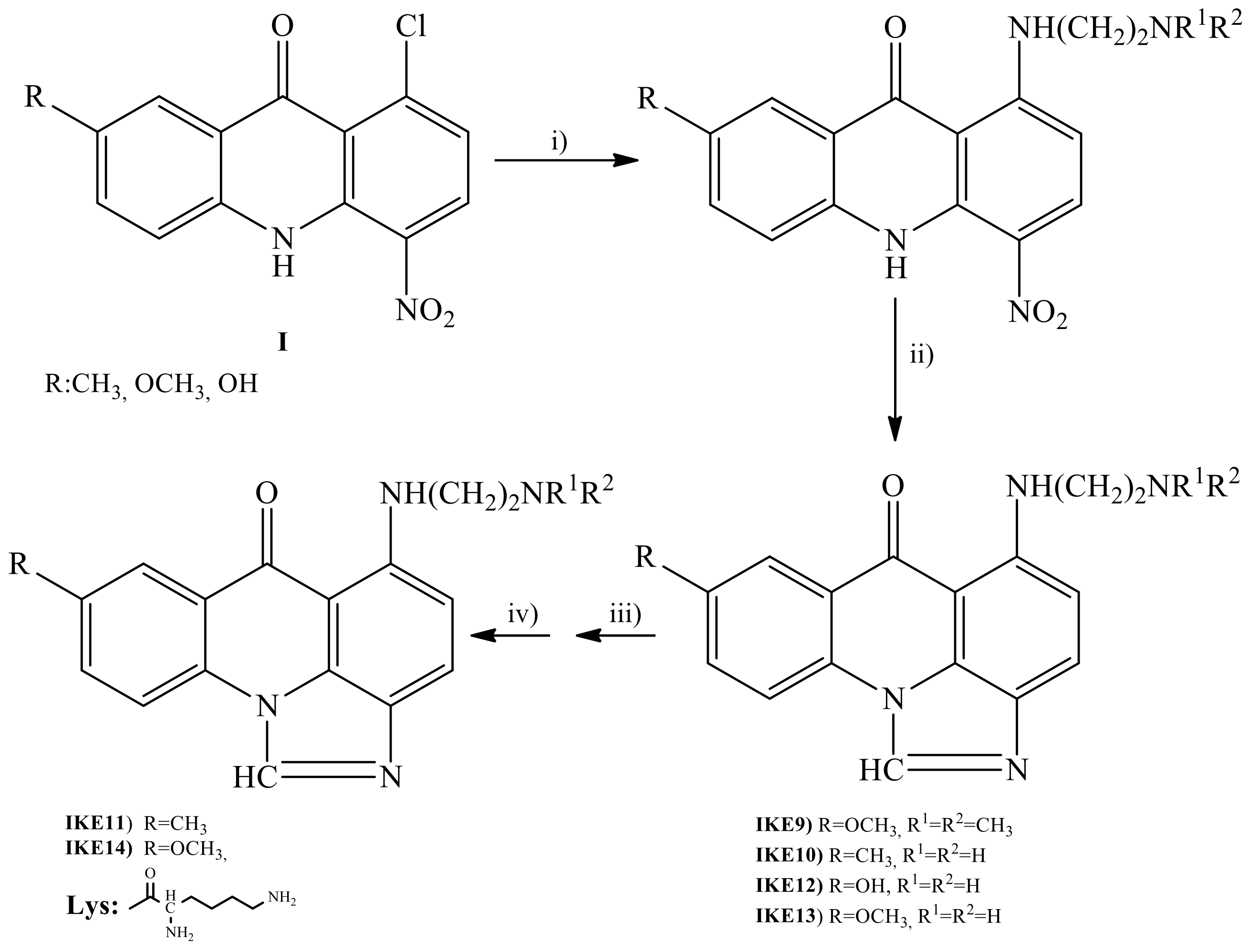

Figure S2. Strategy of the synthesis of IKE9- IKE14. Reagent and conditions: i) The corresponding amine; DMSO, 2.5h, rt; ii) 10% Pd/C, HCOOH, 24H, rt; iii) N-BOC-Lys(Z)-OH, NHS, DCC, TEA, DMSO,24h, rt; iv) 33% HBr, AcOH

<u>IKE1</u> **¹H NMR Spectra**

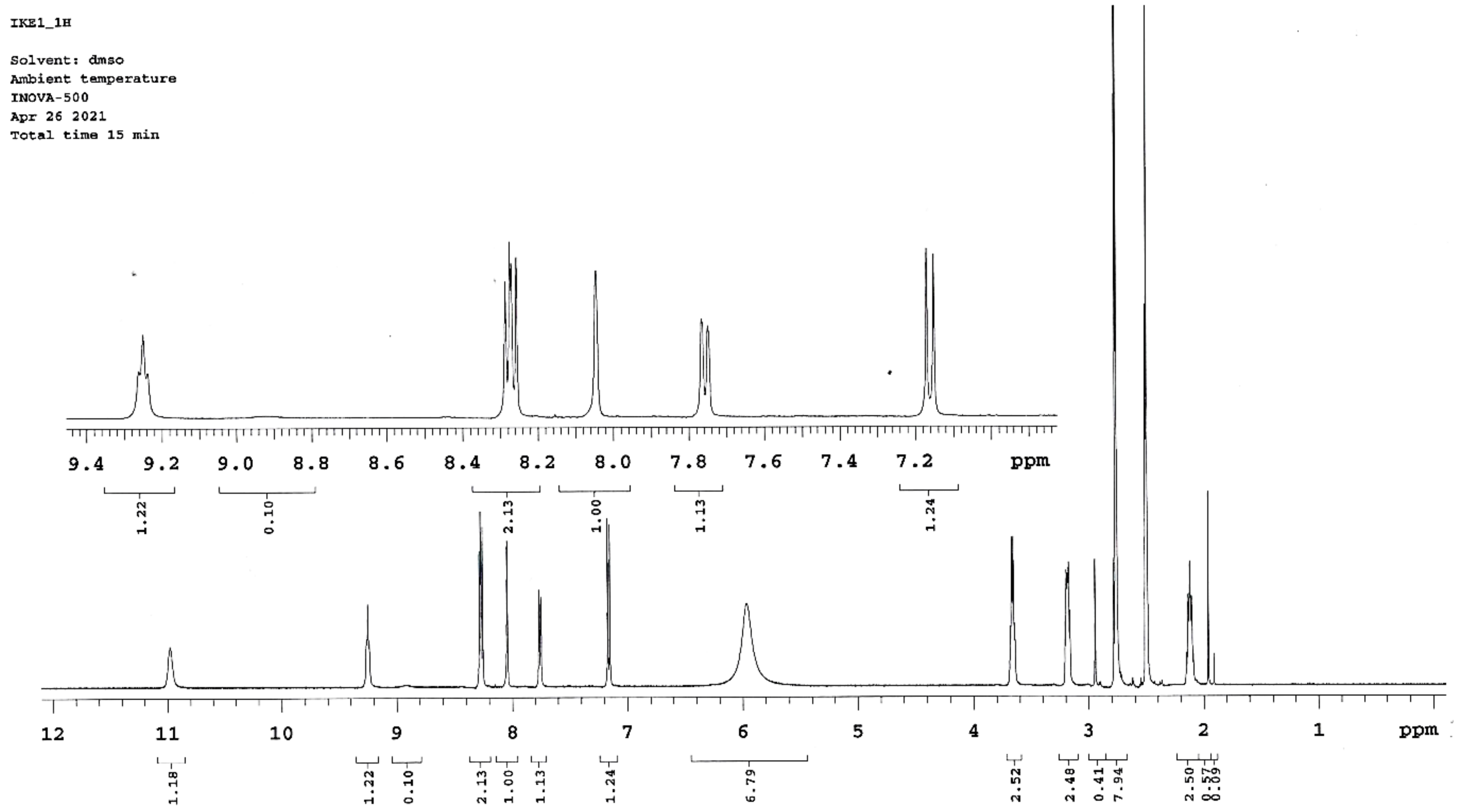

<u>IKE1</u> ^{13}C NMR Spectra

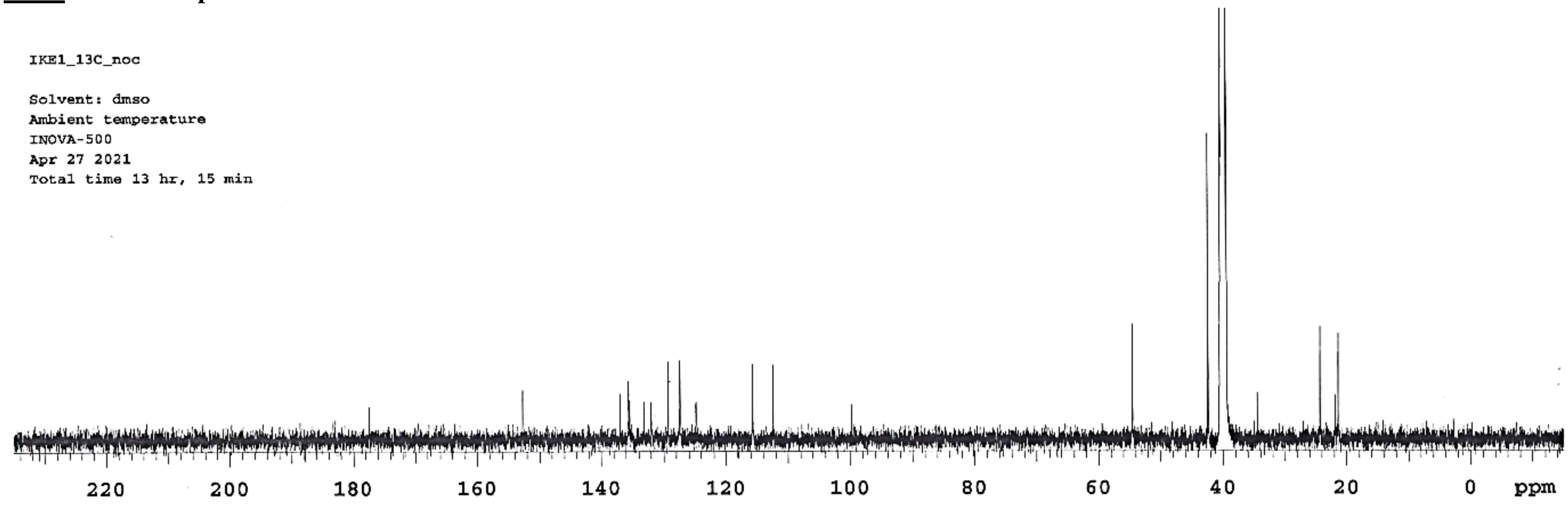

<u>IKE1</u> ESI-MS Spectra

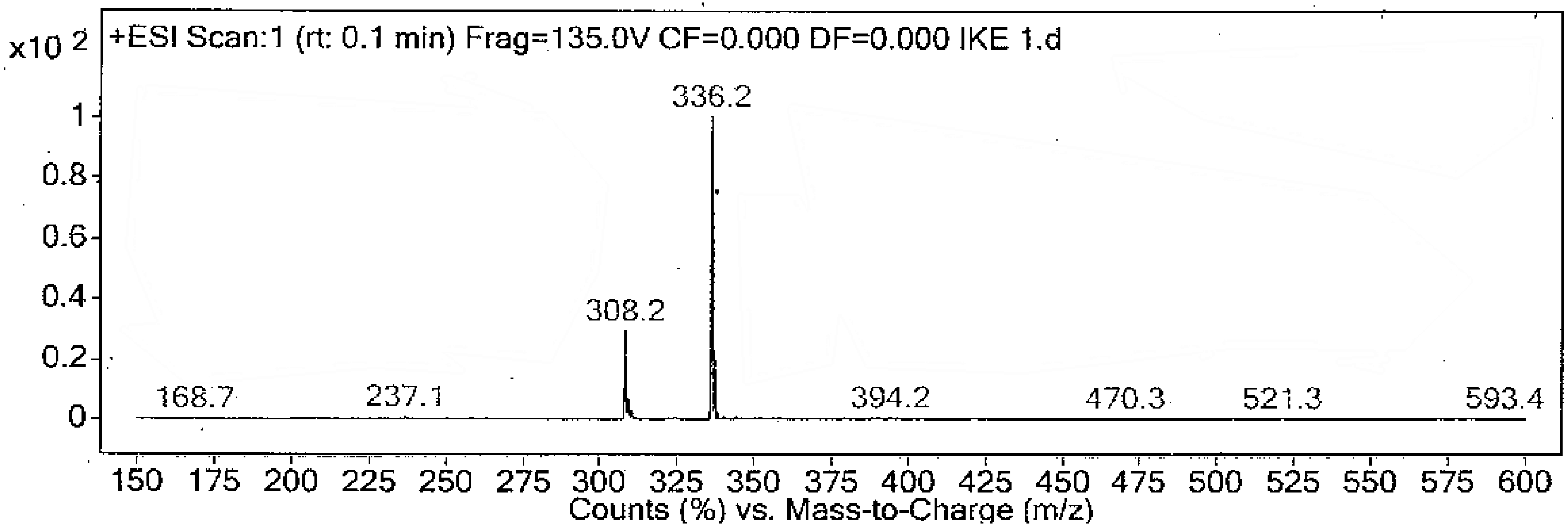

IKE1 **HPLC Spectra**

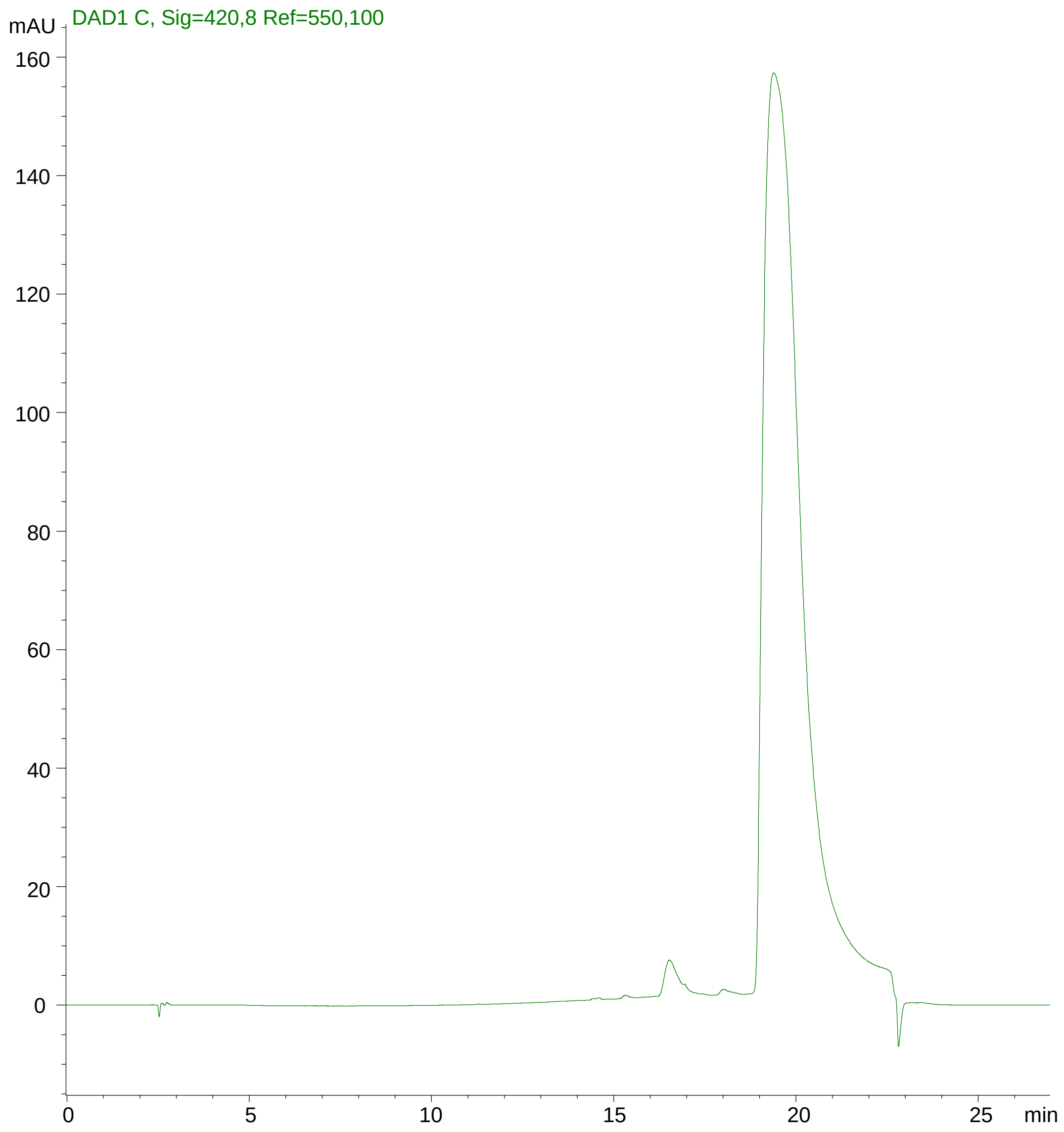

IKE2 ^{1}H NMR Spectra

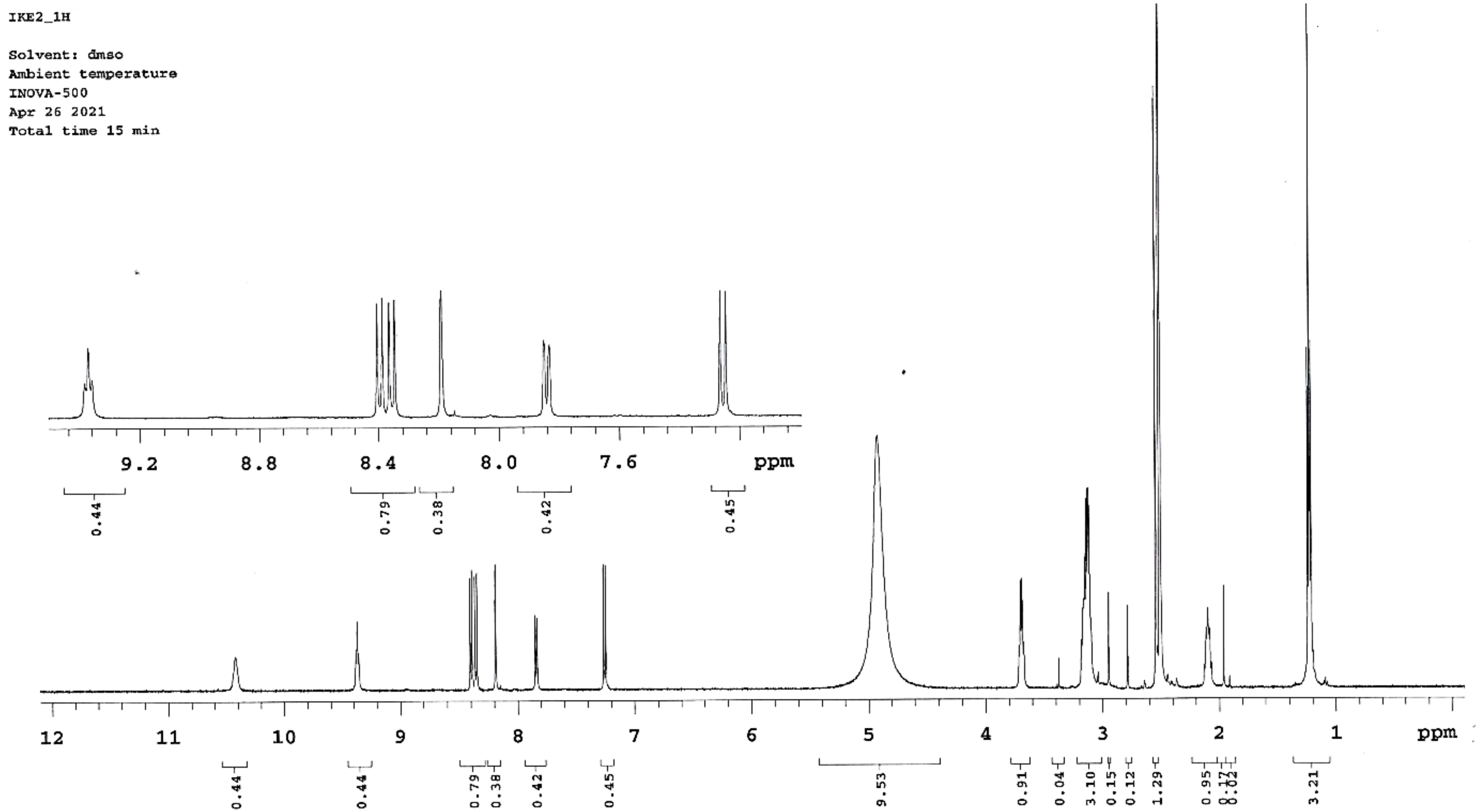

IKE2 ^{13}C **NMR Spectra**

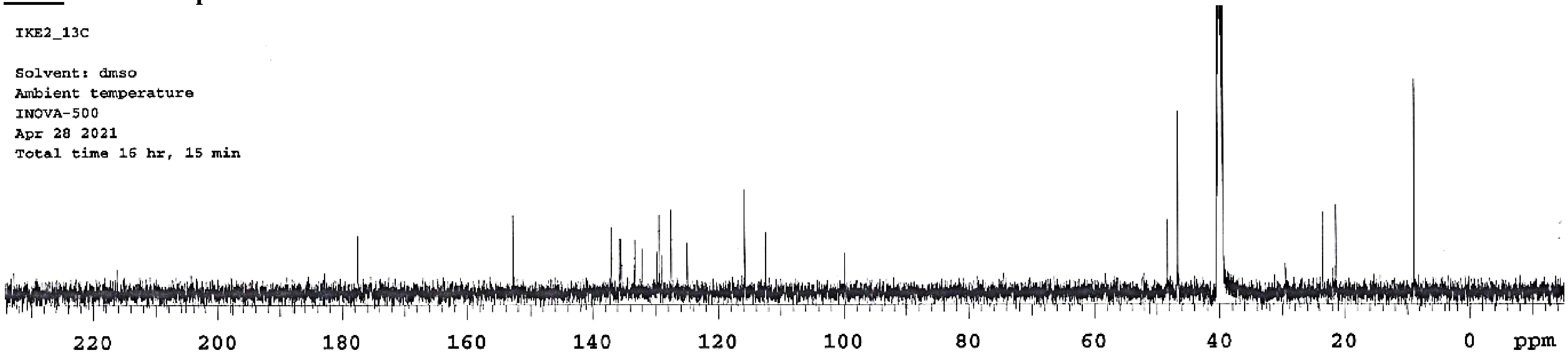

IKE2 ESI-MS Spectra

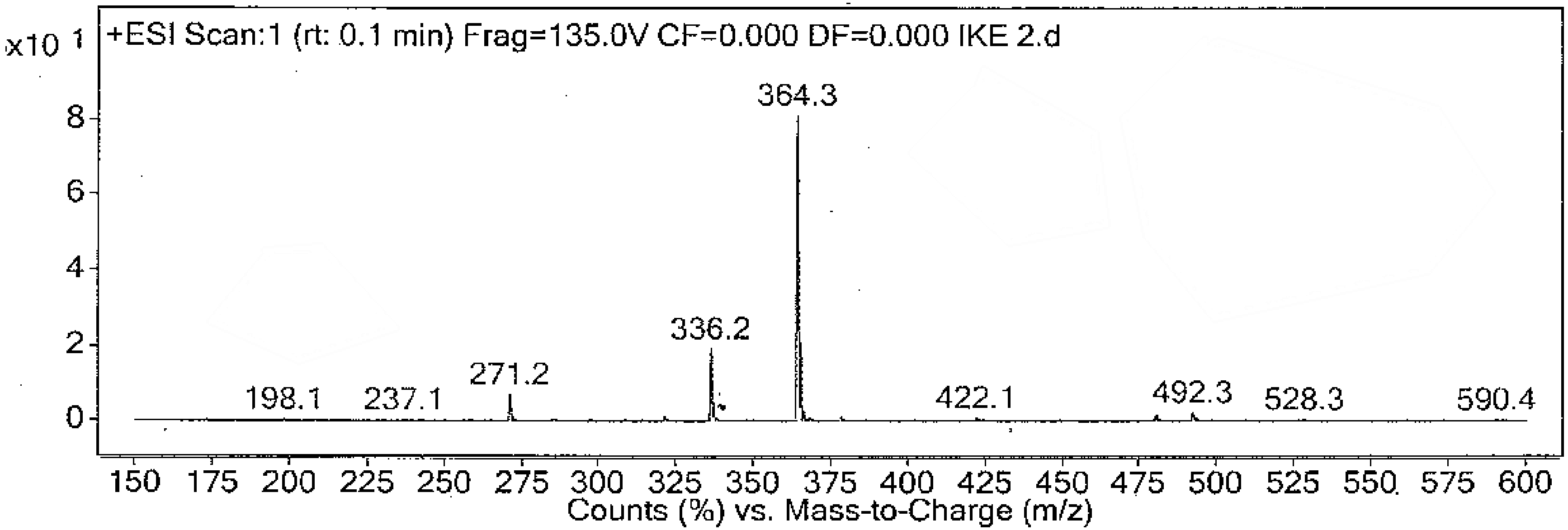

<u>IKE2</u> HPLC Spectra

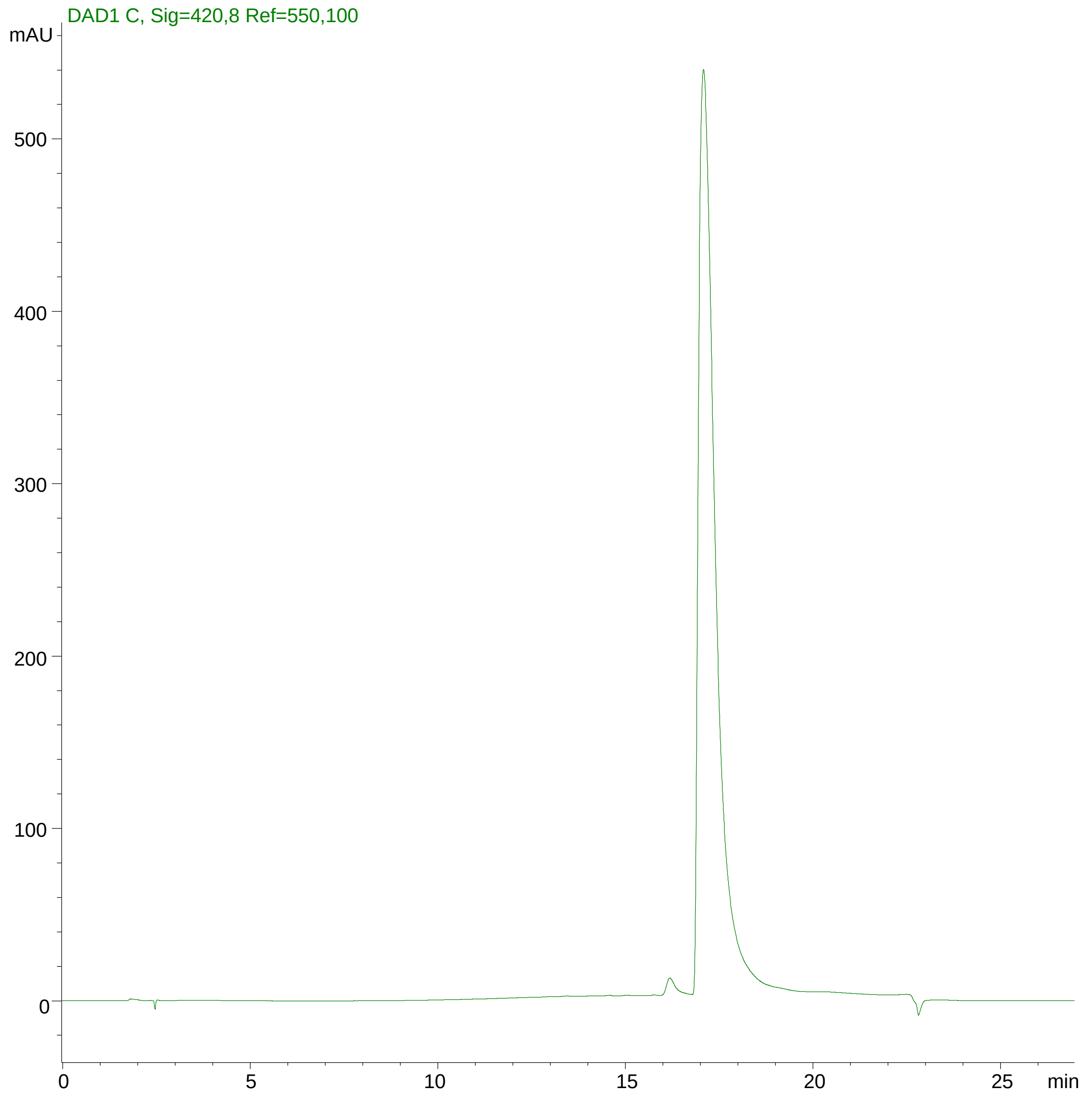

IKE3 ^{1}H NMR Spectra

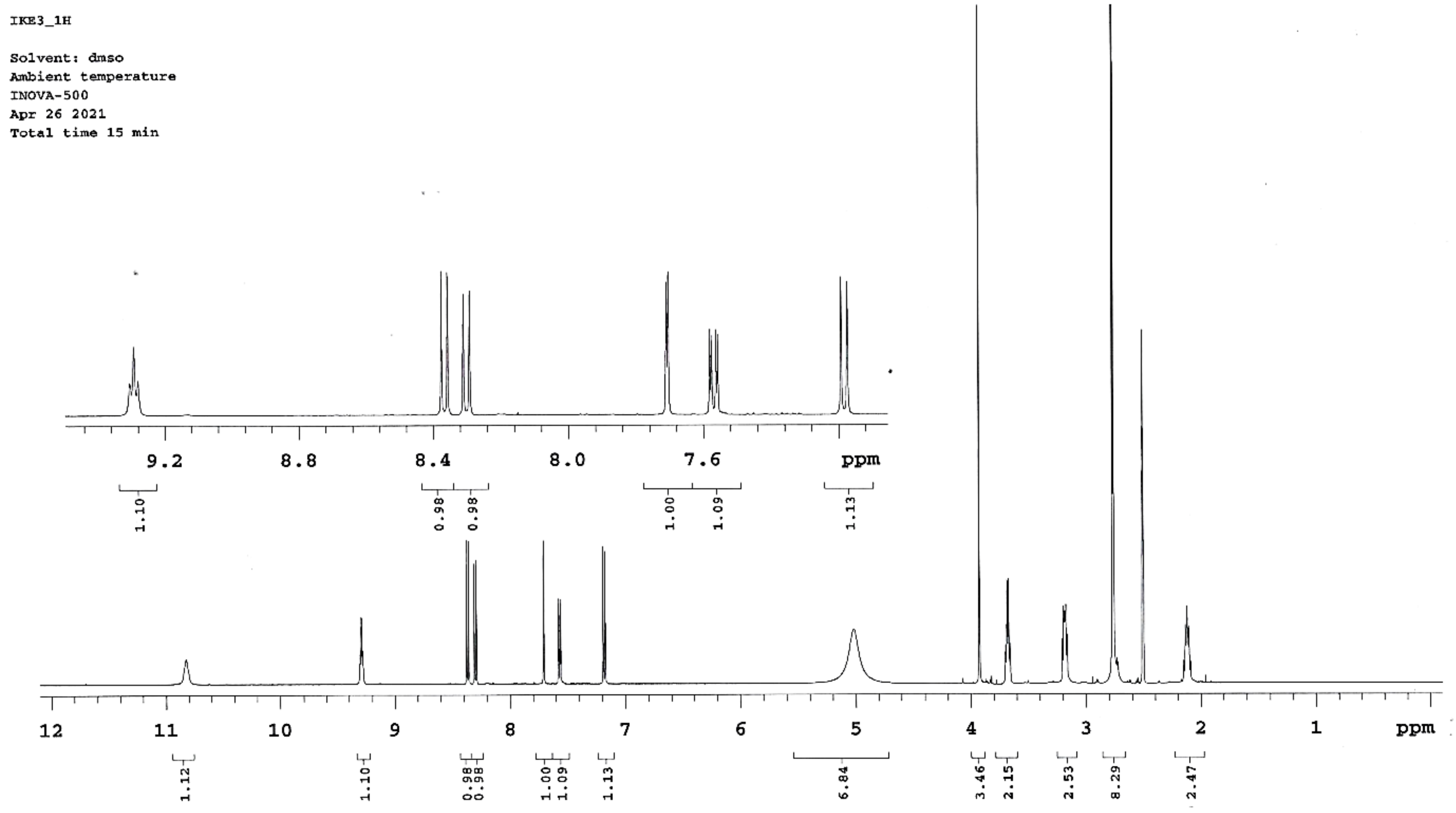

<u>IKE3</u> ^{13}C NMR Spectra

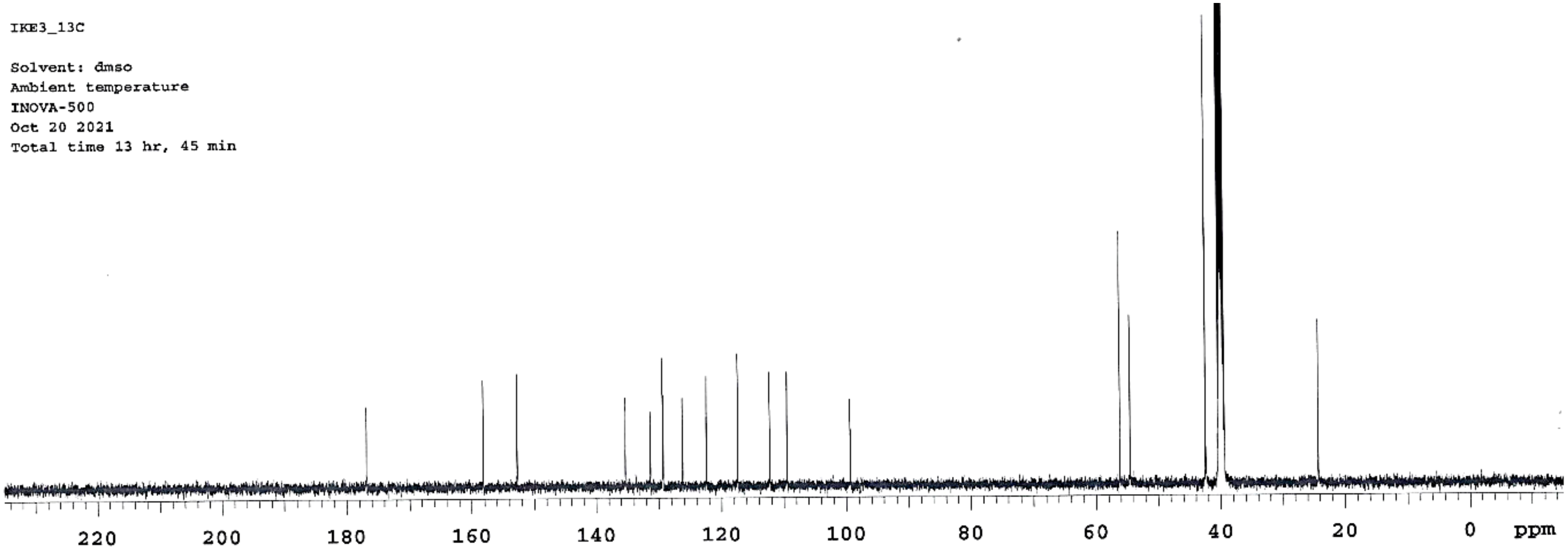

<u>IKE3</u> ESI-MS Spectra

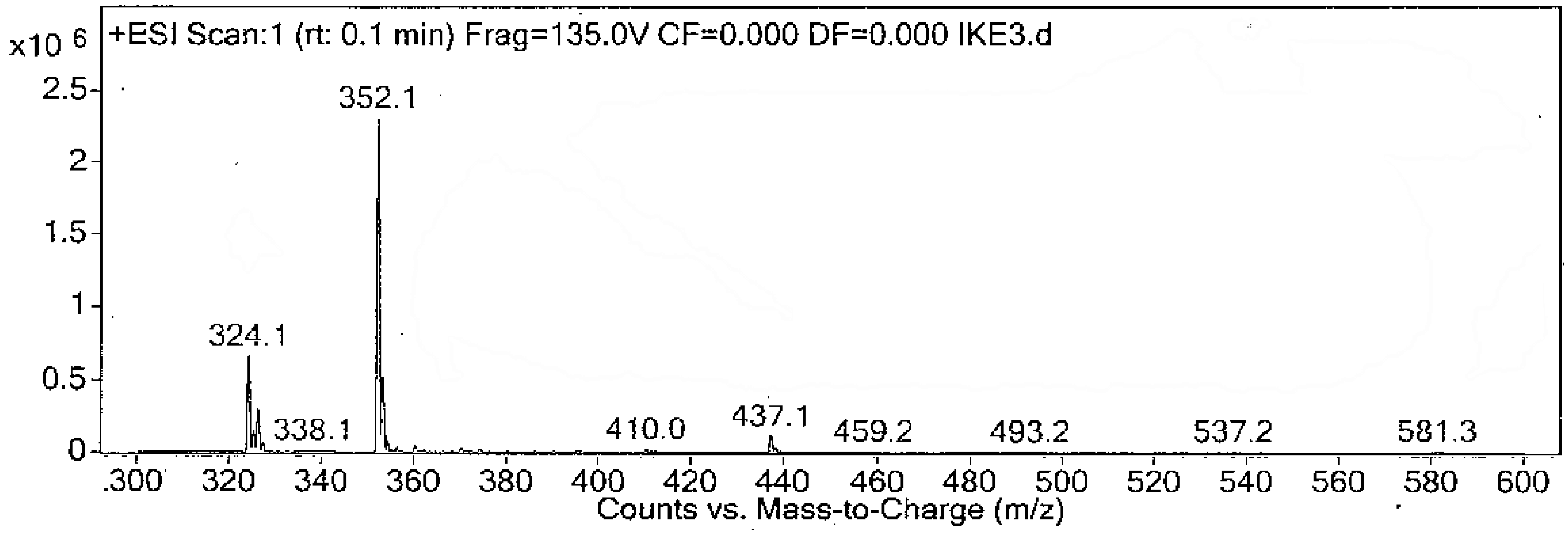

<u>IKE3</u> HPLC Spectra

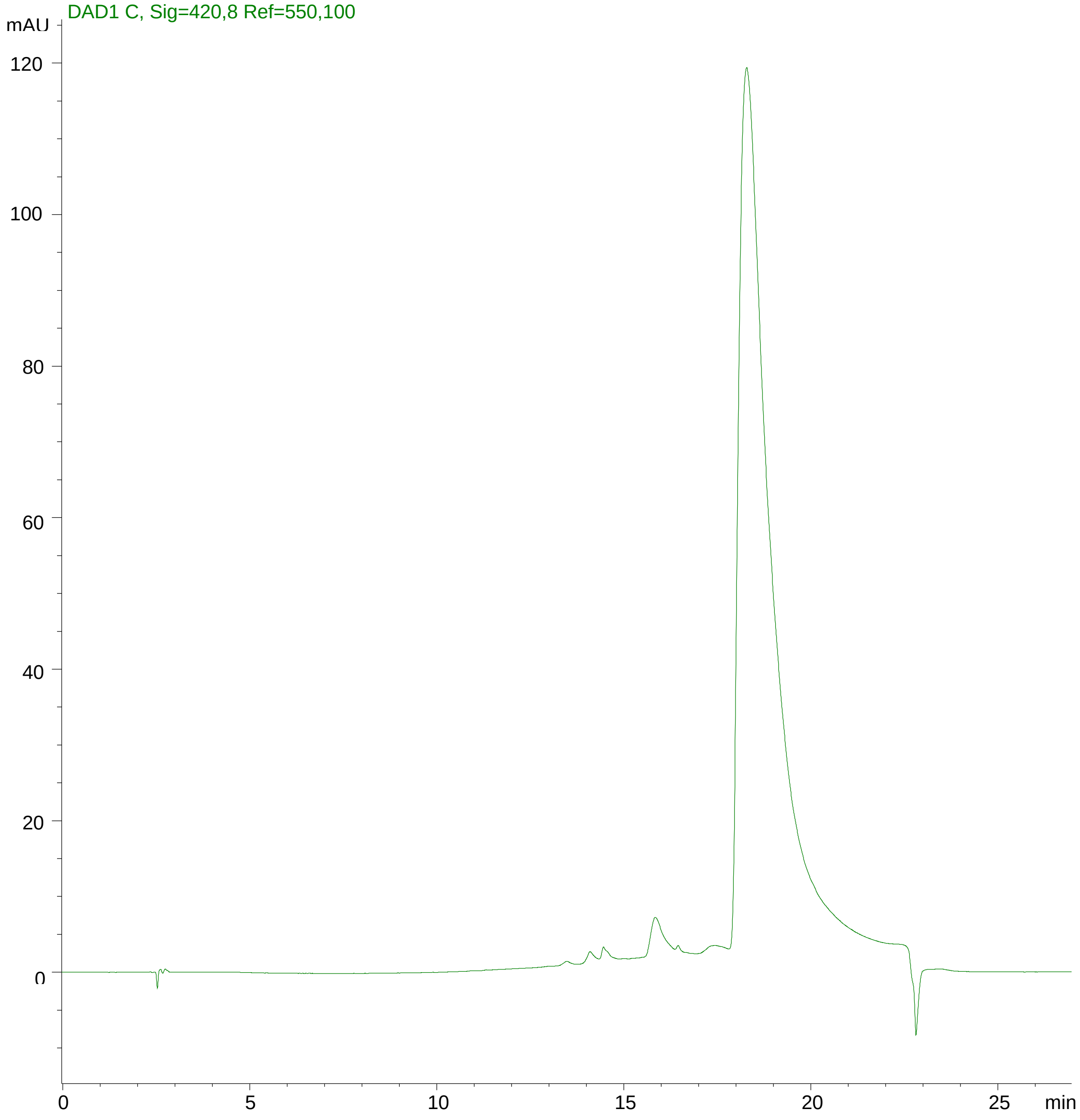

IKE4 ^{1}H NMR Spectra

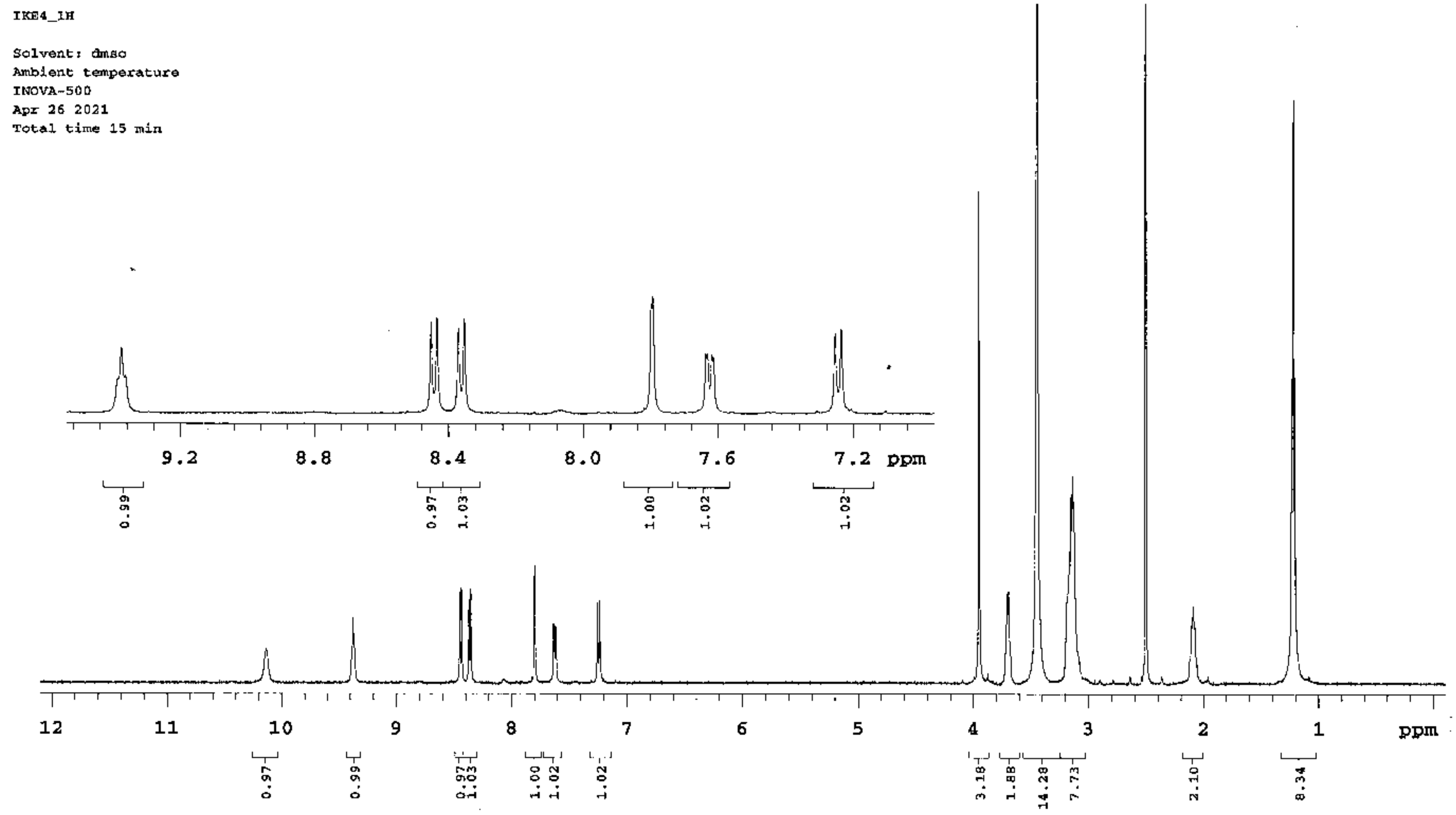

IKE4 ^{13}**C NMR Spectra**

IKE4_13C

Solvent: d2o
Ambient temperature
File: IKE4_13C
INOVA-500
May 19 2021
Total time 17 hr, 45 min

INDEX	FREQUENCY	PPM	HEIGHT	INDEX	FREQUENCY	PPM	HEIGHT
1	27025.2	215.048	4.7	21	2064.6	16.429	4.9
2	25071.0	199.497	-4.5	22	1977.3	15.734	-4.6
3	23211.6	184.702	6.8	23	1121.0	8.920	-4.6
4	21869.1	174.019	5.5	24	1032.8	8.218	400.0
5	19648.3	156.347	6.7	25	-281.9	-2.243	5.1
6	19016.4	151.319	6.3				
7	16686.2	132.777	7.4				
8	16076.3	127.924	7.9				
9	15843.3	126.070	7.2				
10	15466.4	123.071	6.4				
11	15237.3	121.247	5.7				
12	14497.9	115.364	5.8				
13	14045.3	111.763	6.2				
14	13392.3	106.567	6.3				
15	12079.5	96.121	6.8				
16	6911.9	55.000	45.0				
17	6142.9	48.881	108.2				
18	5952.0	47.362	289.6				
19	4970.1	39.549	37.7				
20	2892.1	23.014	85.9				

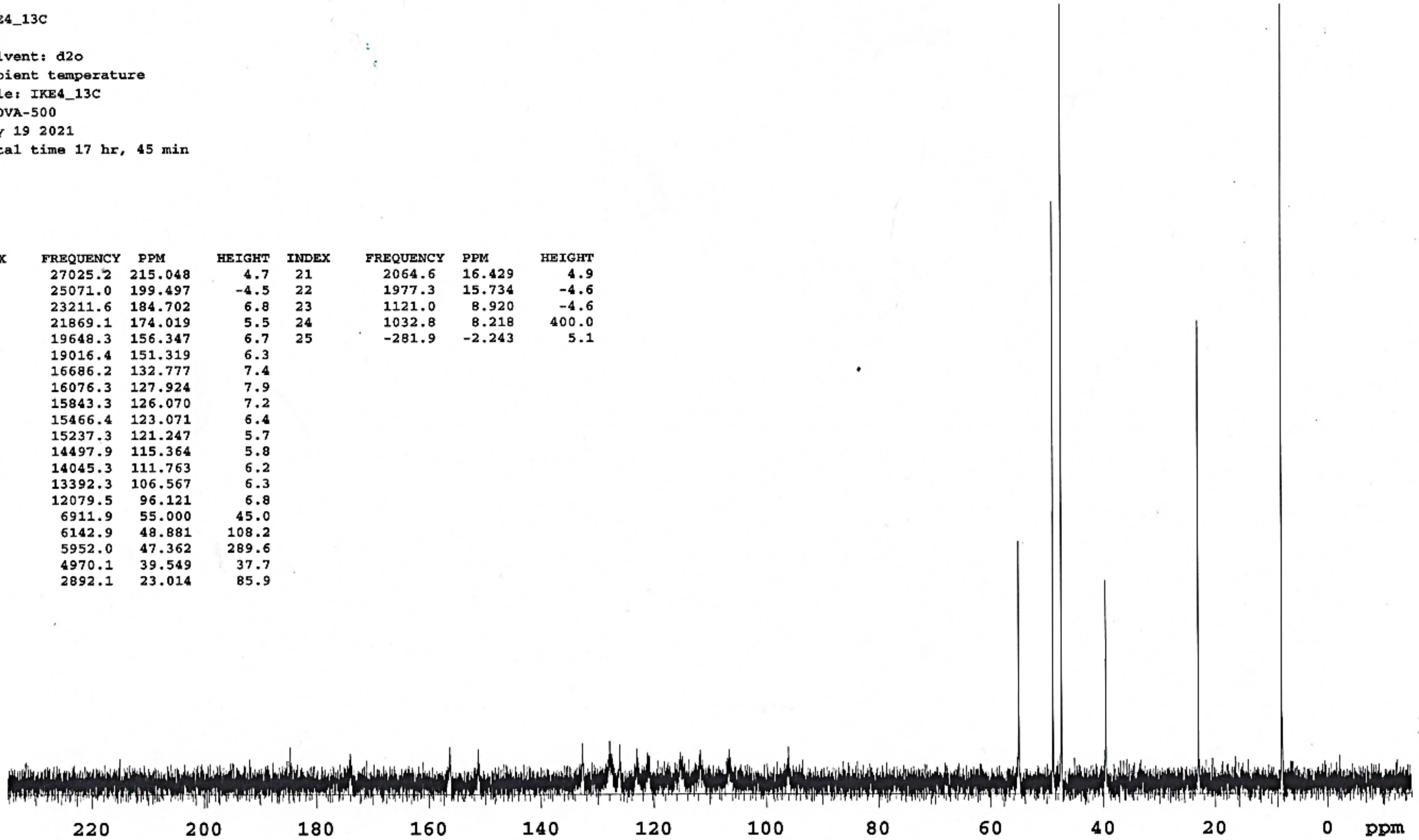

IKE4 ESI-MS Spectra

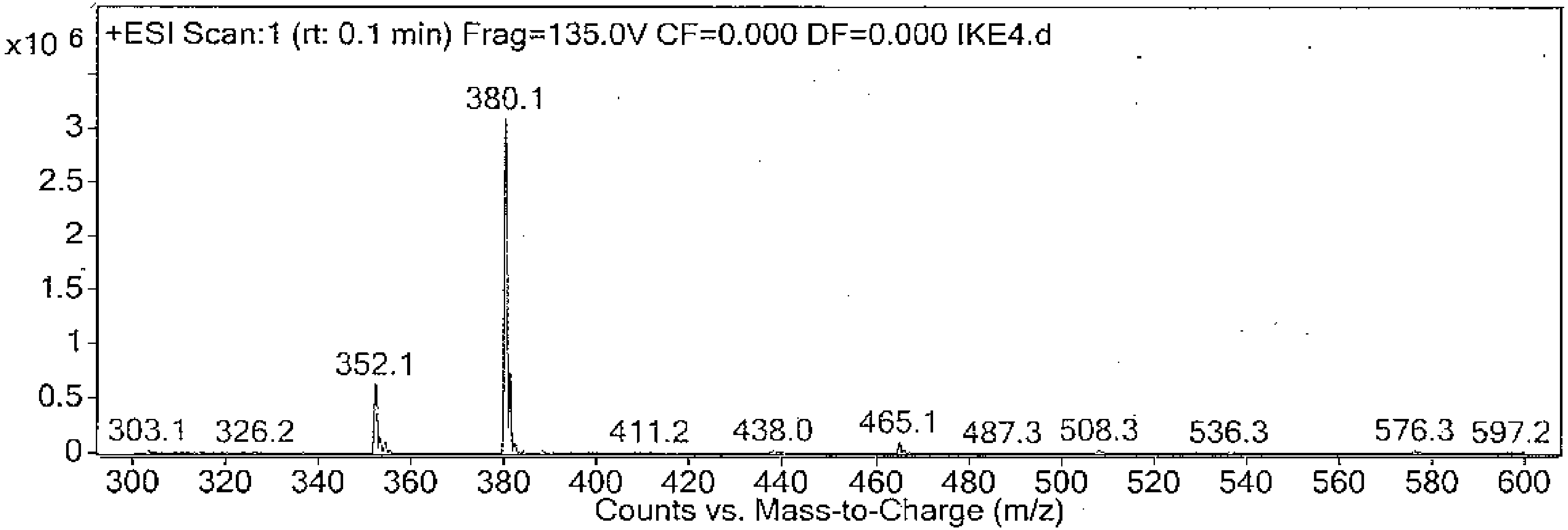

<u>IKE4</u> HPLC Spectra

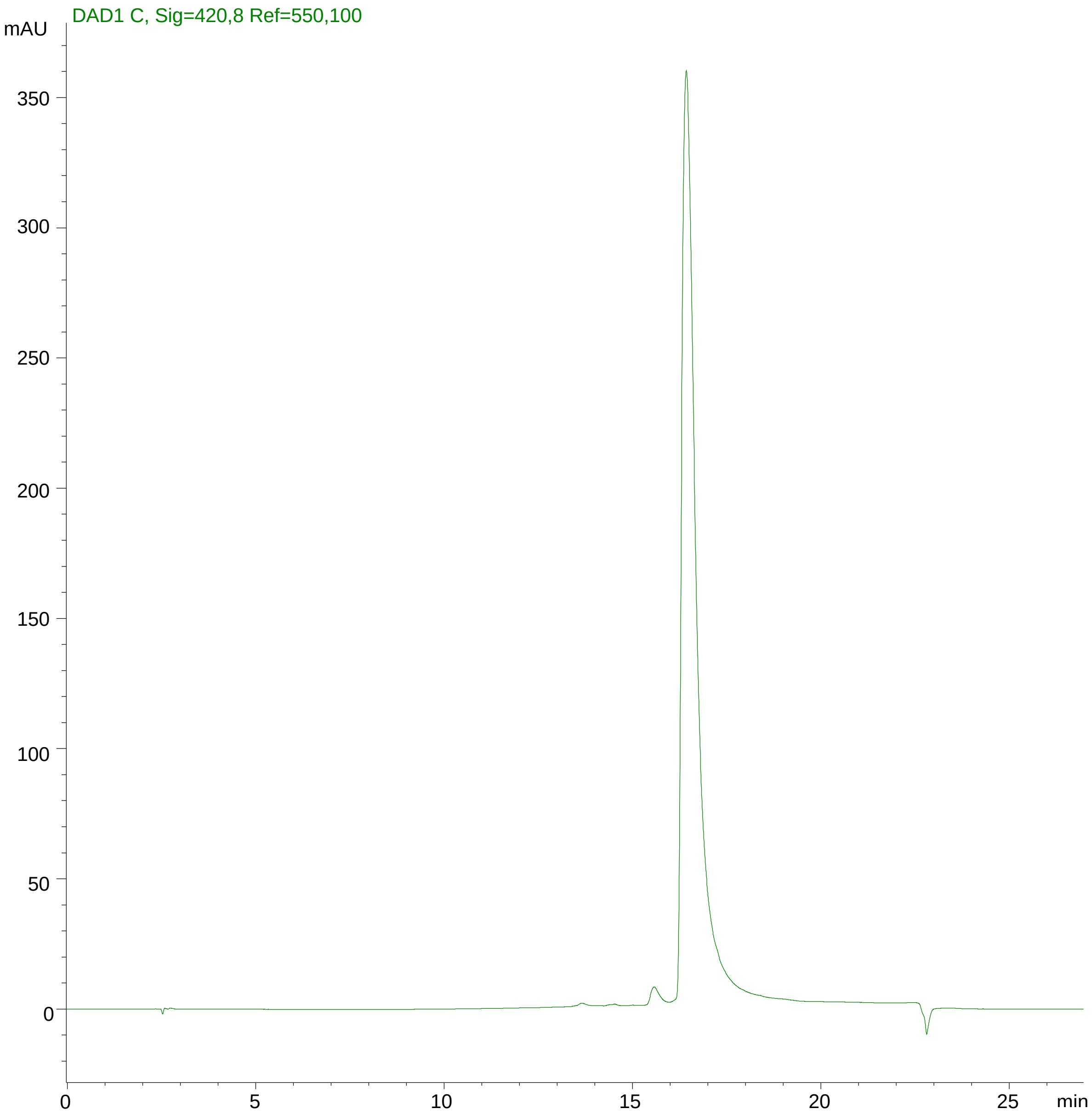

<u>IKE5</u> **^{1}H NMR Spectra**

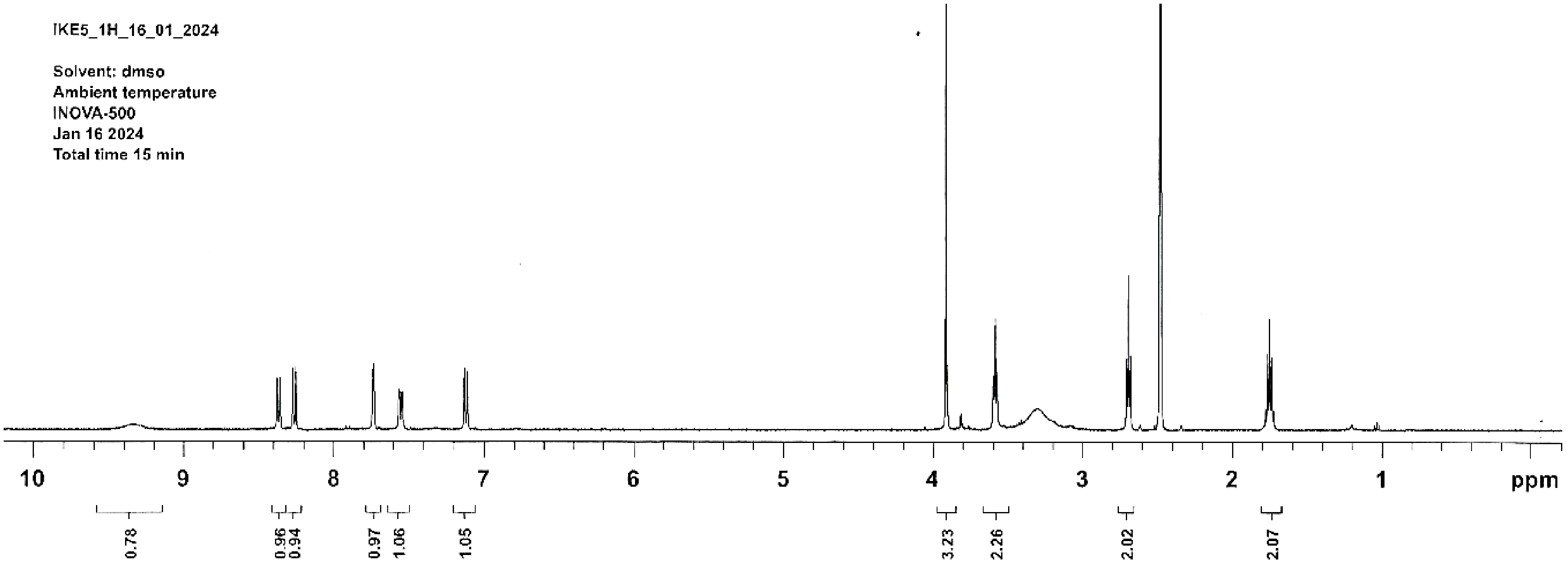

<u>IKE5</u> ^{13}C NMR Spectra

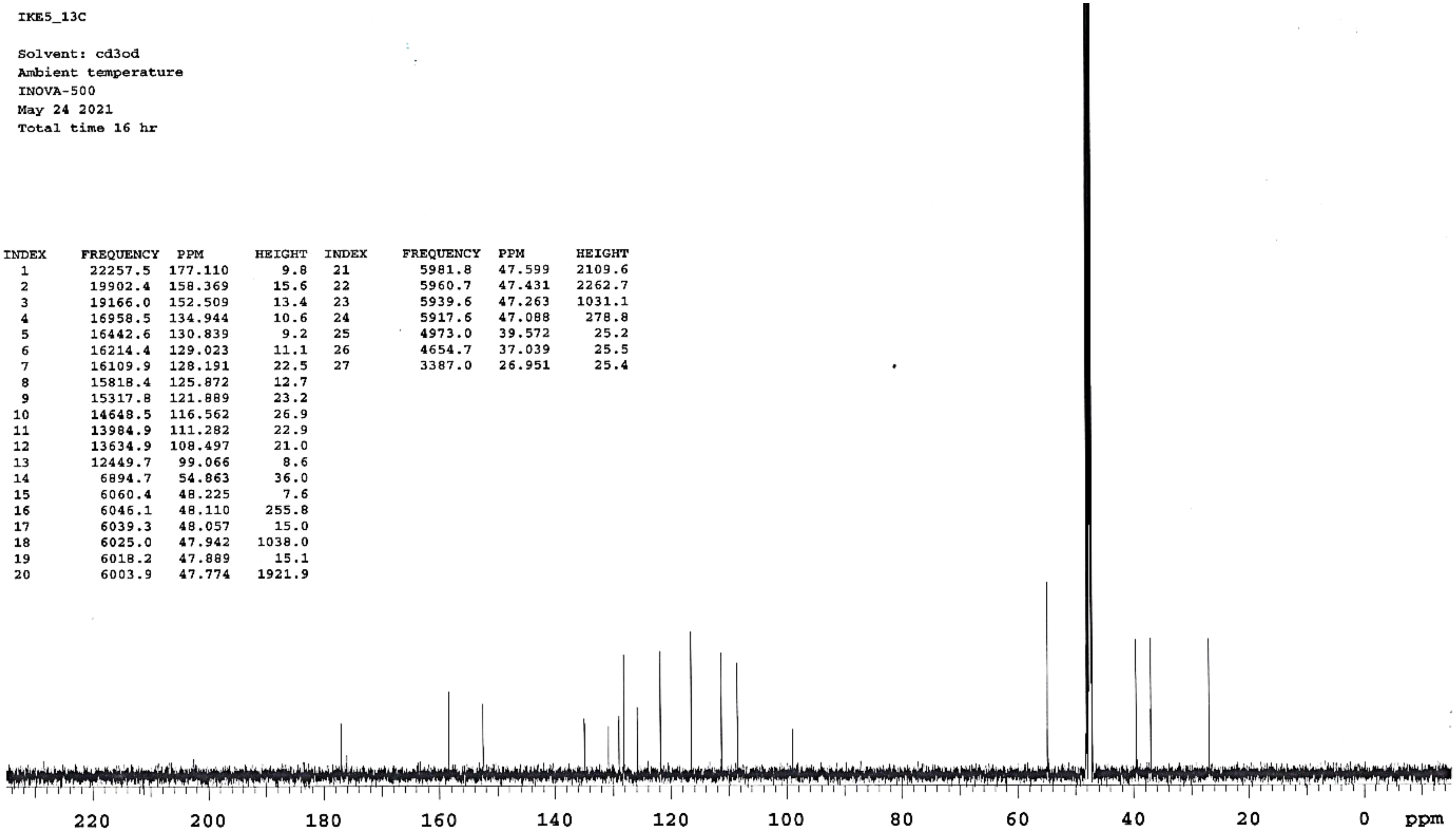

INDEX	FREQUENCY	PPM	HEIGHT	INDEX	FREQUENCY	PPM	HEIGHT
1	22257.5	177.110	9.8	21	5981.8	47.599	2109.6
2	19902.4	158.369	15.6	22	5960.7	47.431	2262.7
3	19166.0	152.509	13.4	23	5939.6	47.263	1031.1
4	16958.5	134.944	10.6	24	5917.6	47.088	278.8
5	16442.6	130.839	9.2	25	4973.0	39.572	25.2
6	16214.4	129.023	11.1	26	4654.7	37.039	25.5
7	16109.9	128.191	22.5	27	3387.0	26.951	25.4
8	15818.4	125.872	12.7				
9	15317.8	121.889	23.2				
10	14648.5	116.562	26.9				
11	13984.9	111.282	22.9				
12	13634.9	108.497	21.0				
13	12449.7	99.066	8.6				
14	6894.7	54.863	36.0				
15	6060.4	48.225	7.6				
16	6046.1	48.110	255.8				
17	6039.3	48.057	15.0				
18	6025.0	47.942	1038.0				
19	6018.2	47.889	15.1				
20	6003.9	47.774	1921.9				

<u>IKE5</u> ESI-MS Spectra

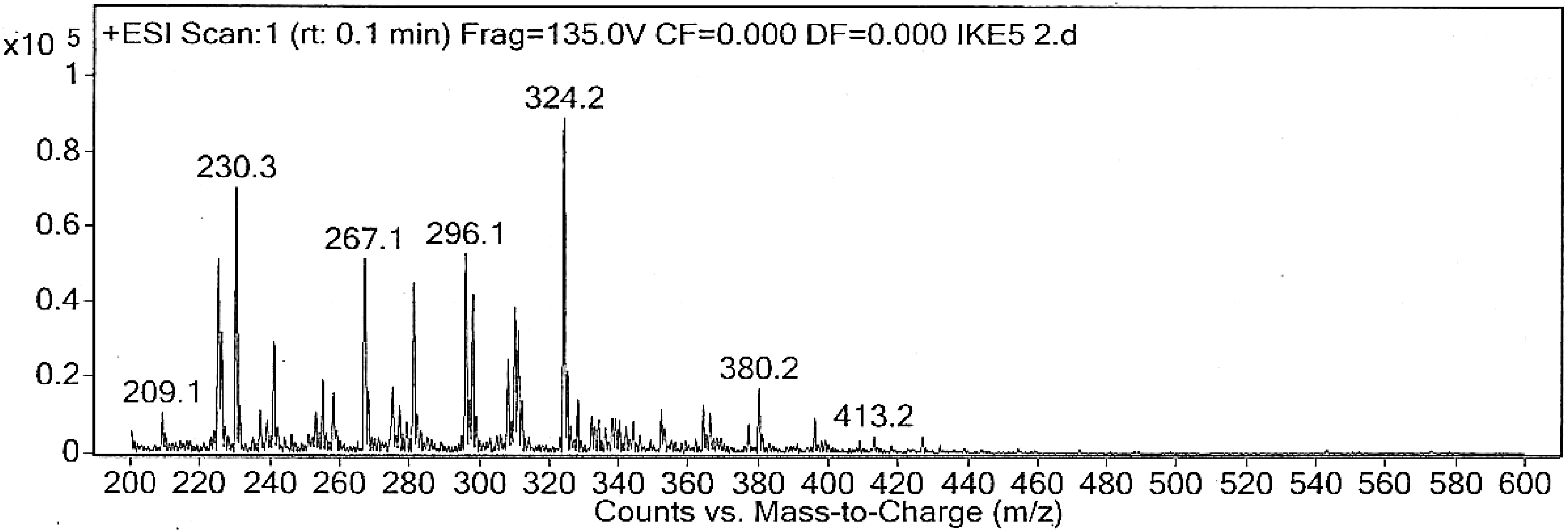

<u>IKE5</u> HPLC Spectra

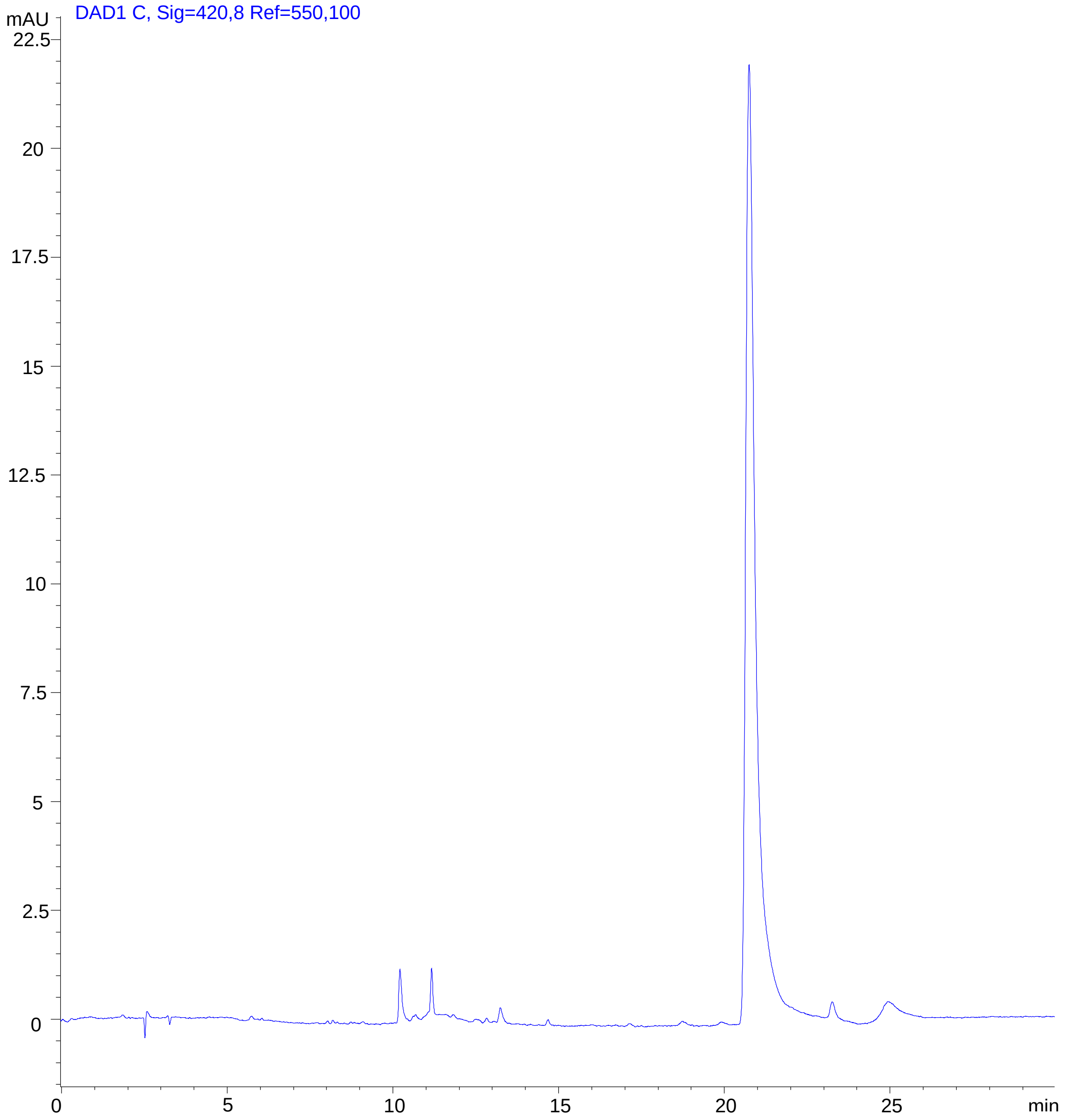

IKE6 ^{1}H NMR Spectra

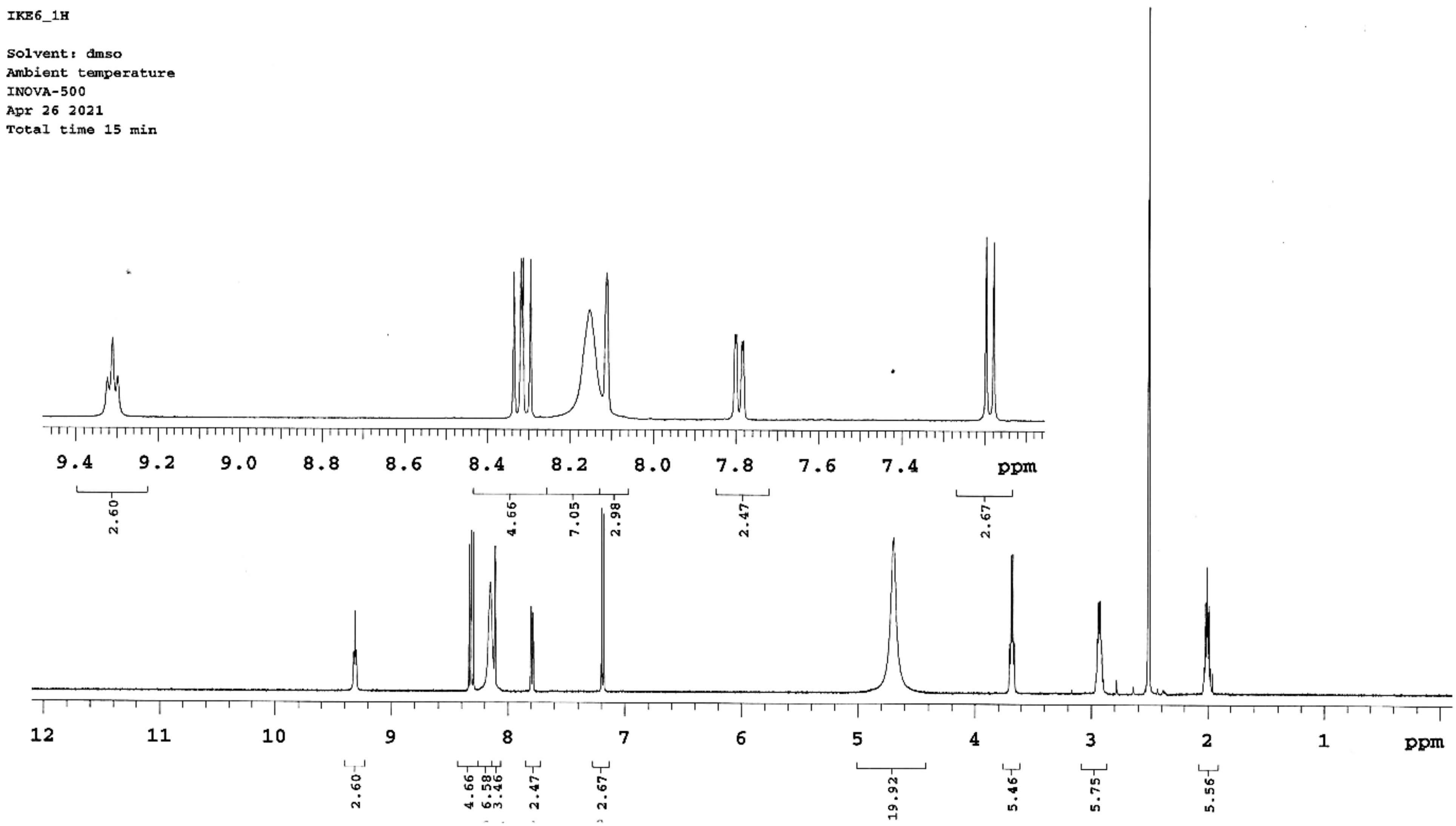

IKE6 ^{13}C NMR Spectra

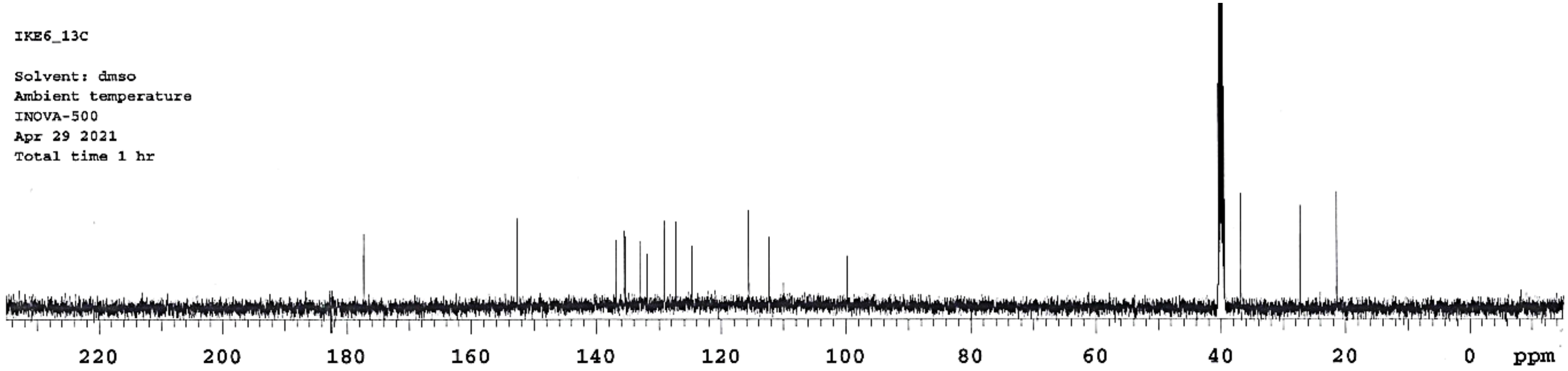

IKE6 ESI-MS Spectra

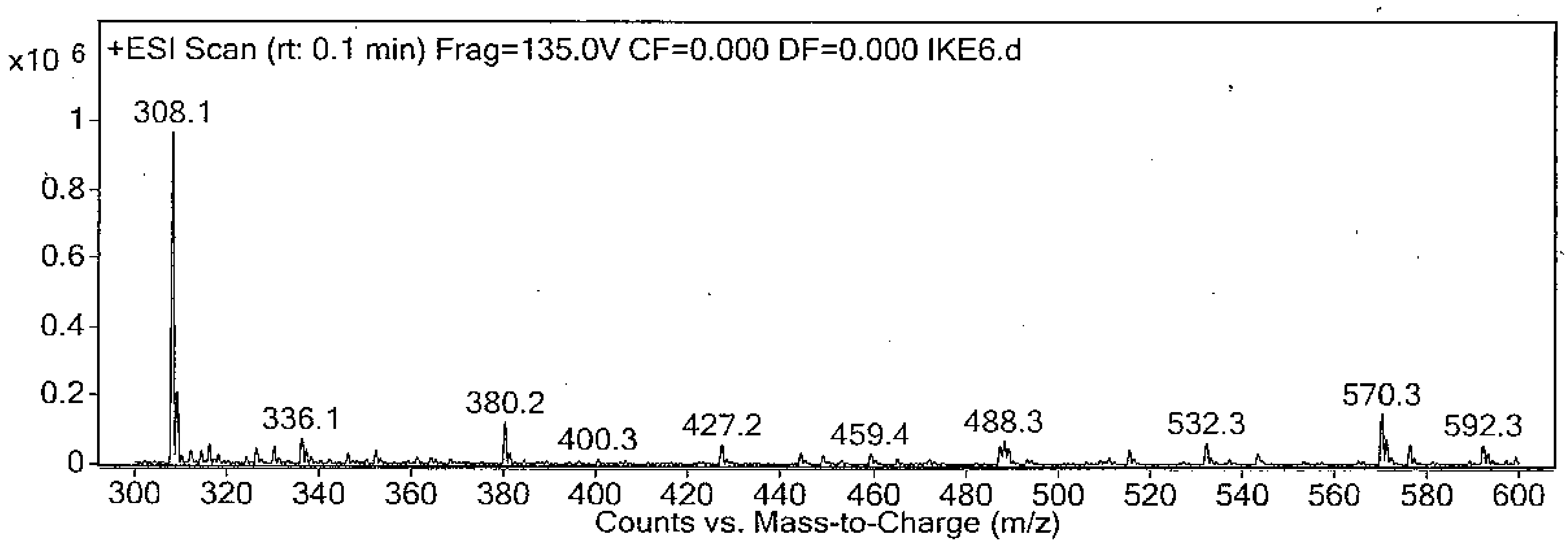

<u>IKE6</u> HPLC Spectra

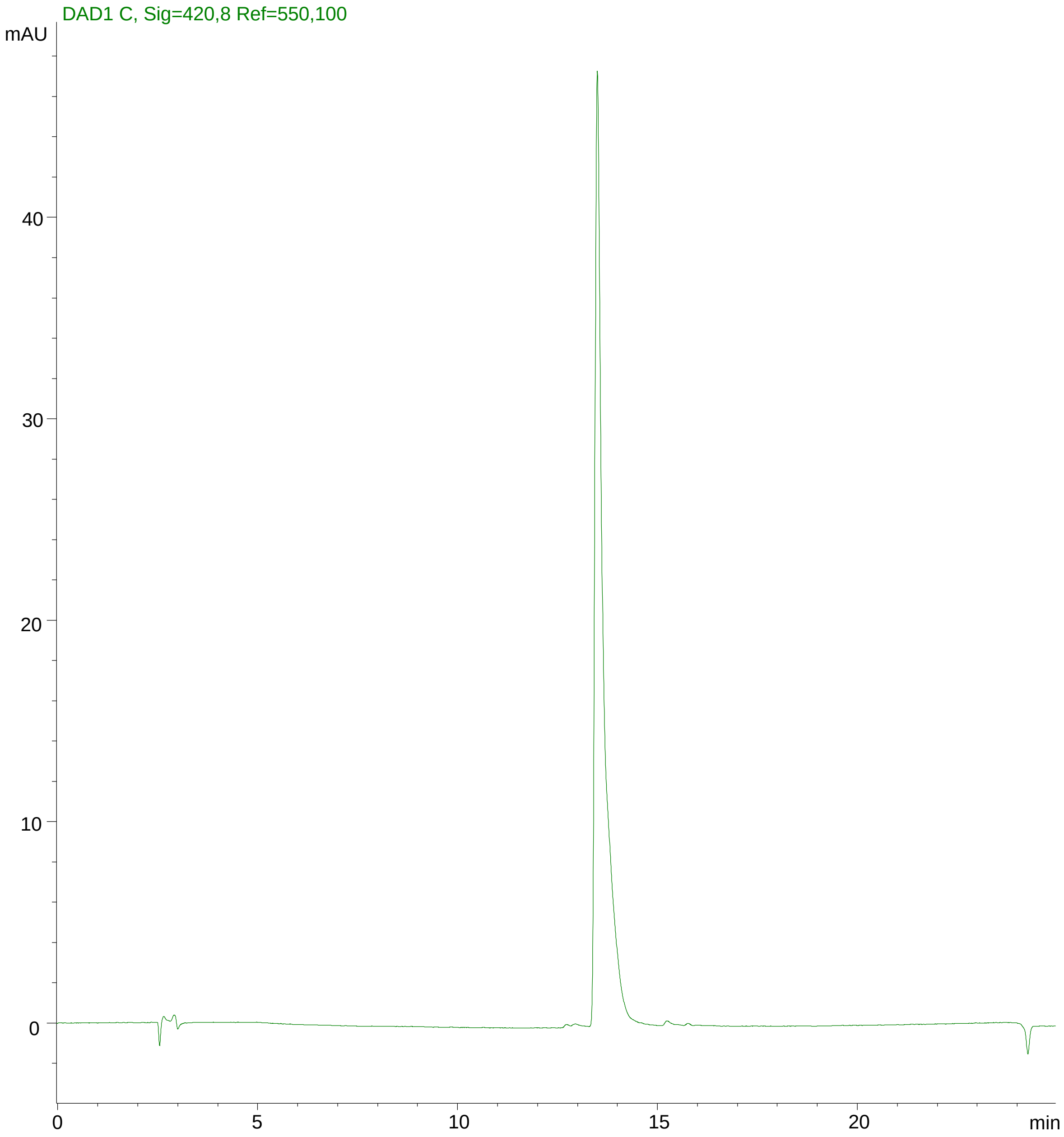

IKE7 ^{1}H NMR Spectra

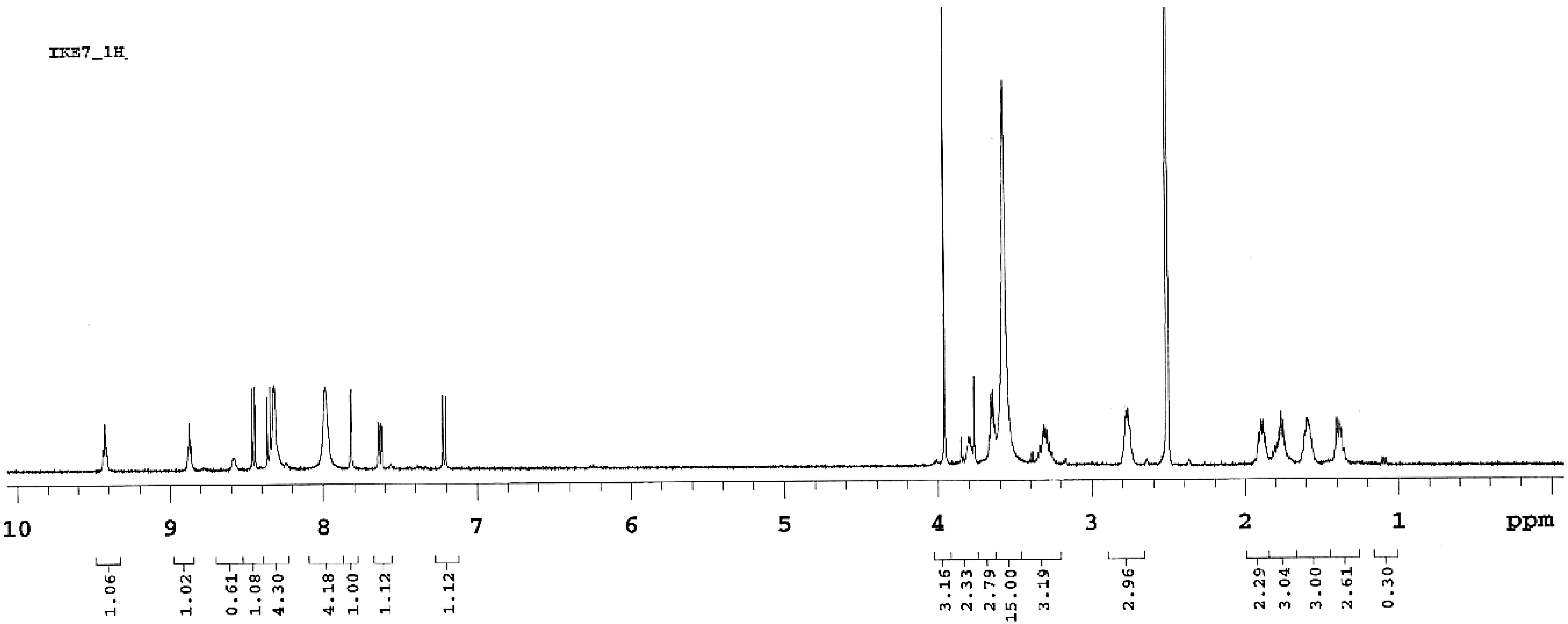

IKE7 ^{13}C NMR Spectra

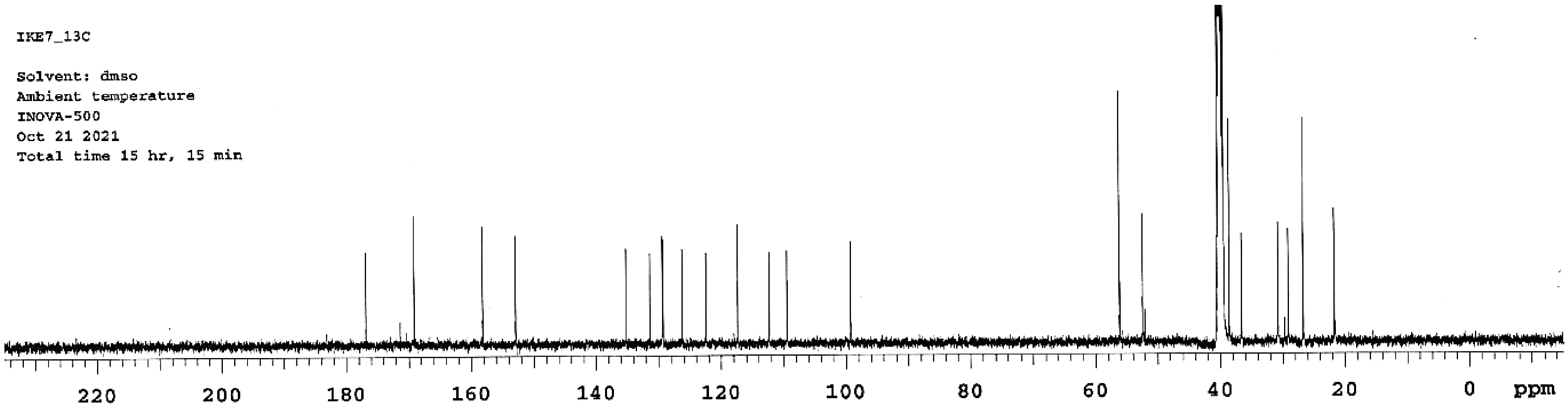

IKE7 **ESI-MS Spectra**

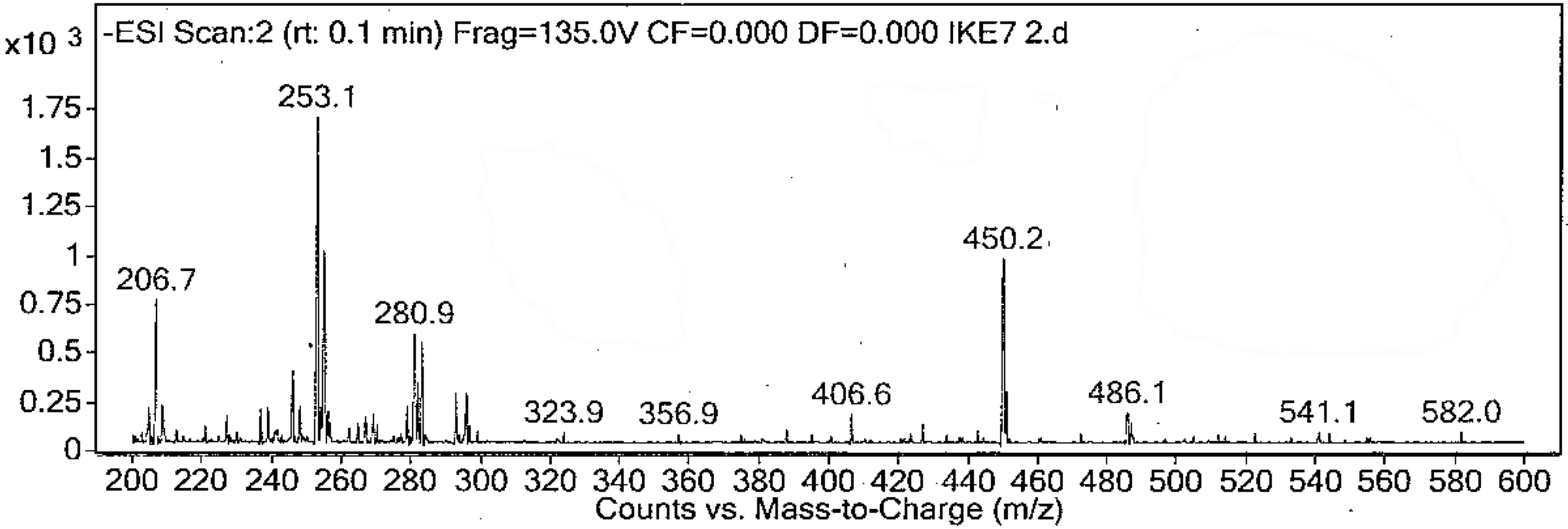

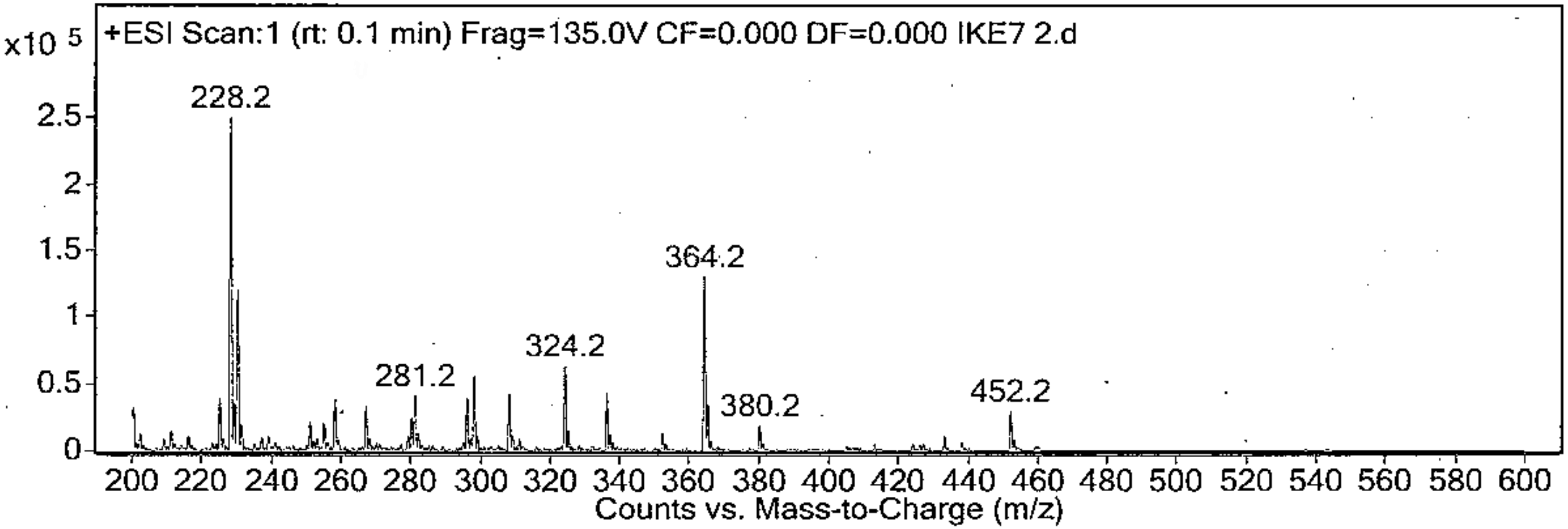

<u>IKE7</u> HPLC Spectra

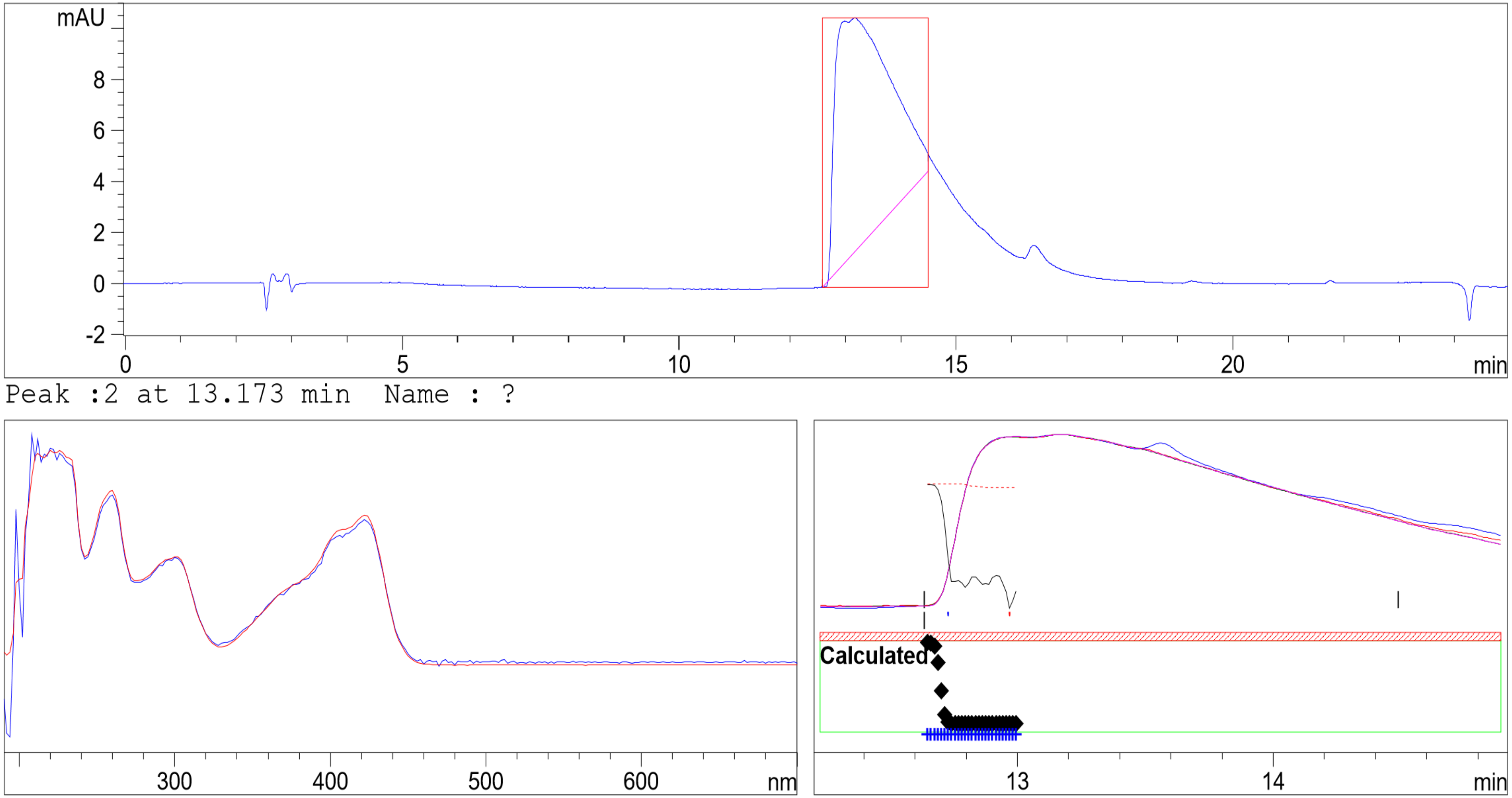

To confirm the purity of the compound, we attached the Purity of Peak file (option available in the DAD detector)

<u>IKE8</u> ^{1}**H NMR Spectra**

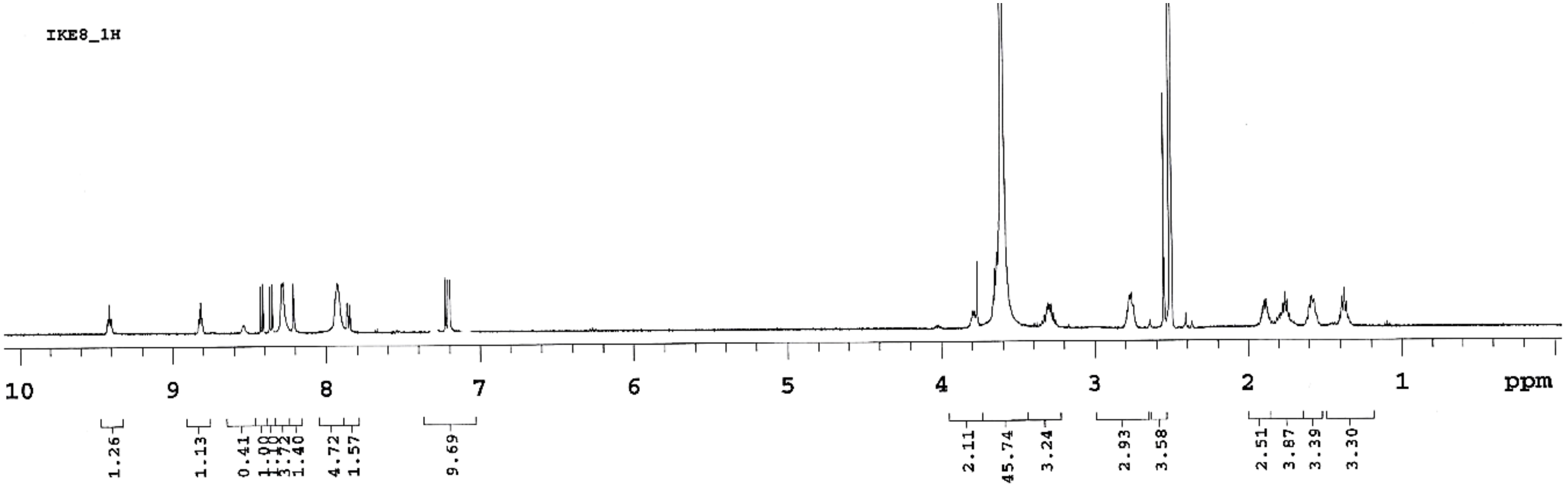

<u>IKE8</u> ^{13}**C NMR Spectra**

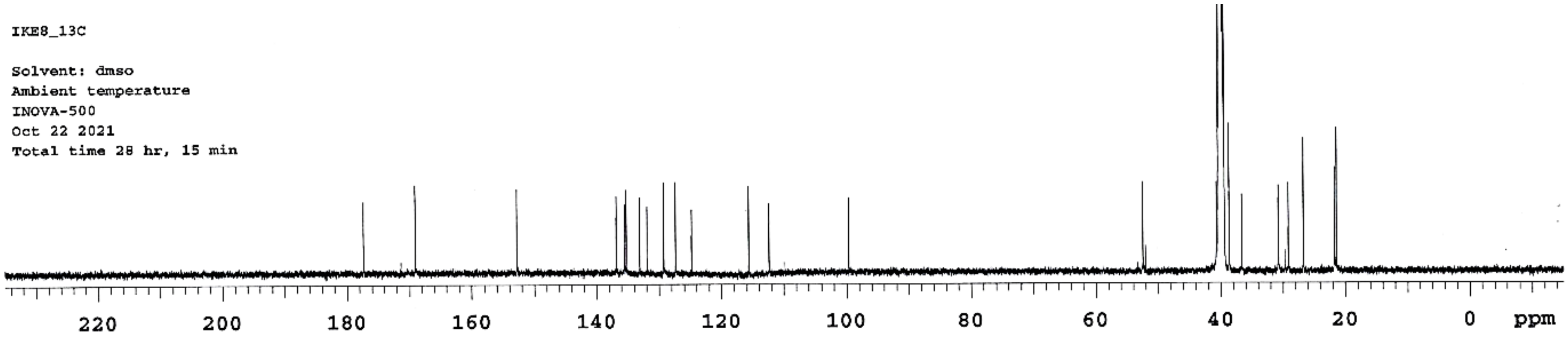

IKE8 **ESI-MS Spectra**

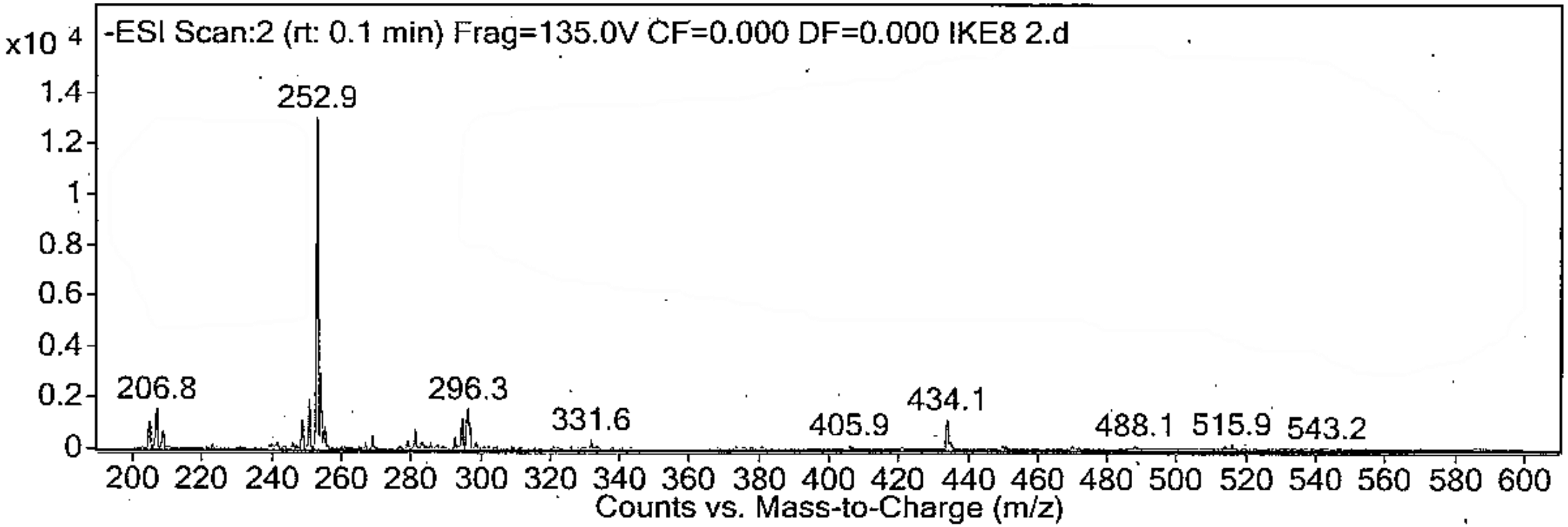

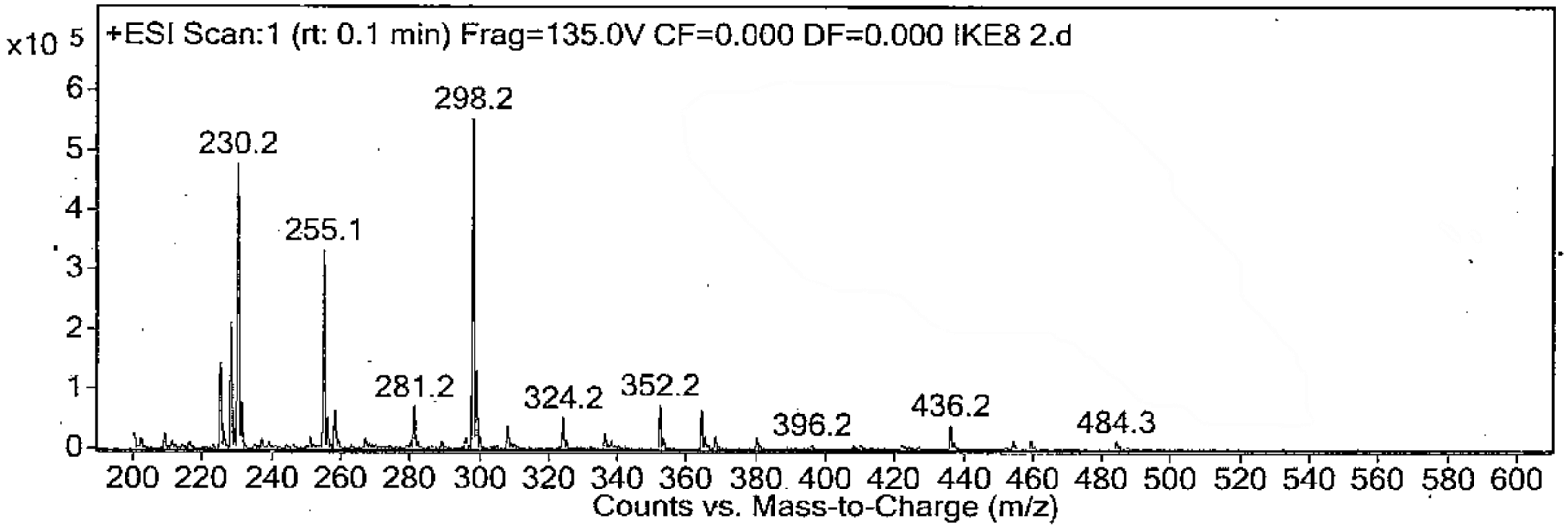

<u>IKE8</u> HPLC Spectra

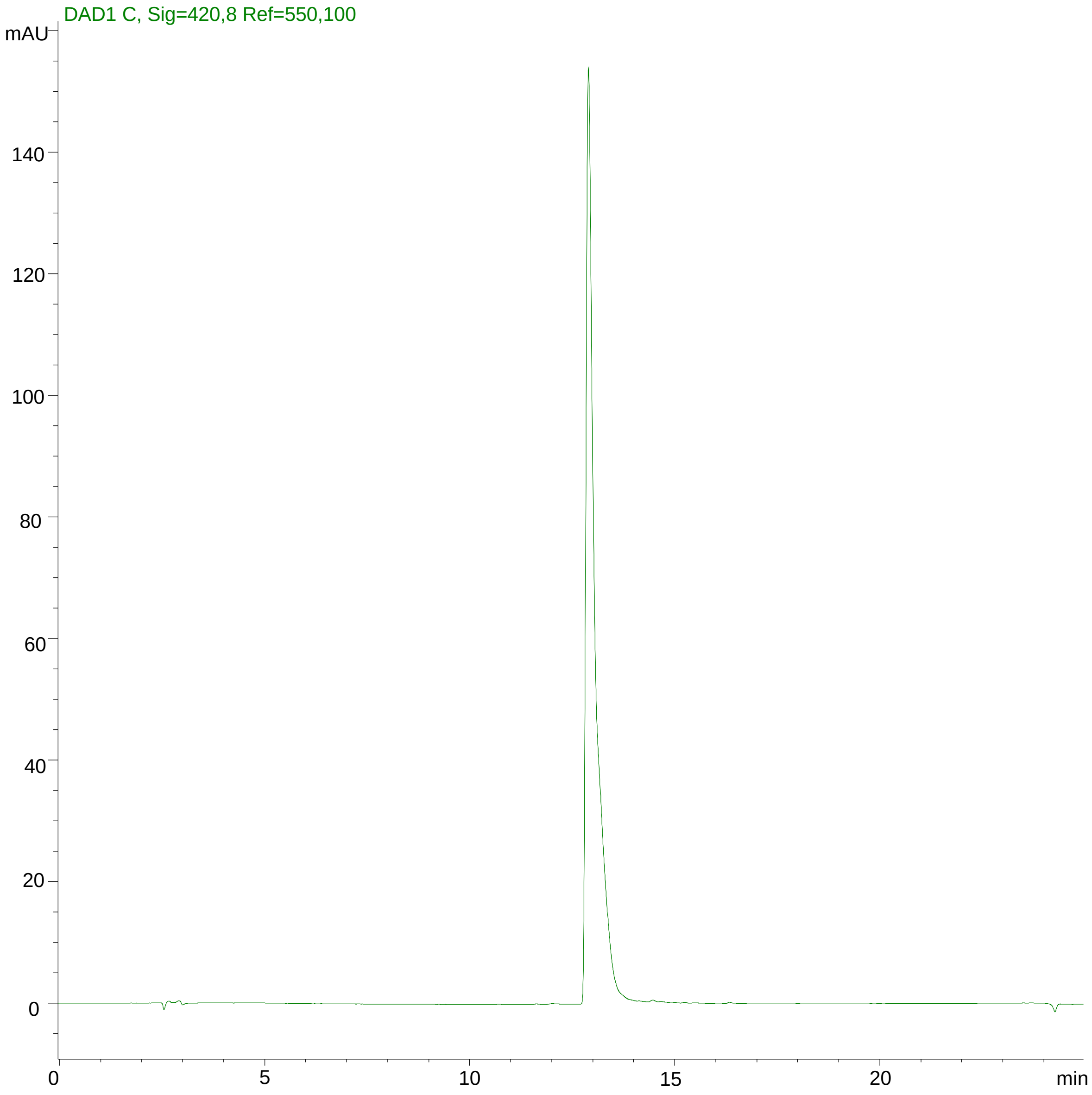

IKE9 ^{1}H NMR Spectra

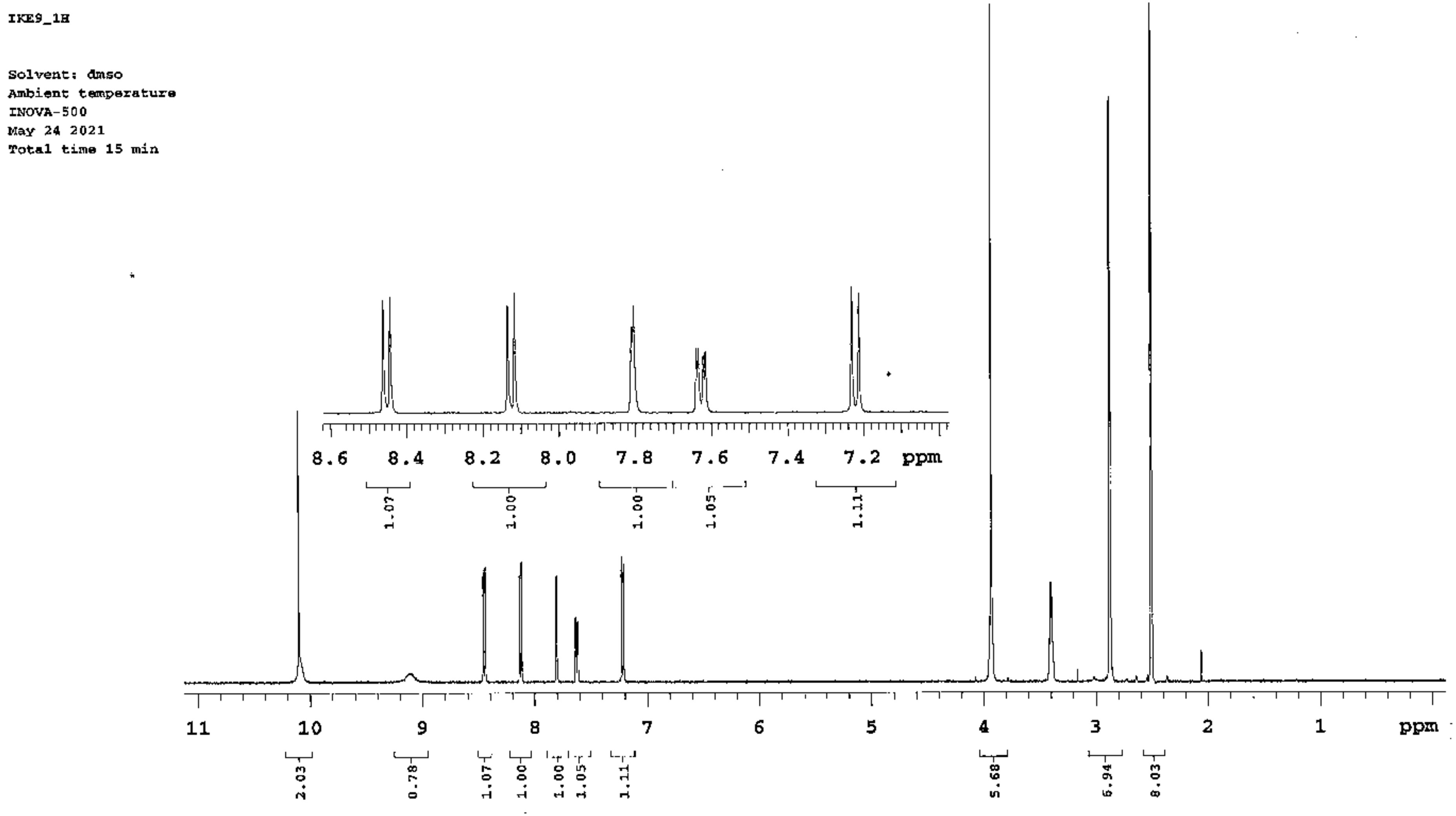

IKE9 ^{13}C NMR Spectra

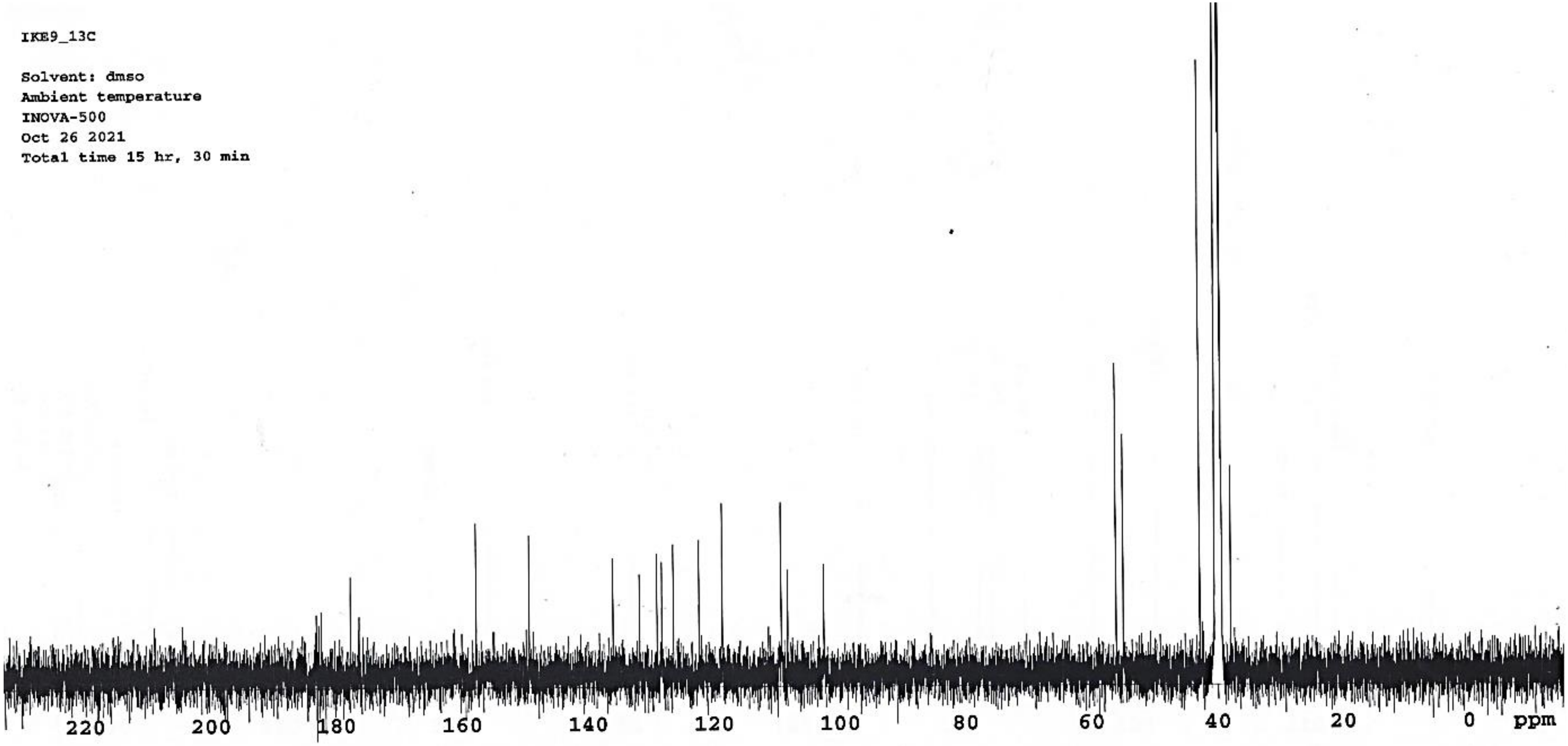

IKE9 **ESI-MS Spectra**

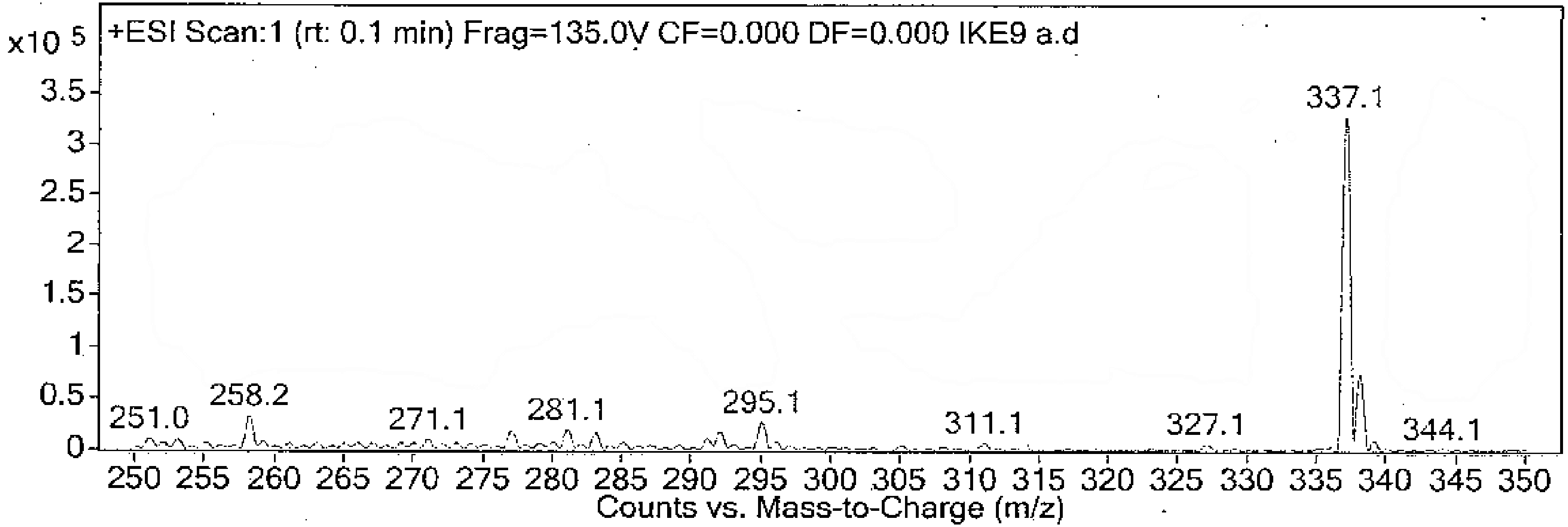

IKE9 **HPLC Spectra**

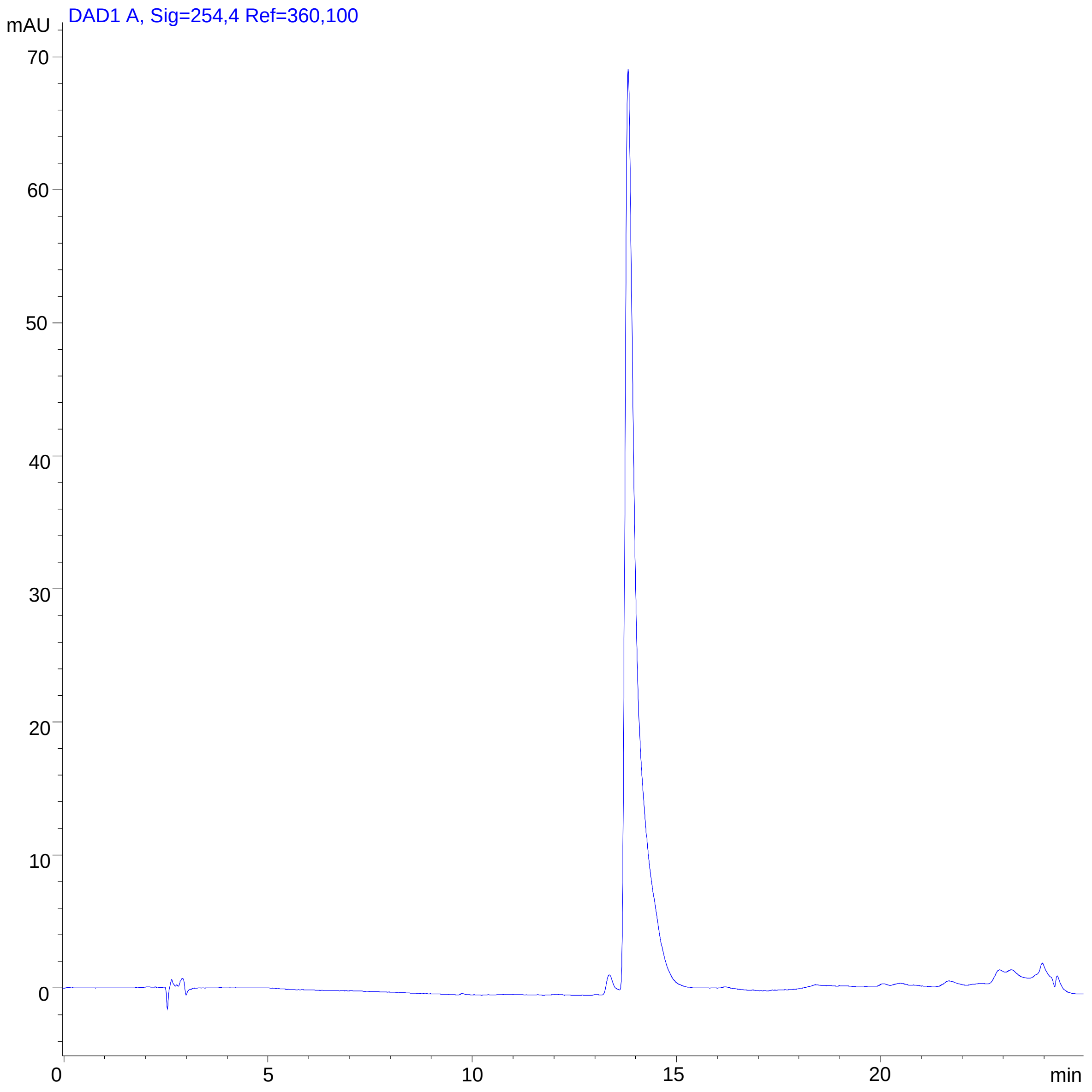

<u>IKE10</u> ^{1}**H NMR Spectra**

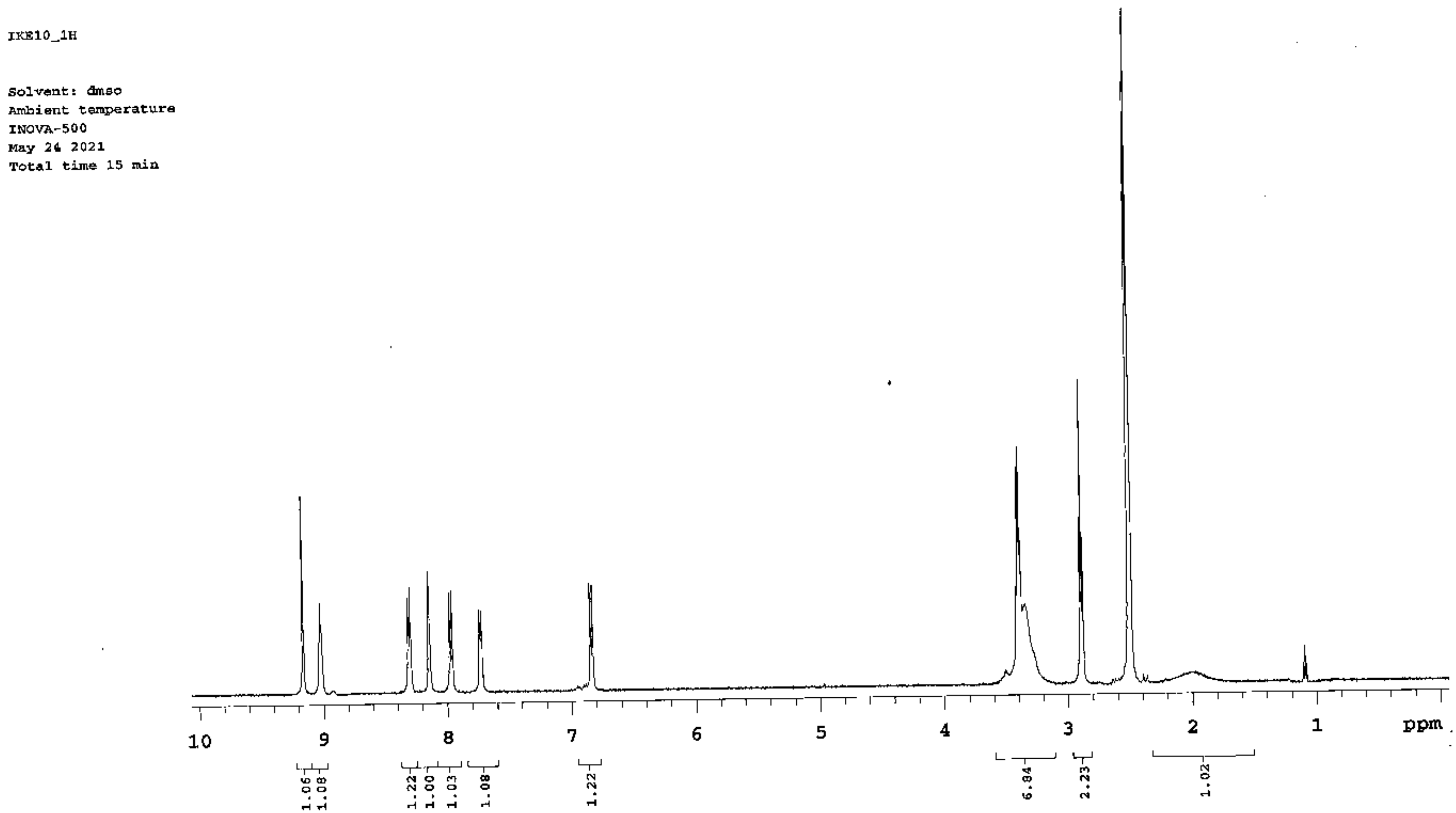

IKE10 ^{13}C NMR Spectra

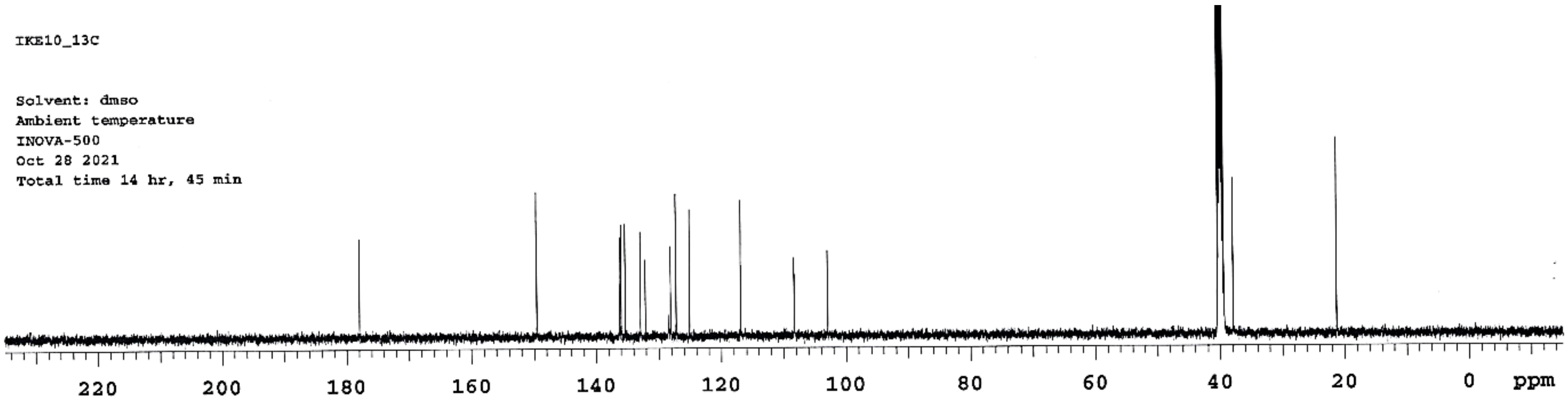

IKE10 ESI-MS Spectra

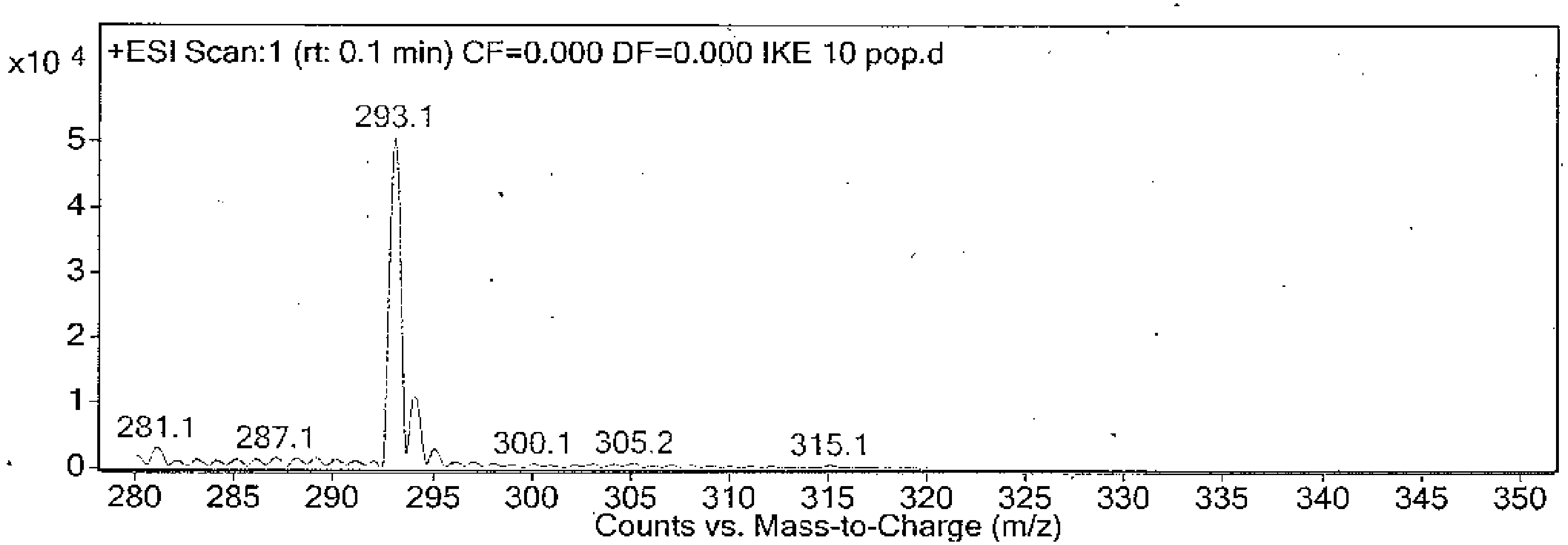

IKE10 **HPLC Spectra**

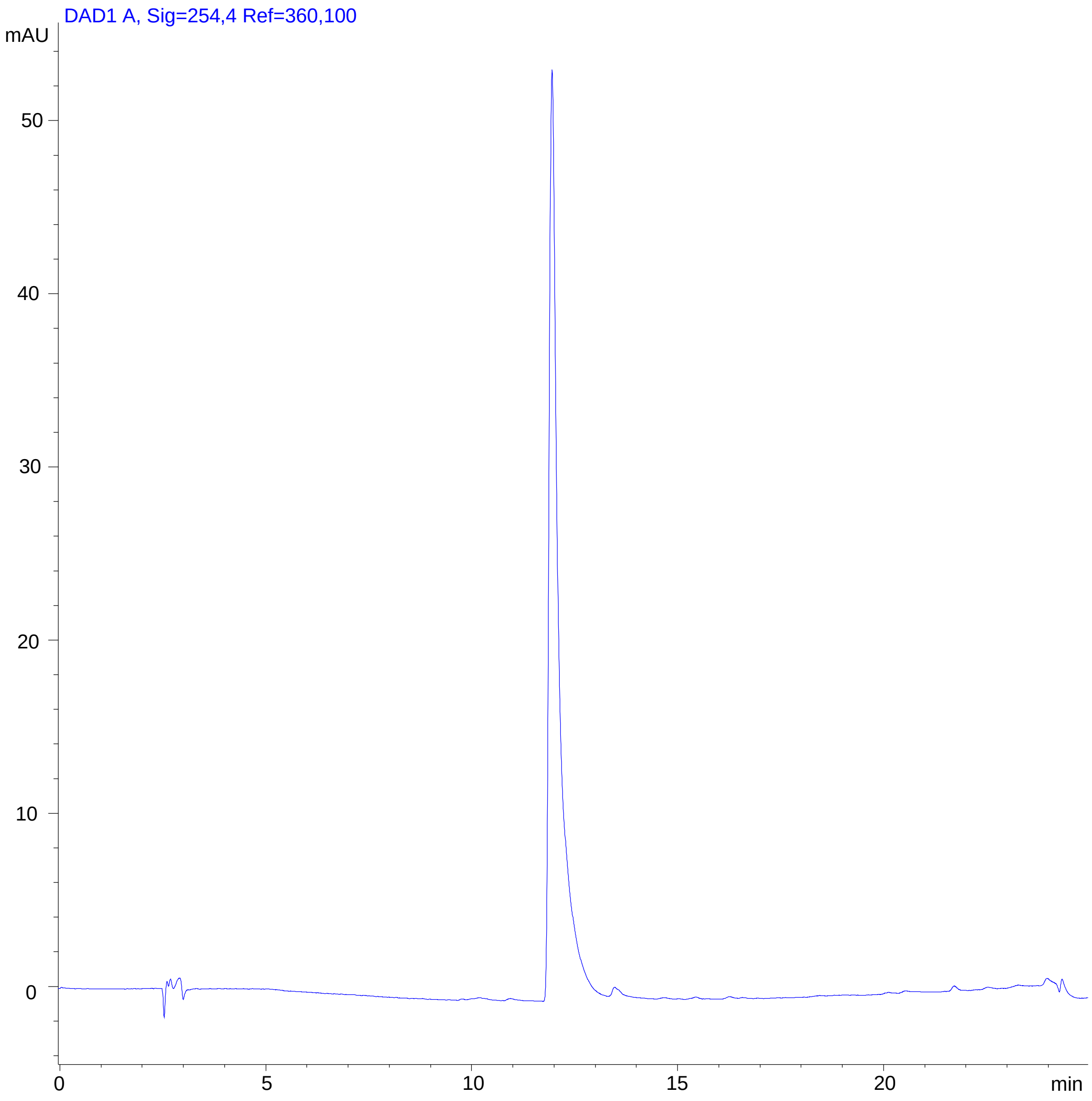

IKE11 ^{1}H NMR Spectra

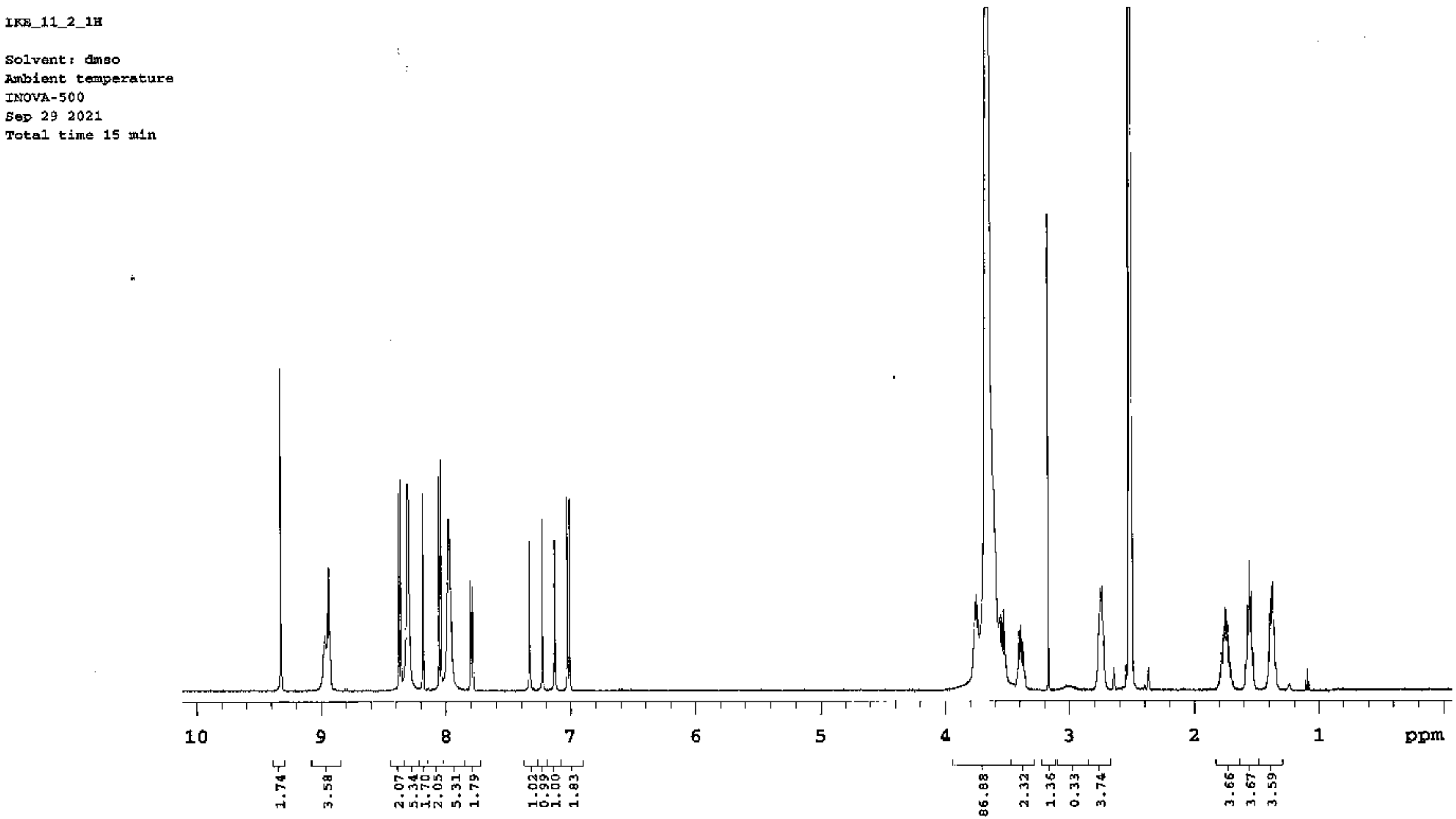

IKE11 ^{13}C NMR Spectra

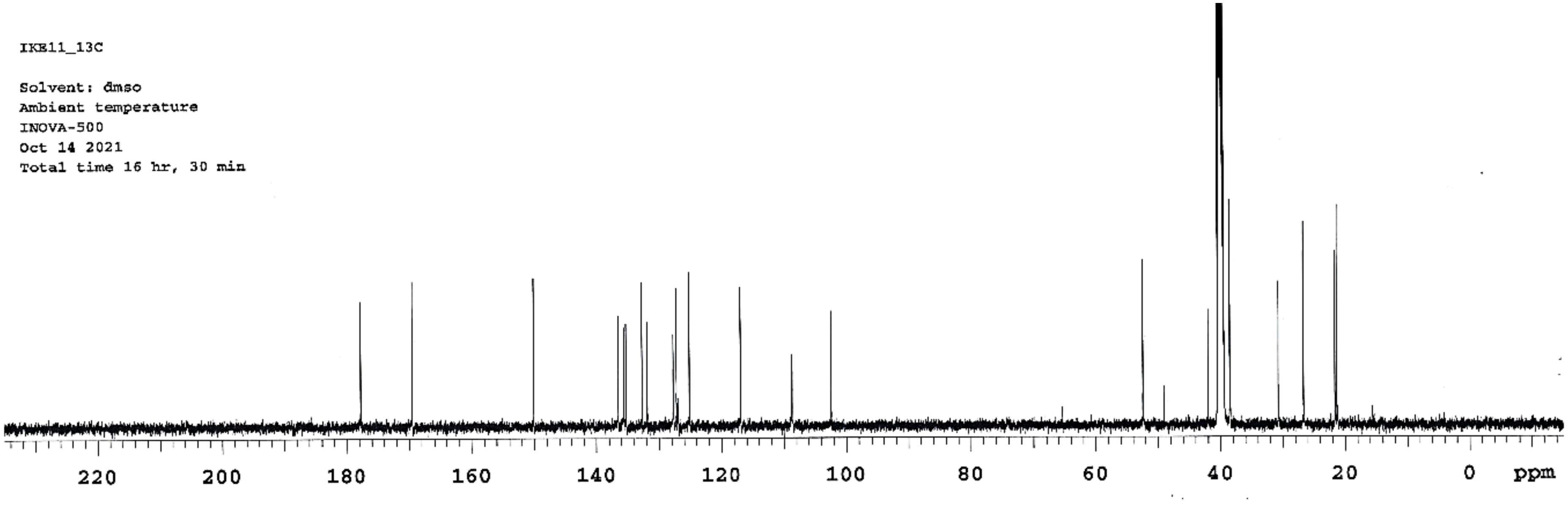

IKE11 **ESI-MS Spectra**

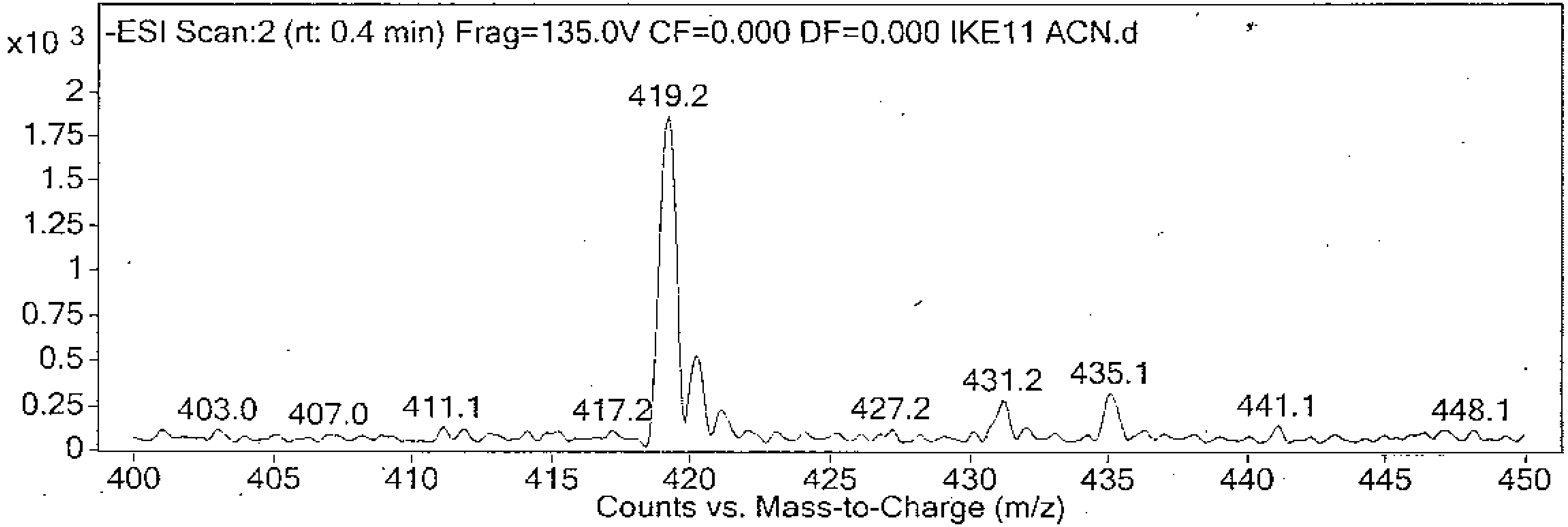

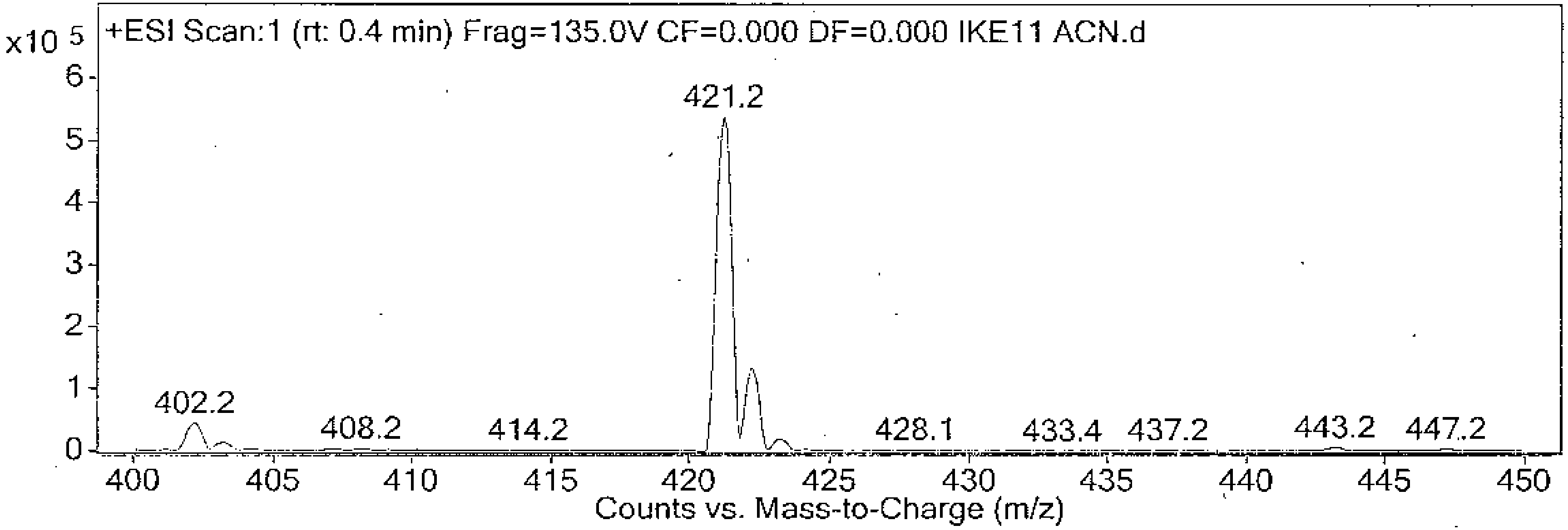

IKE11 **HPLC Spectra**

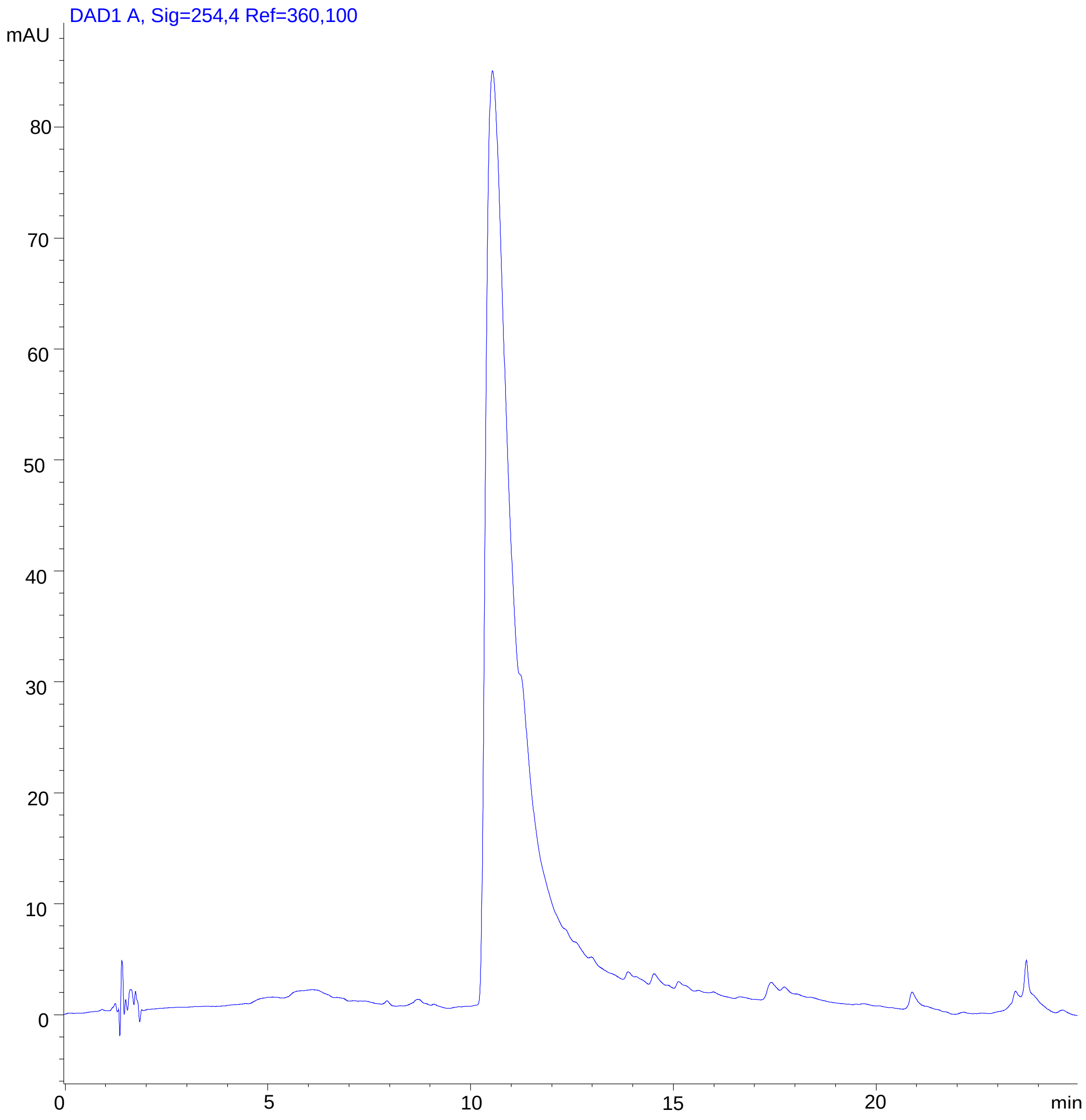

IKE12 ^{1}H NMR Spectra

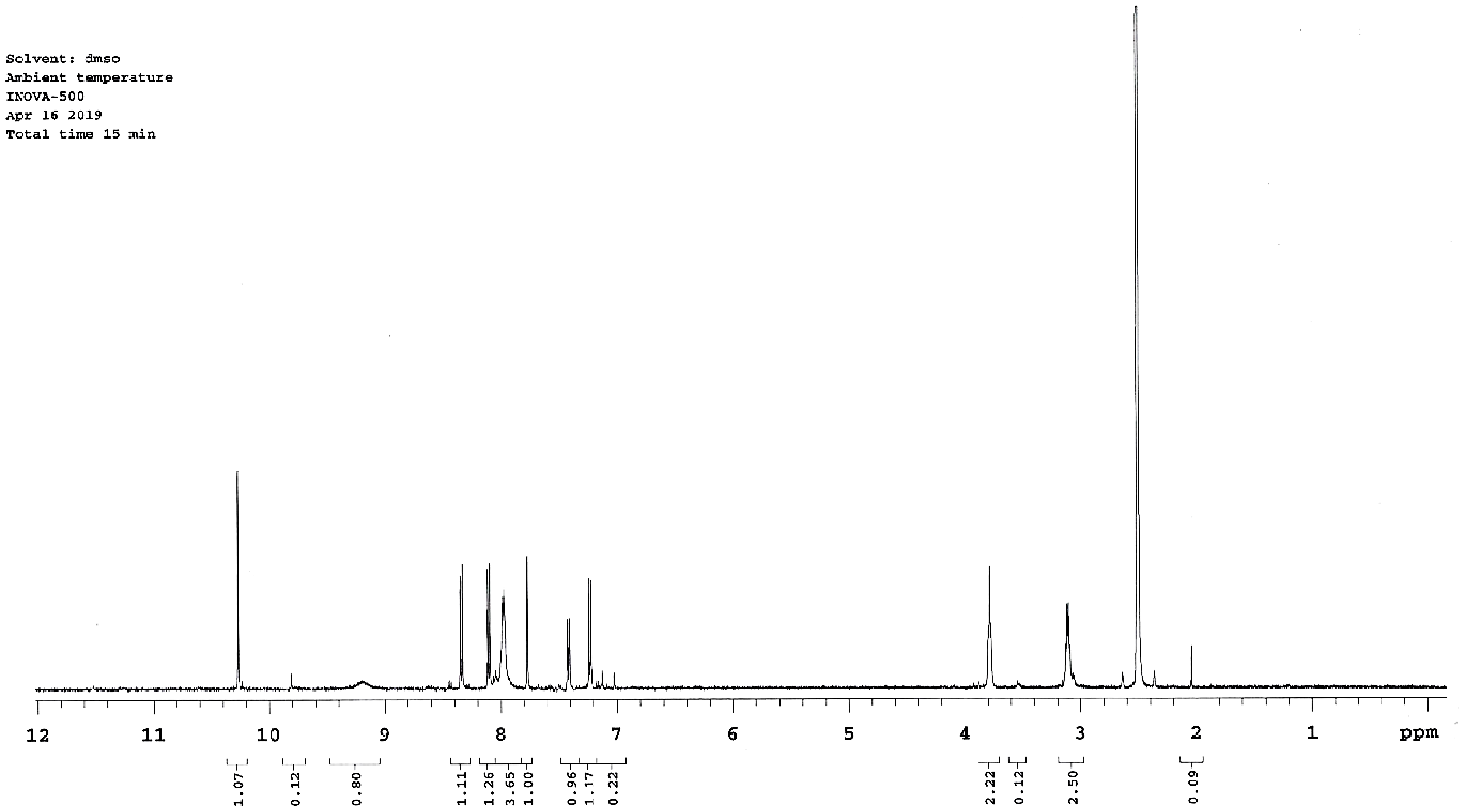

<u>IKE12</u> **^{13}C NMR Spectra**

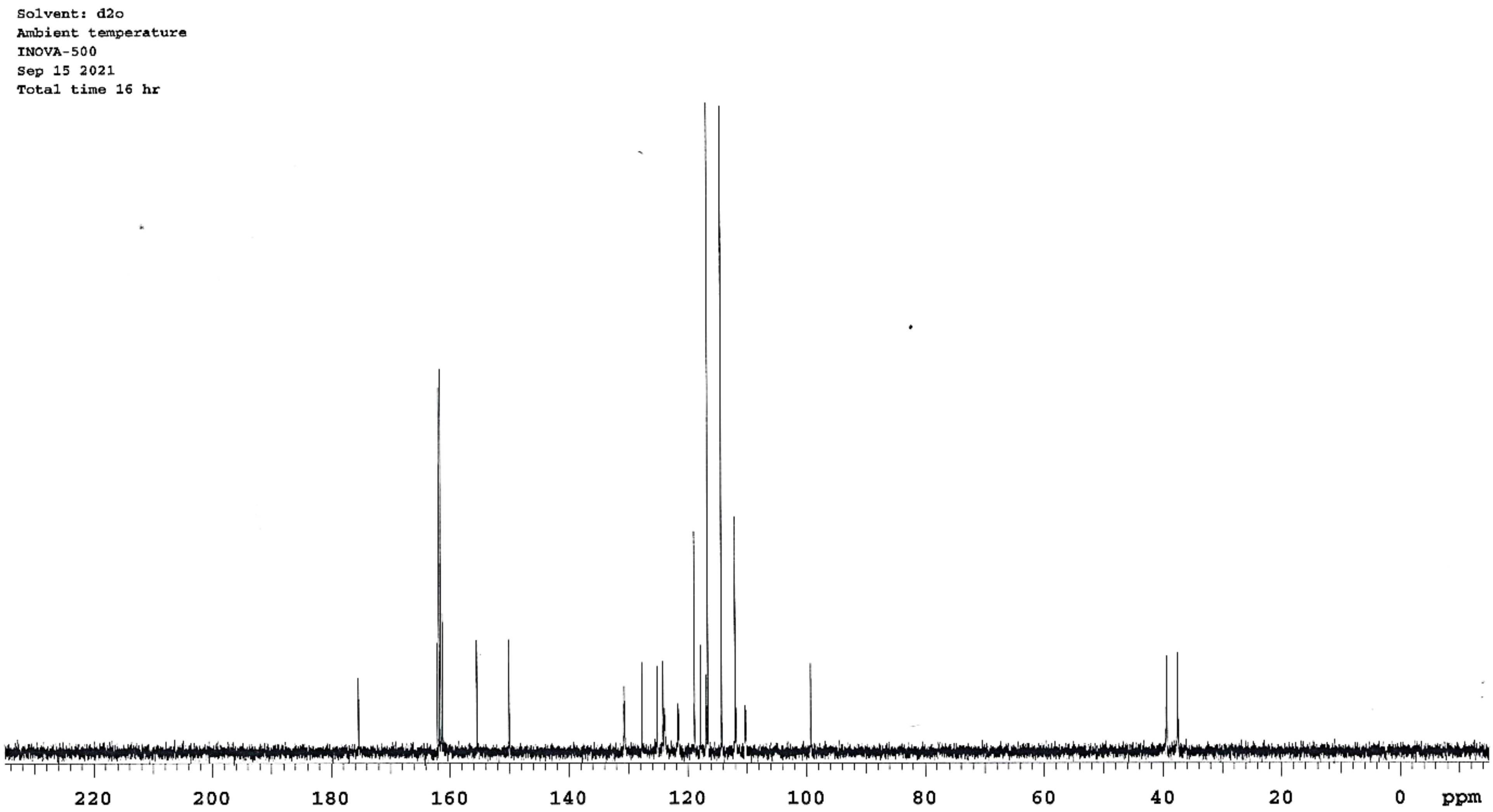

<u>IKE12</u> ESI-MS Spectra

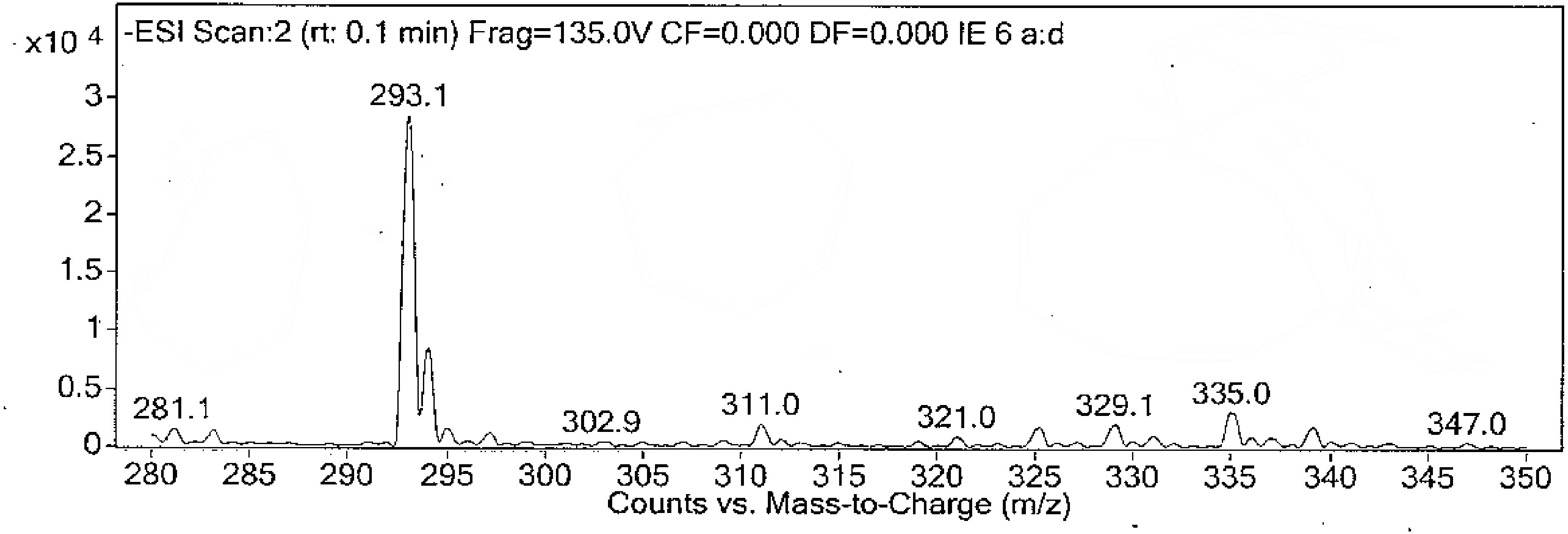

IKE12 **HPLC Spectra**

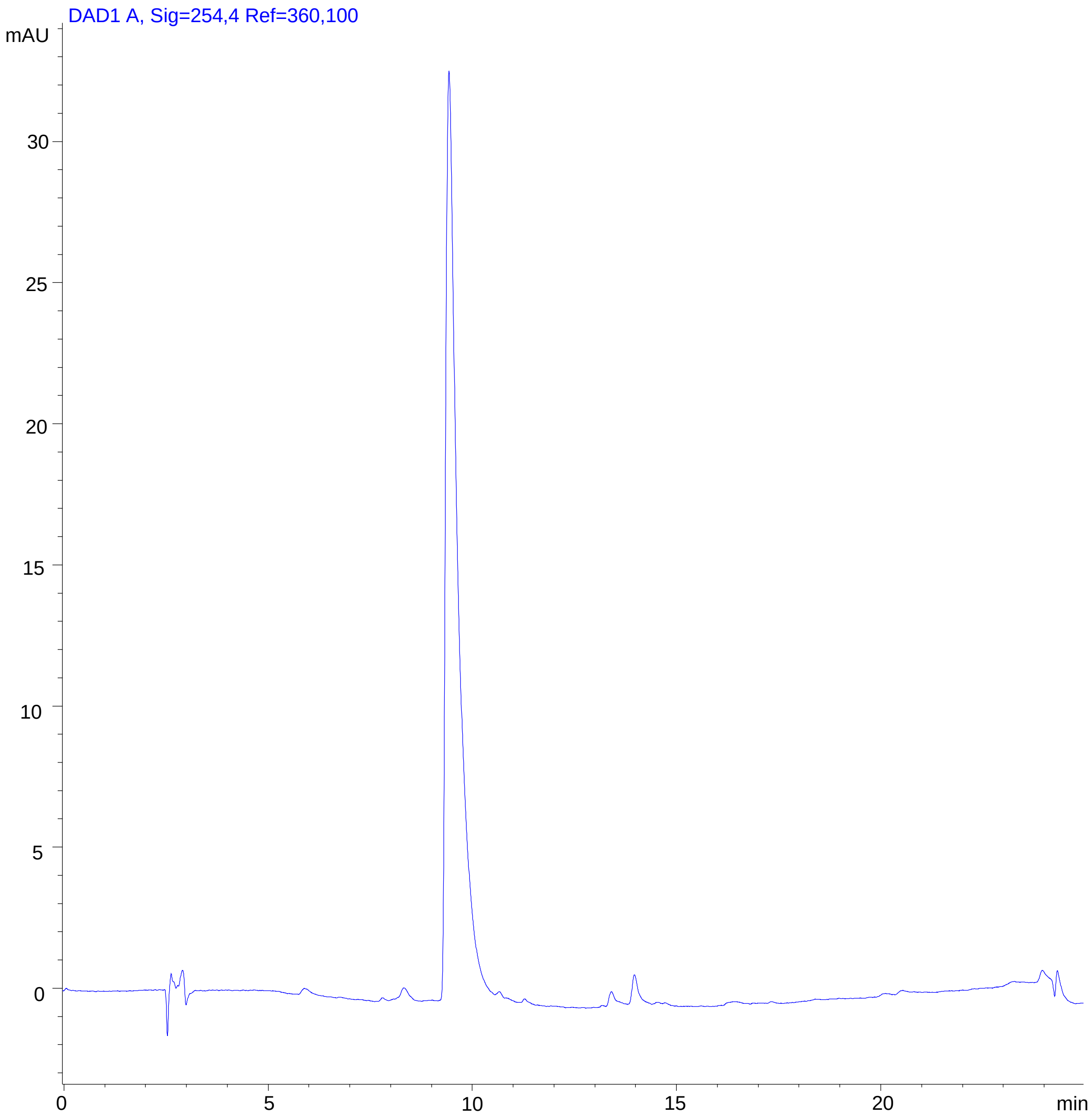

IKE13 ^{1}H NMR Spectra

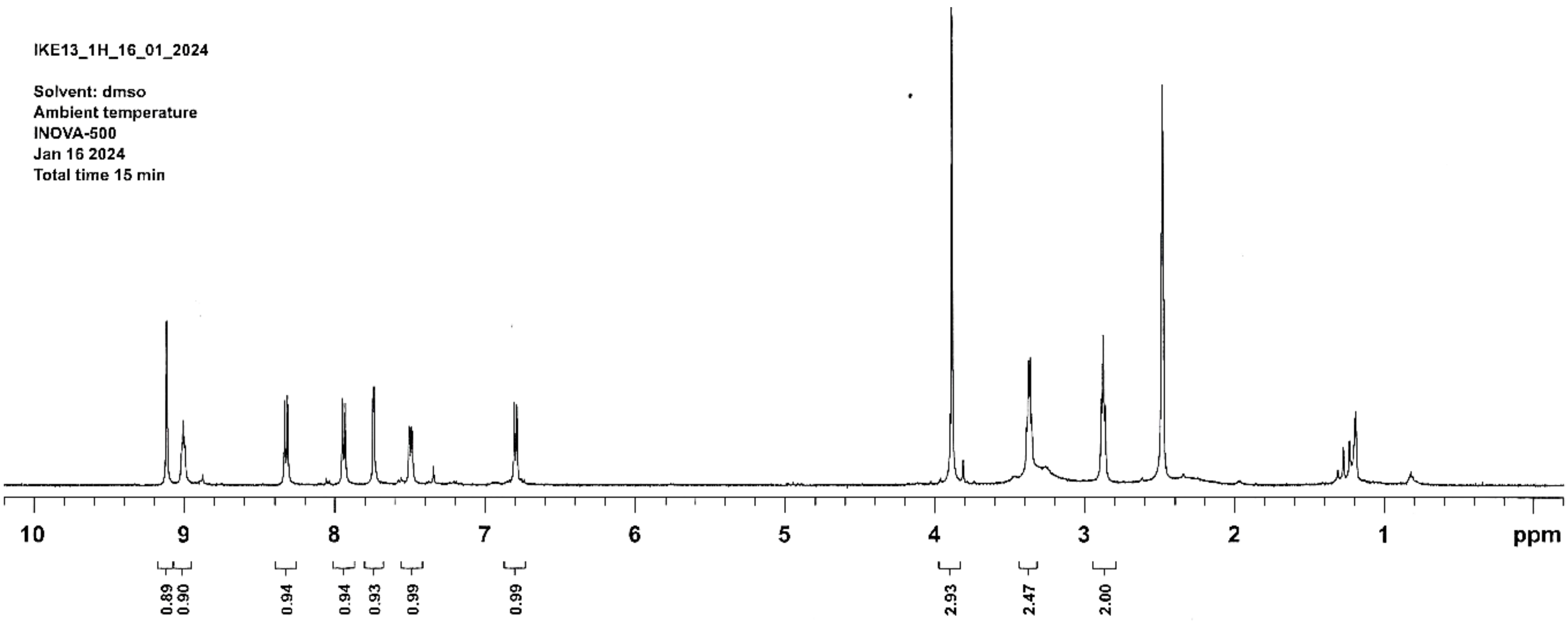

<u>IKE13</u> **^{13}C NMR Spectra**

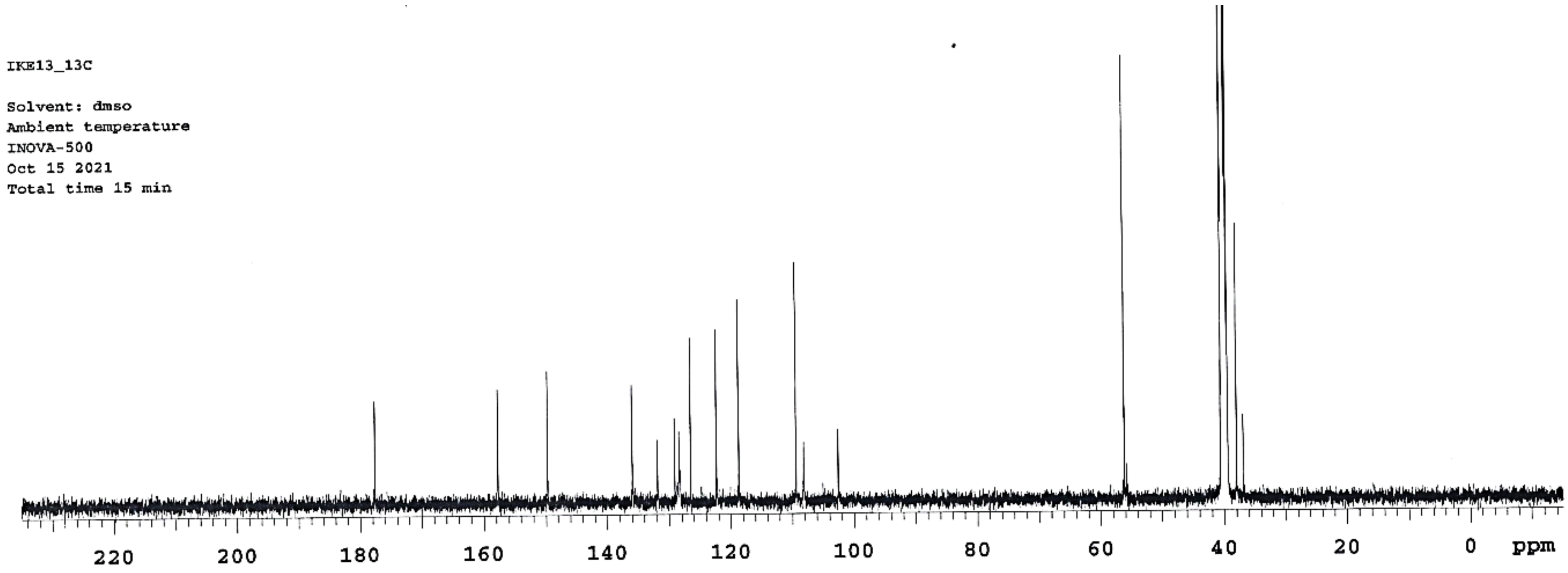

<u>IKE13</u> ESI-MS Spectra

HP

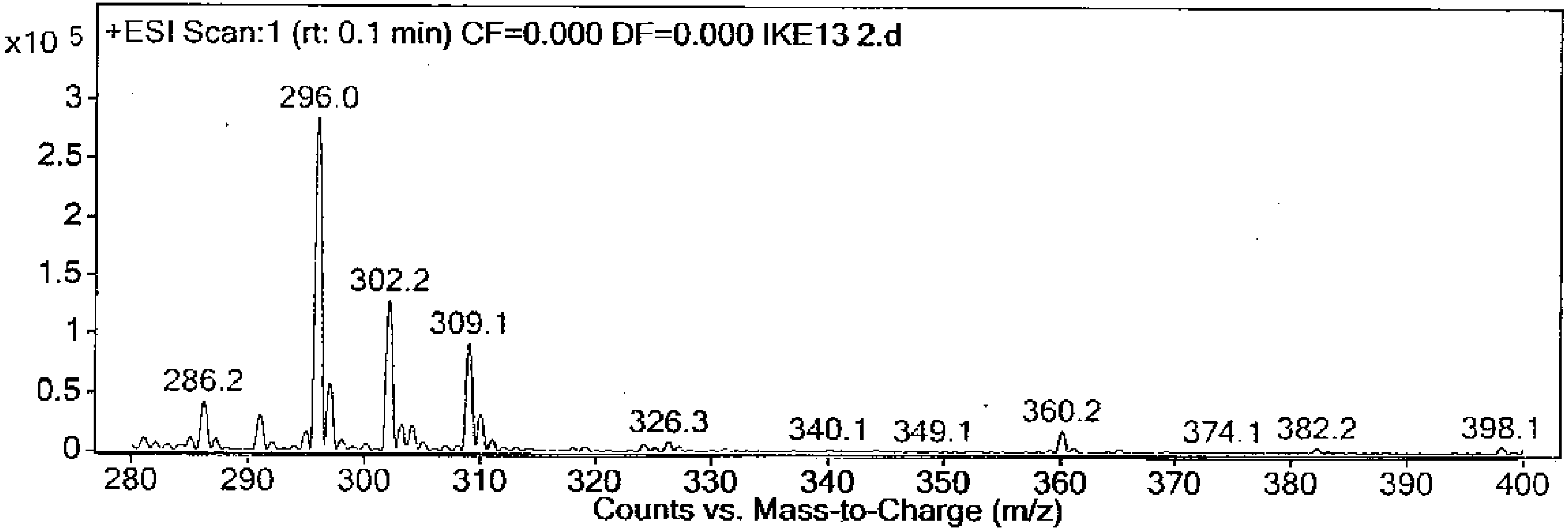

IKE13 **HPLC Spectra**

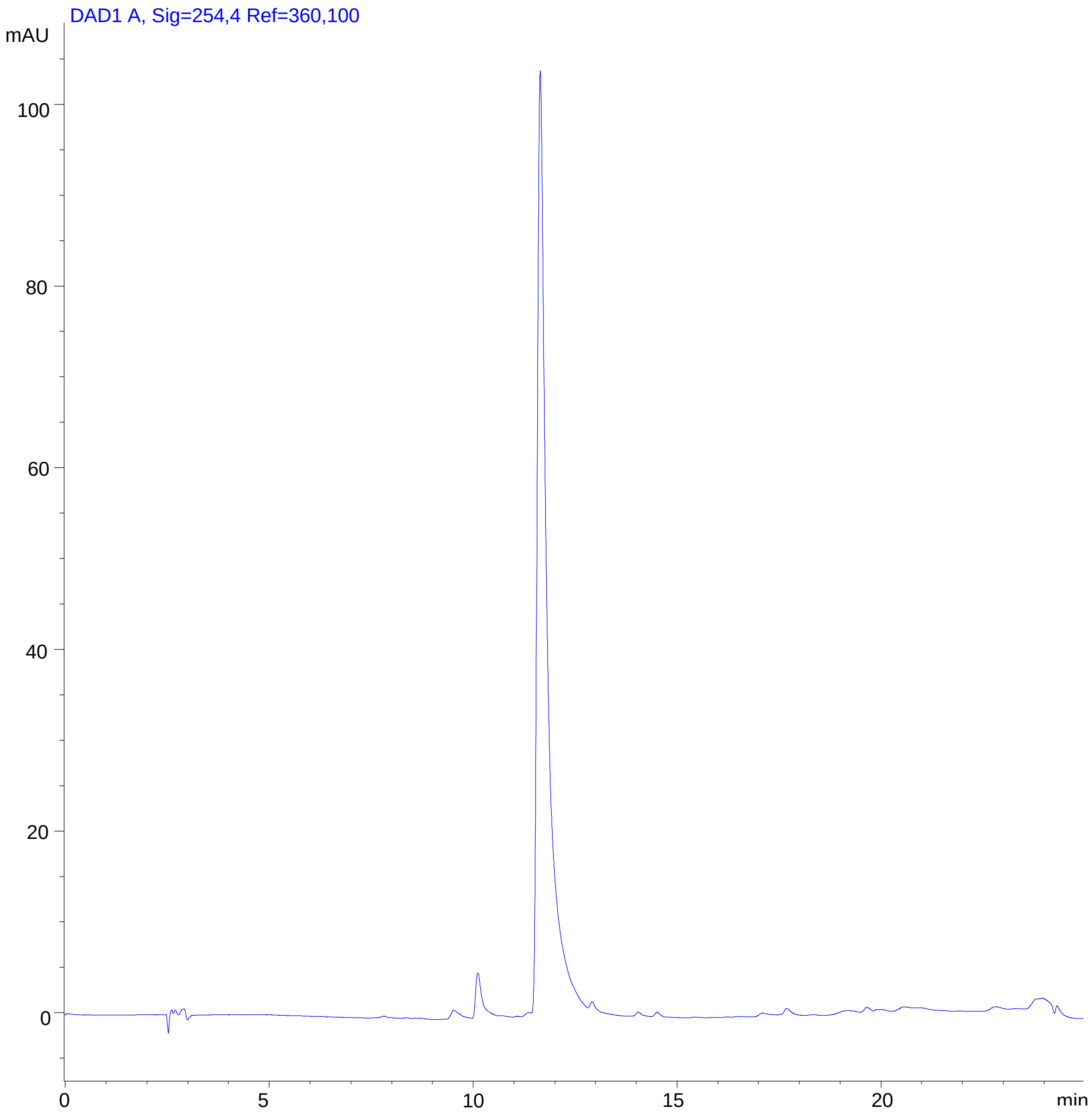

IKE14 ^{1}H NMR Spectra

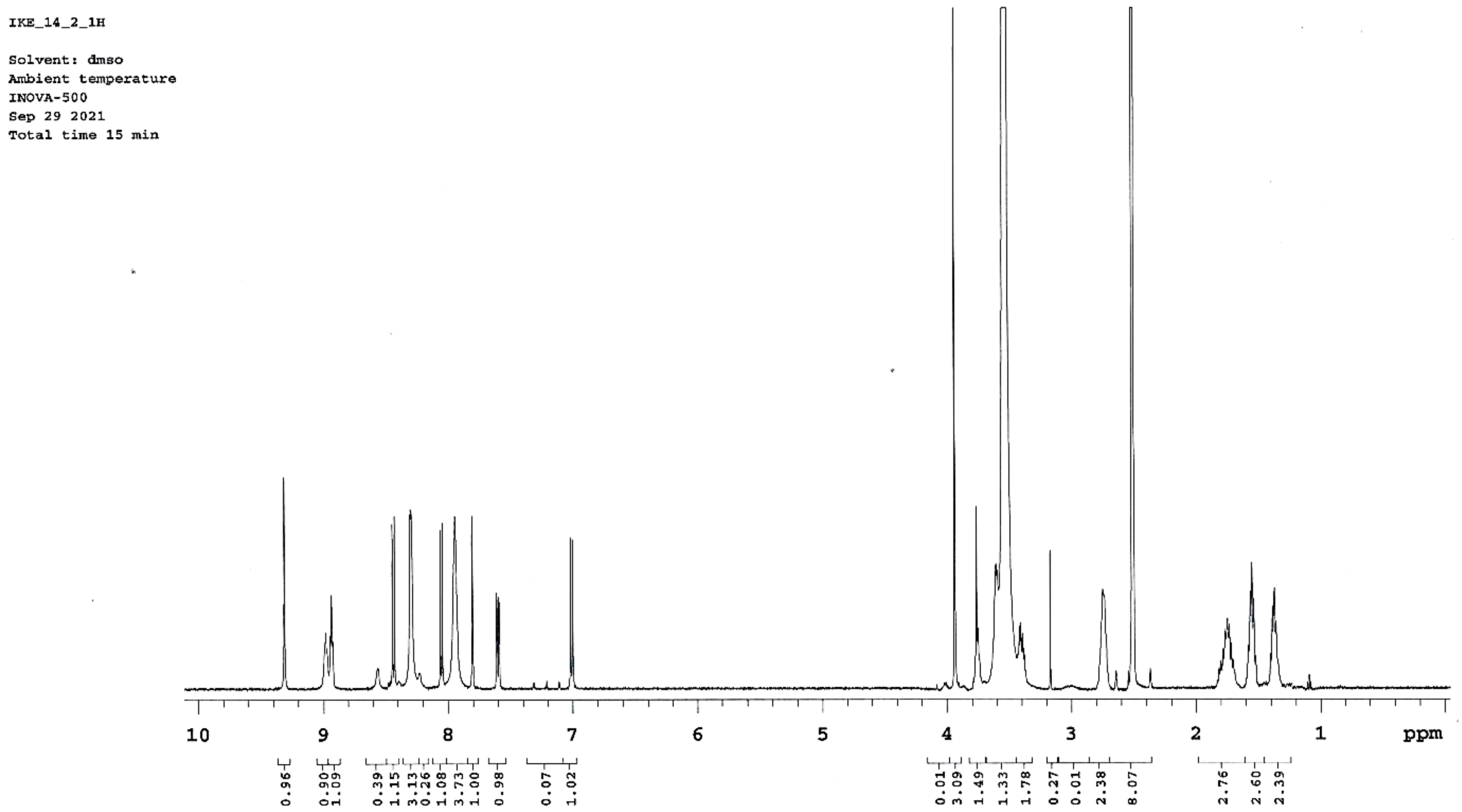

IKE14 ^{13}C NMR Spectra

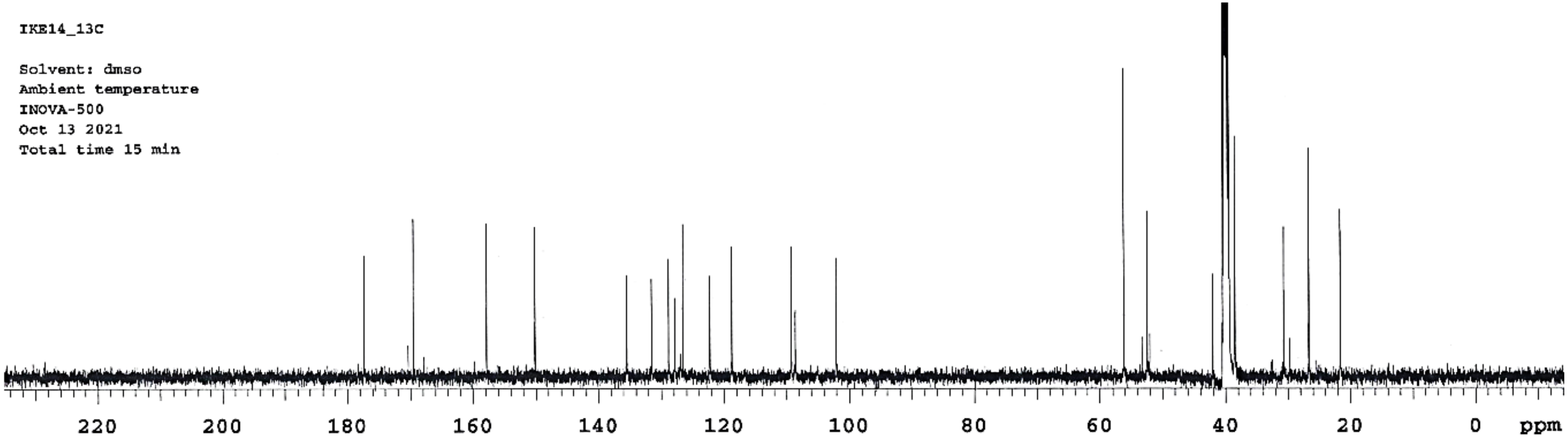

IKE14 ESI-MS Spectra

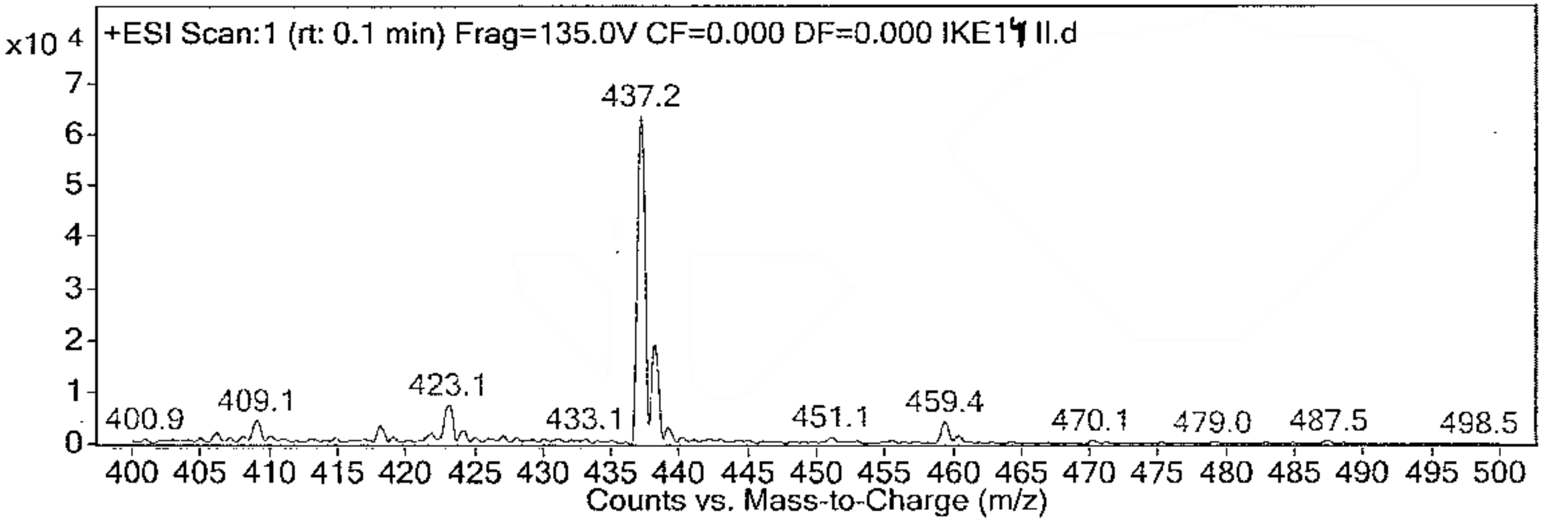

<u>IKE14</u> HPLC Spectra

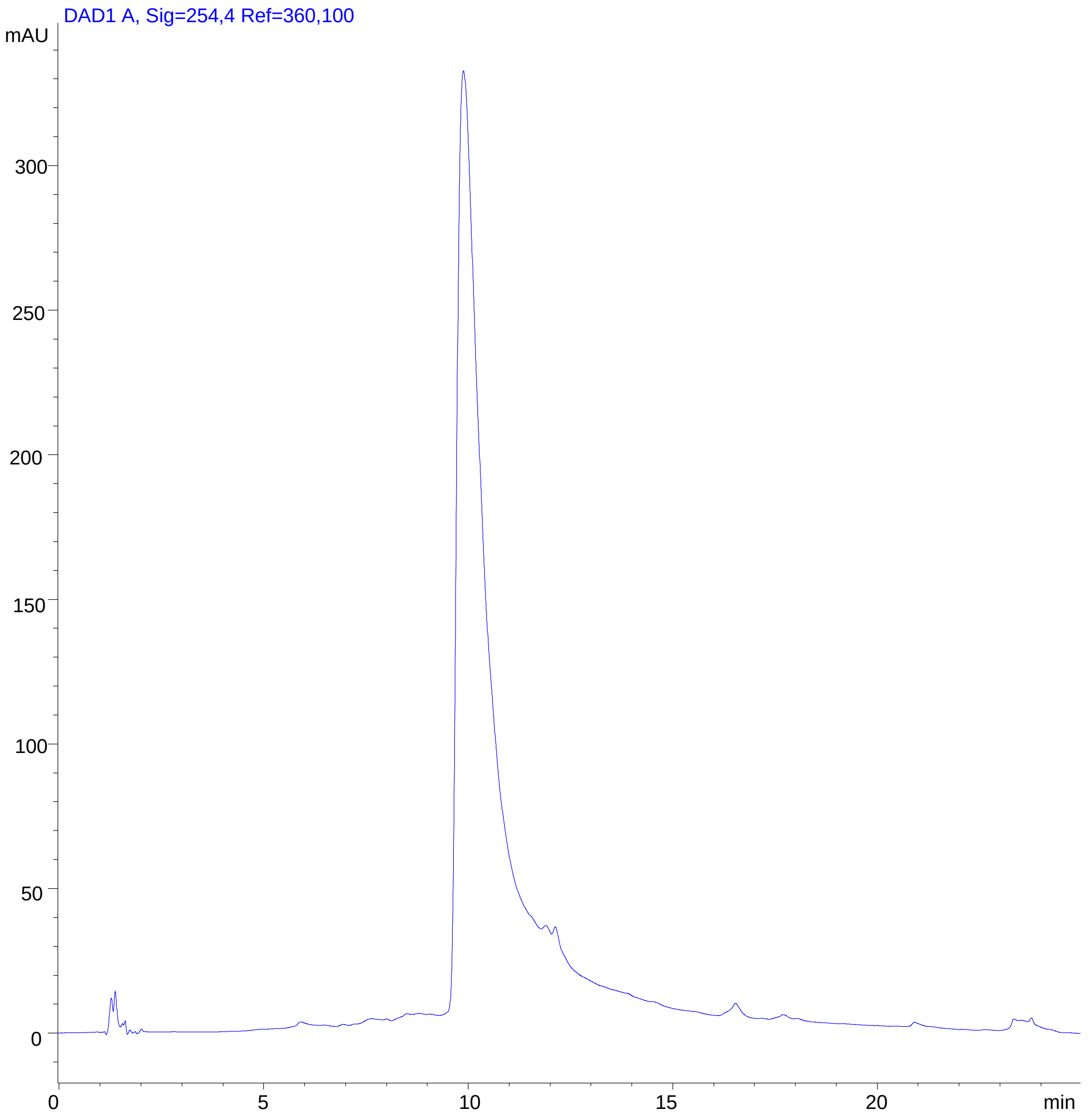

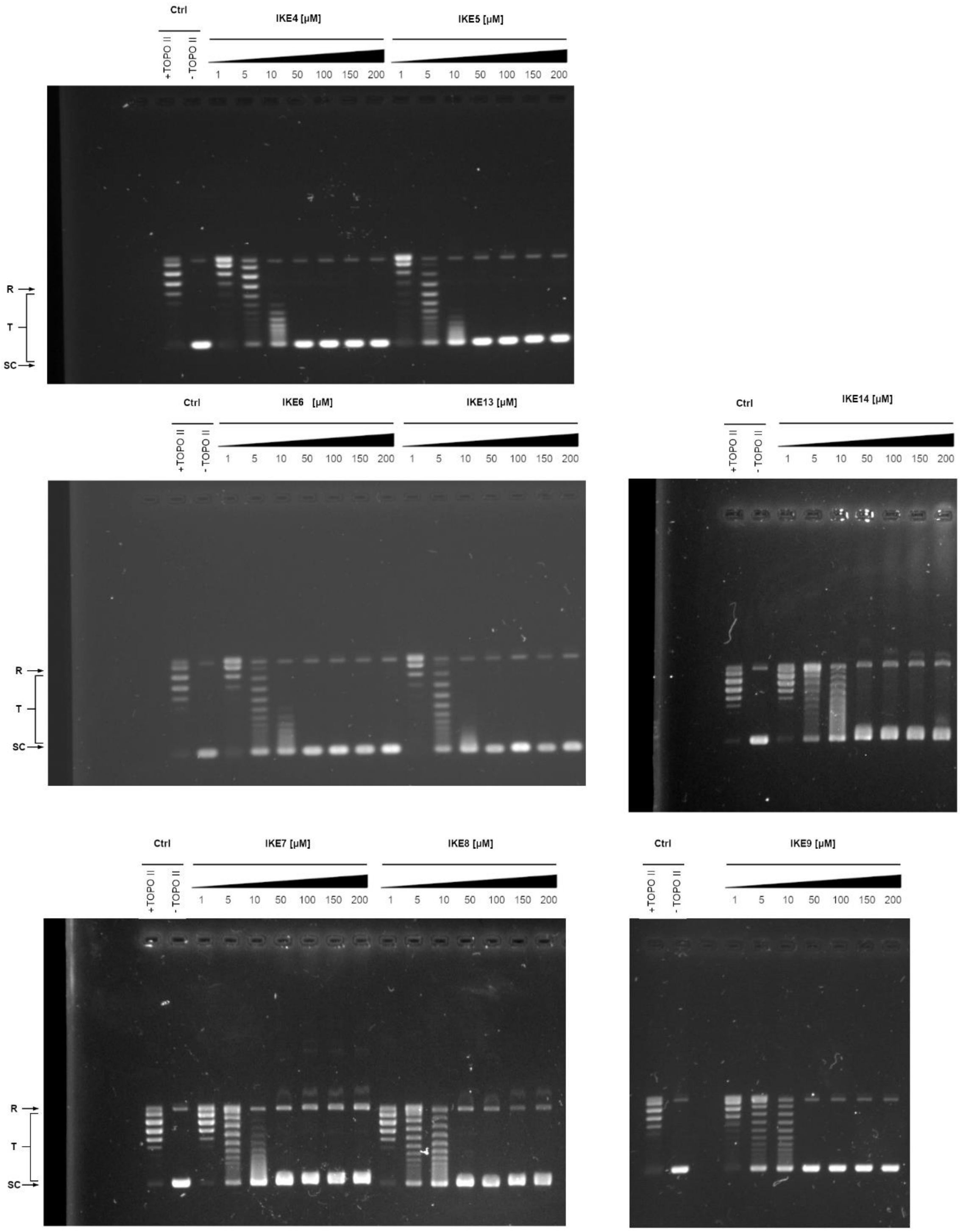

Figure S3. Inhibition of the catalytic activity of purified yeast topoisomerase II by selected compounds: IKE4, IKE5, IKE6, IKE7, IKE8, IKE9, IKE13 and IKE14 as measured by relaxation. Supercoiled pBR322 plasmid DNA (lane 1, -TOPO II) was relaxed by purified yeast topoisomerase II in the absence (lane 2, +TOPO II) or presence of an analyzed compound at 1, 5, 10, 50, 100, 150, or 200 µM. The resulting topological forms of DNA were separated by gel electrophoresis. SC, supercoiled DNA; R, relaxed DNA; T, DNA topoisomers. DNA was separated in a 1% agarose gel. The data shown are typical of three independent experiments.

9

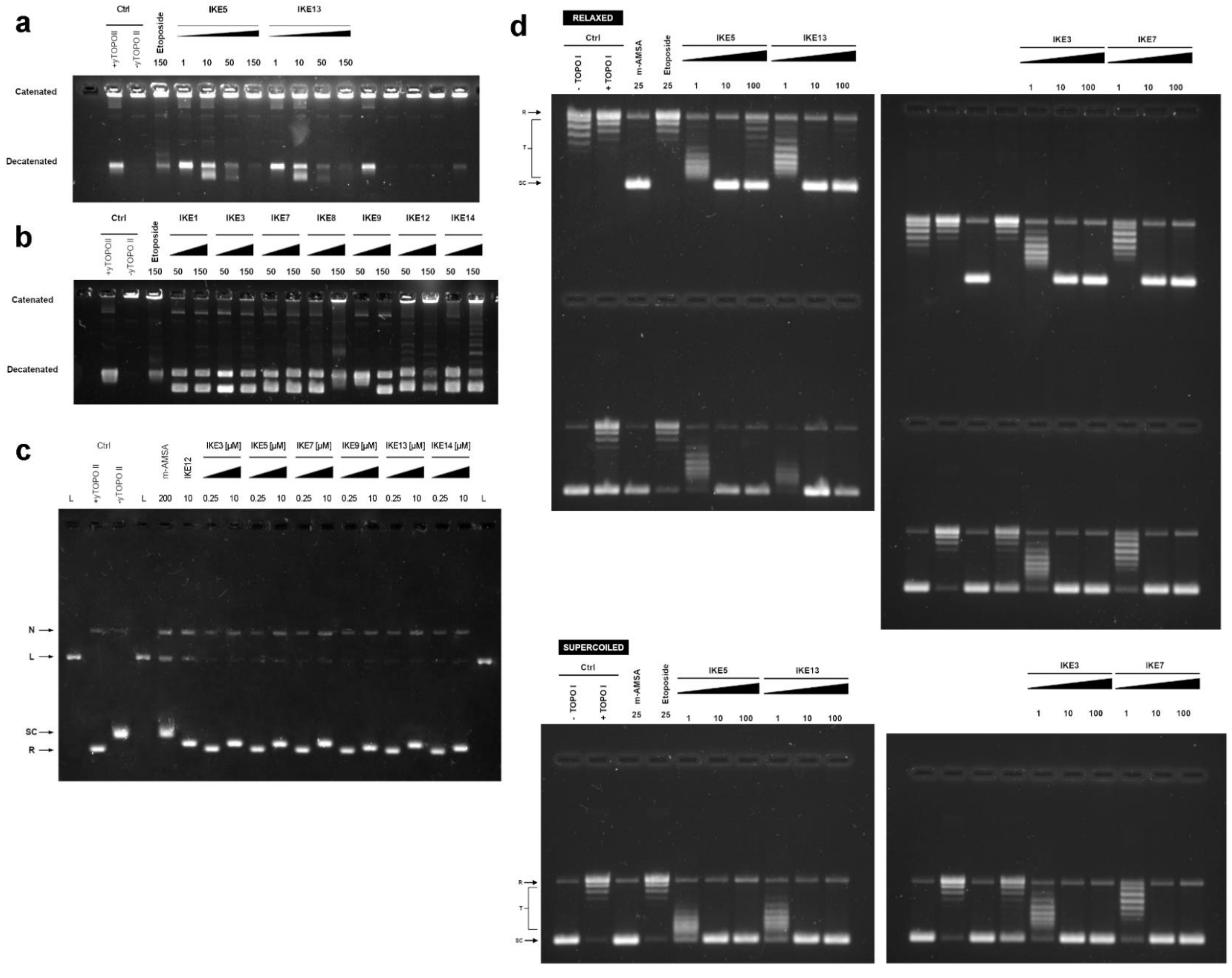

Figure S4. Full length gels. **a, b** Inhibition of the catalytic activity of purified yeast topoisomerase II by selected compounds as measured decatenation of kinetoplast DNA (catenated). Kinetoplast DNA (-yTOPO II) was decatenated by yeast topoisomerase II in the absence (+yTOPO II) or presence of etoposide (lane 3, 150 µM) or selected compounds; **c** The influence of tested compounds on the formation of covalent DNA-yeast topoisomerase II complexes. Supercoiled pBR322 plasmid DNA (lane 2, -yTOPO II) was incubated with purified yeast topoisomerase II in the absence (lane 1, +yTOPO II) or in the presence of 200 µM m-AMSA (lane 4) or with selected compounds (lanes 5–17). Lane 3, linearized pBR322 DNA. SC, supercoiled DNA; R, relaxed DNA; L, linear DNA; N, nicked circular DNA; **d** DNA Unwinding Assay. Supercoiled (or relaxed) pBR322 plasmid DNA (lane 1, - Wheat germ TOPO I) was incubated in the absence (lane 2, + Wheat germ TOPO I) or presence of analysed compounds prior to incubation with wheat germ TOPO I. SC, supercoiled DNA; R, relaxed DNA; T, DNA topoisomers.

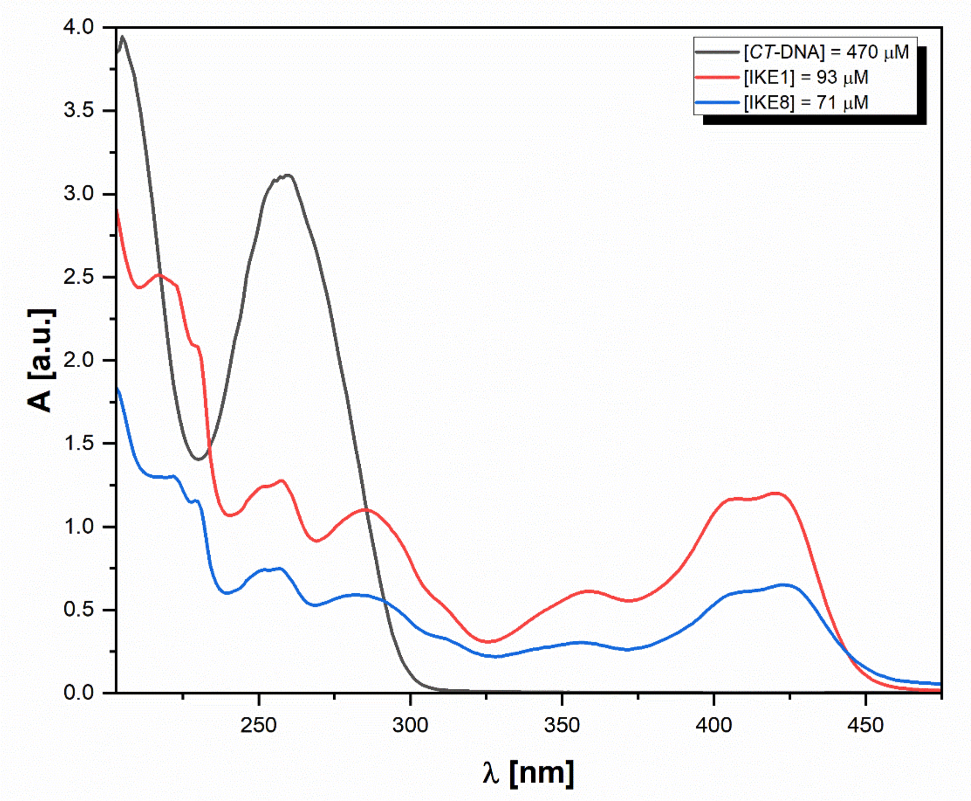

Figure S5. Electronic spectra of IKE1, IKE8, and *CT*-DNA solutions were recorded to establish the preconditions of interaction investigations.

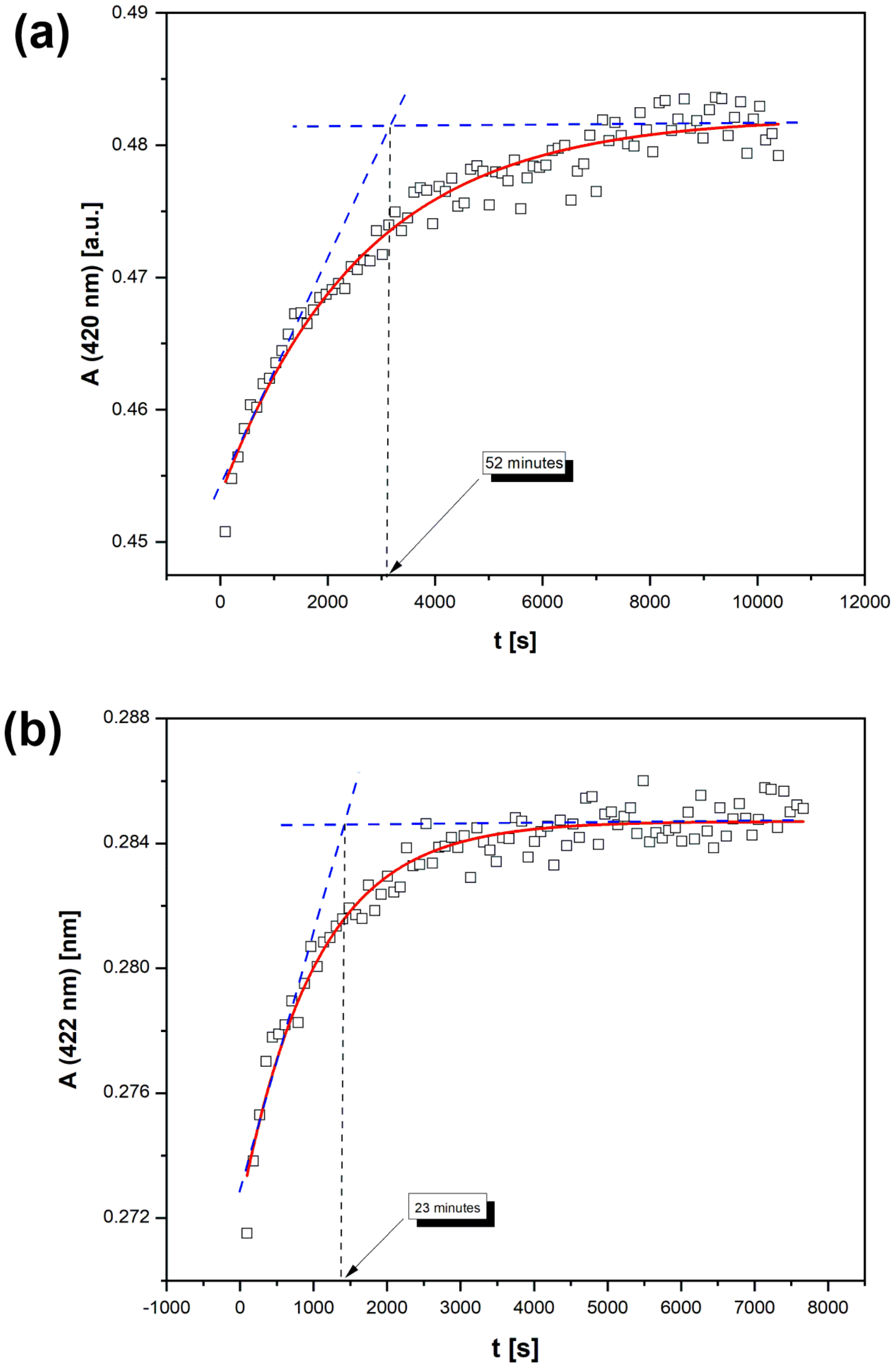

Figure S6. The relationship spectrophotometrically established for the step's time detection of *CT*-DNA 10 μL dose interaction for both: IKE1 (a) and IKE8 (b), respectively.

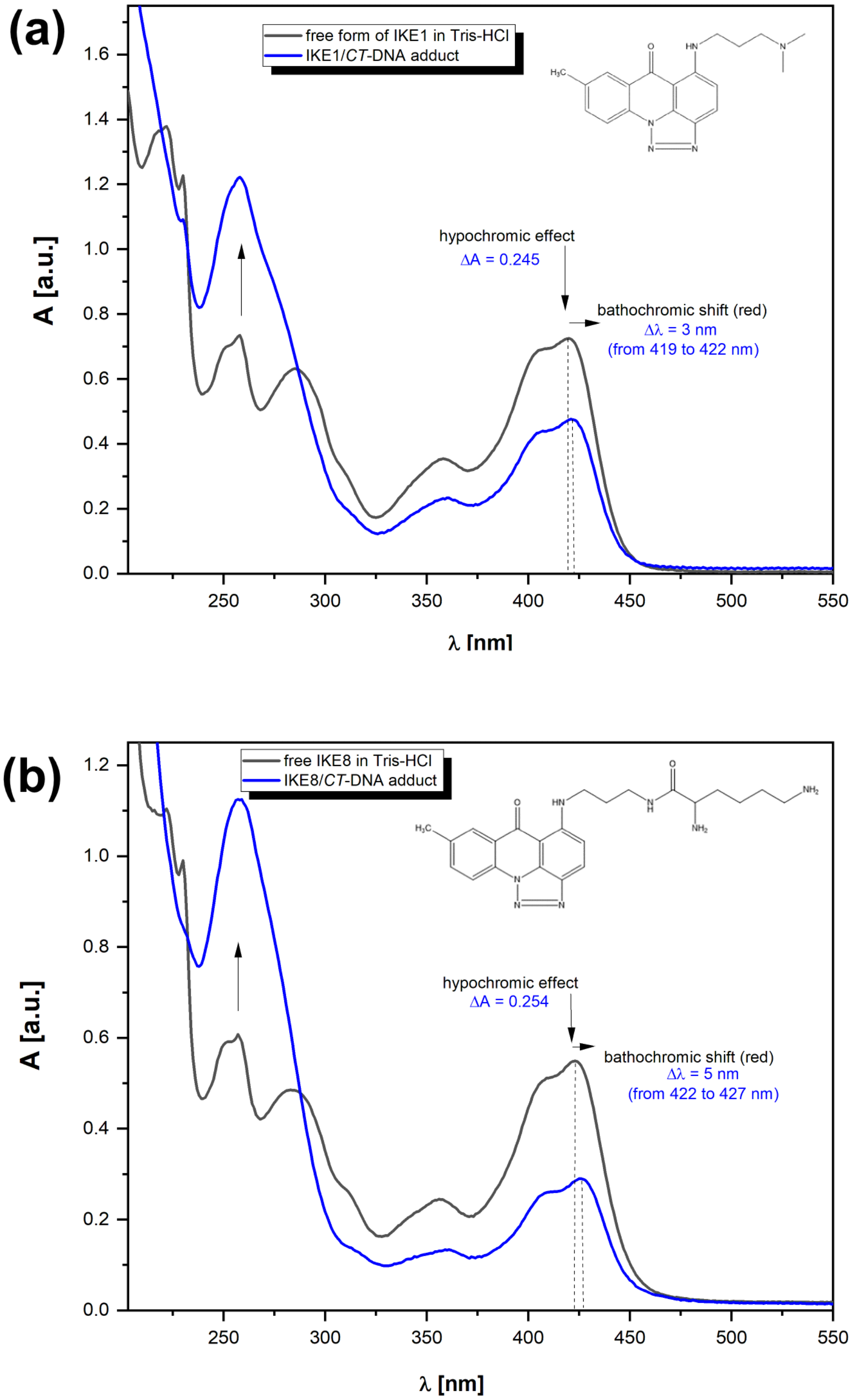

Figure S7. UV-Vis spectra of Tris-HCl buffer solutions obtained for IKE1 free state (black) and IKE1/CT-DNA adduct formed (blue) (a); IKE8 free form (black) and IKE8/*CT*-DNA adduct formed (blue) (b) together with parameters for spectroscopic effects observed: intensity changes and/or chromic shifts.

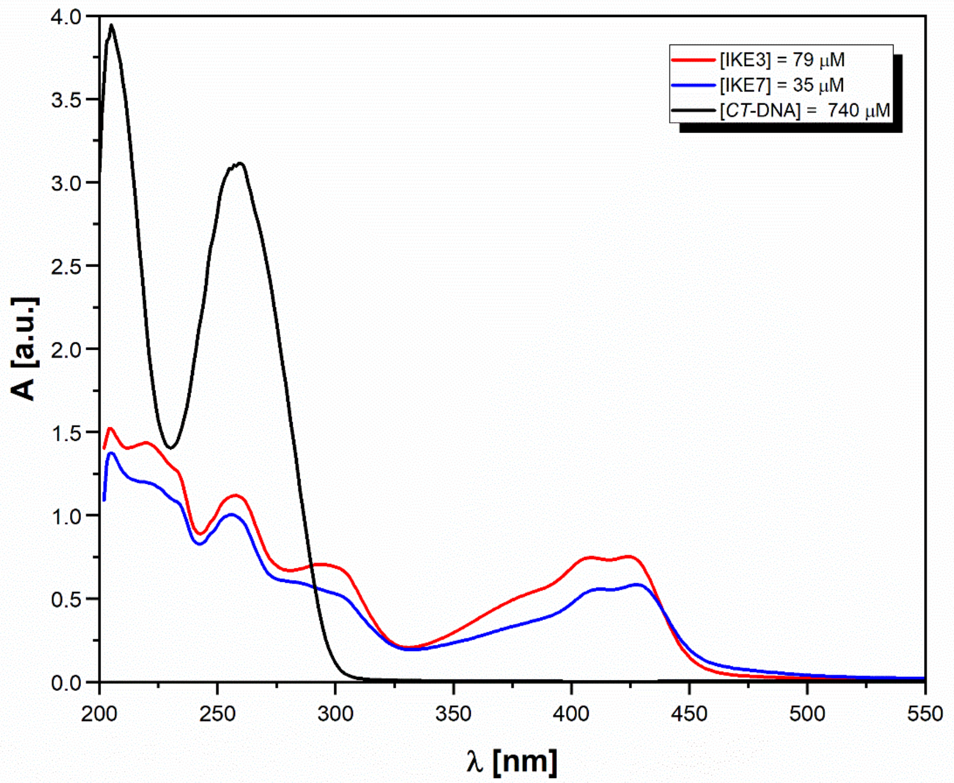

Figure S8. Electronic spectra of IKE3, IKE7, and *CT*-DNA solutions were recorded to establish the preconditions of interaction investigations.

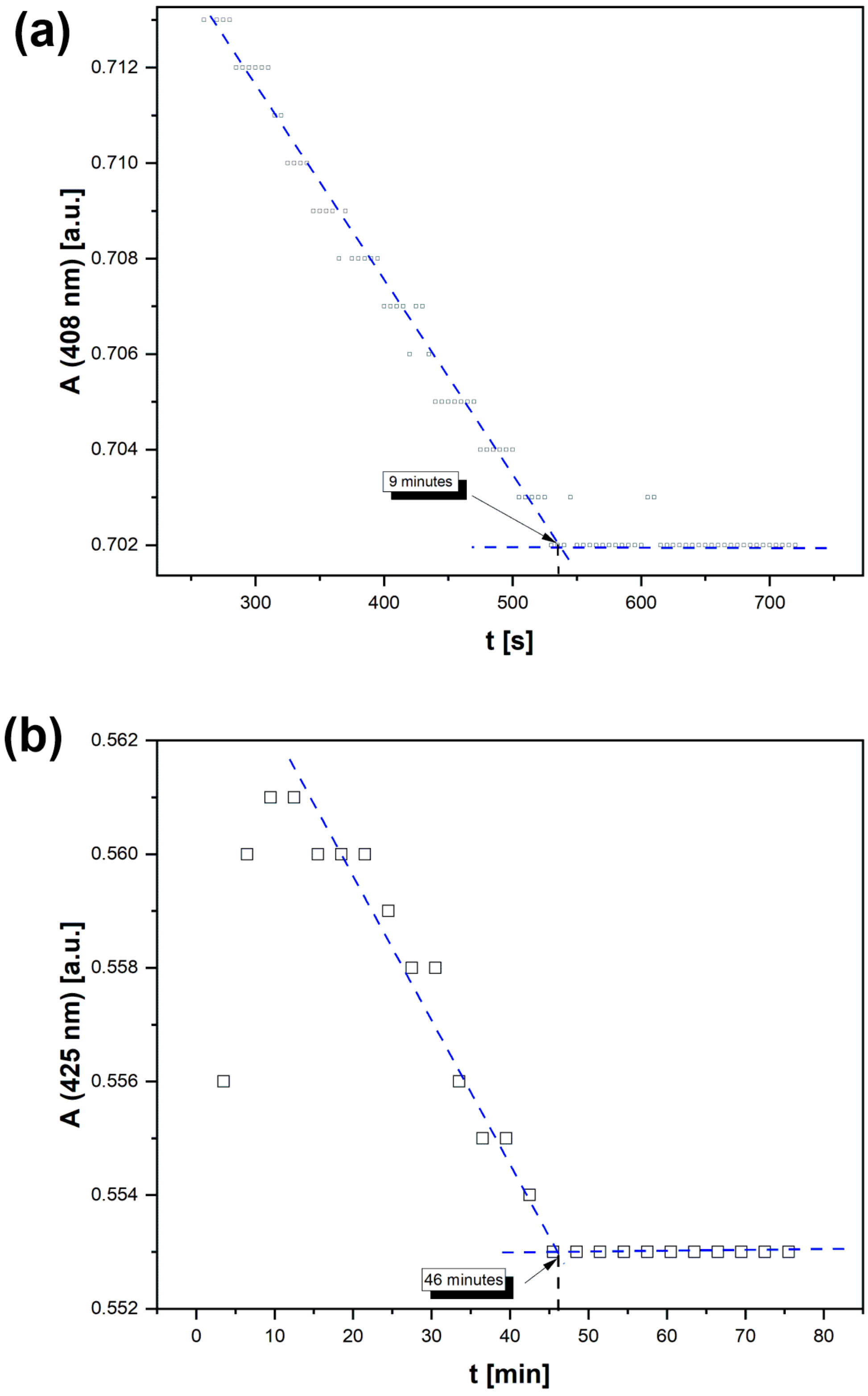

Figure S9. The relationship spectrophotometrically established for the step's time detection of *CT*-DNA: 20 μL and 50 μL dose interaction with IKE3 (a) and IKE7 (b), respectively.

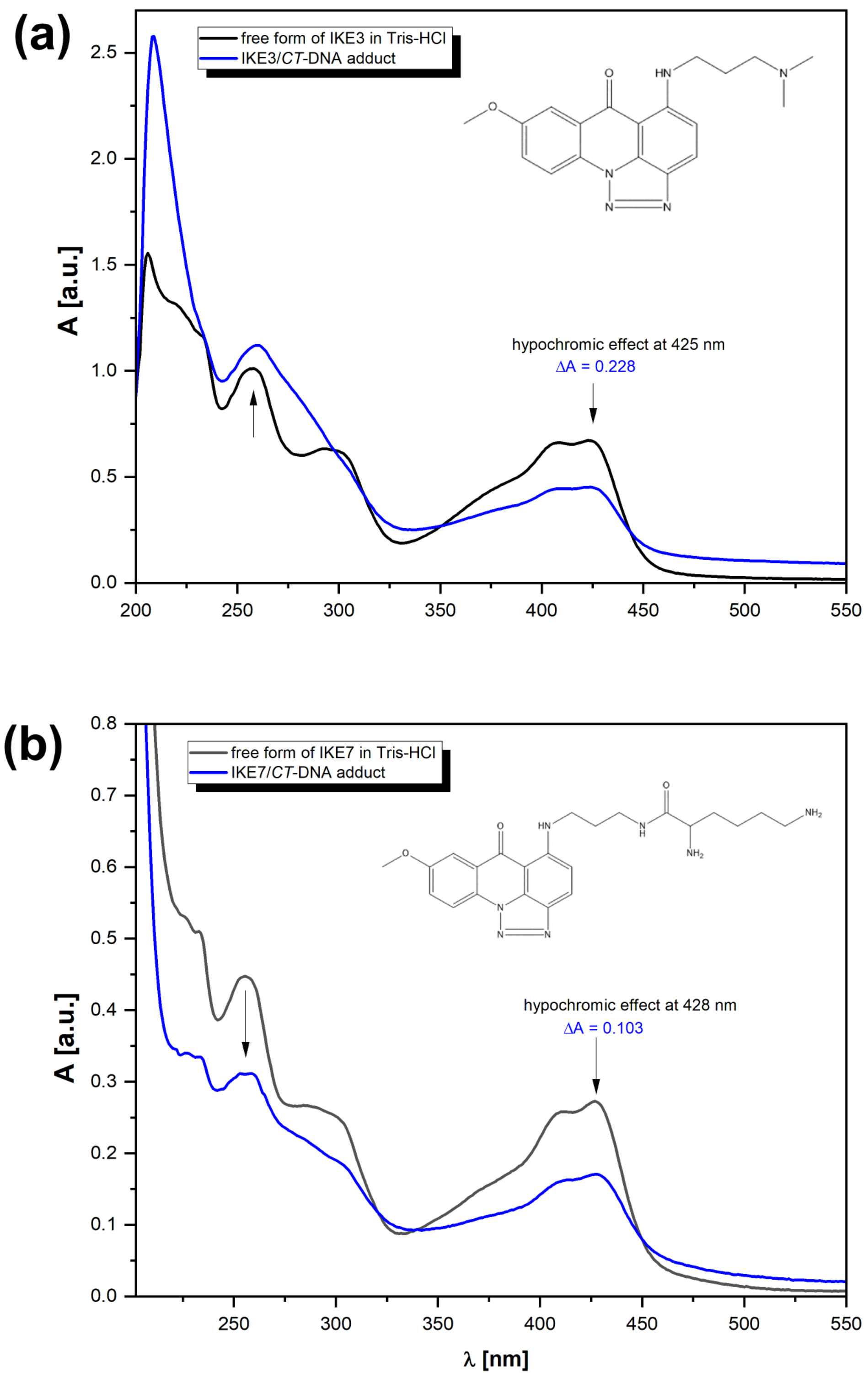

Figure S10. UV-Vis spectra of Tris-HCl buffer solutions obtained for IKE3 free state (black) and IKE3/CT-DNA adduct formed (blue) (a); IKE7 free form (black) and IKE7/CT-DNA adduct formed (blue) (b) together with parameters for spectroscopic effects observed: intensity changes and/or chromic shifts.

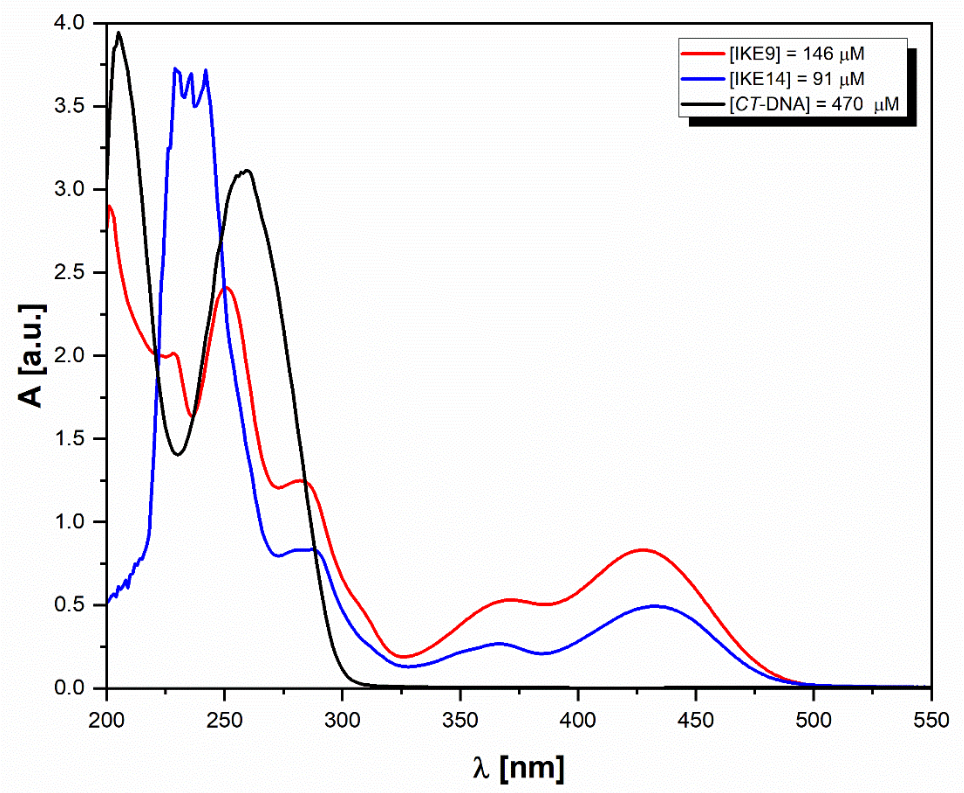

Figure S11. Electronic spectra of IKE9, IKE14, and CT-DNA solutions were recorded to establish the preconditions of interaction investigations.

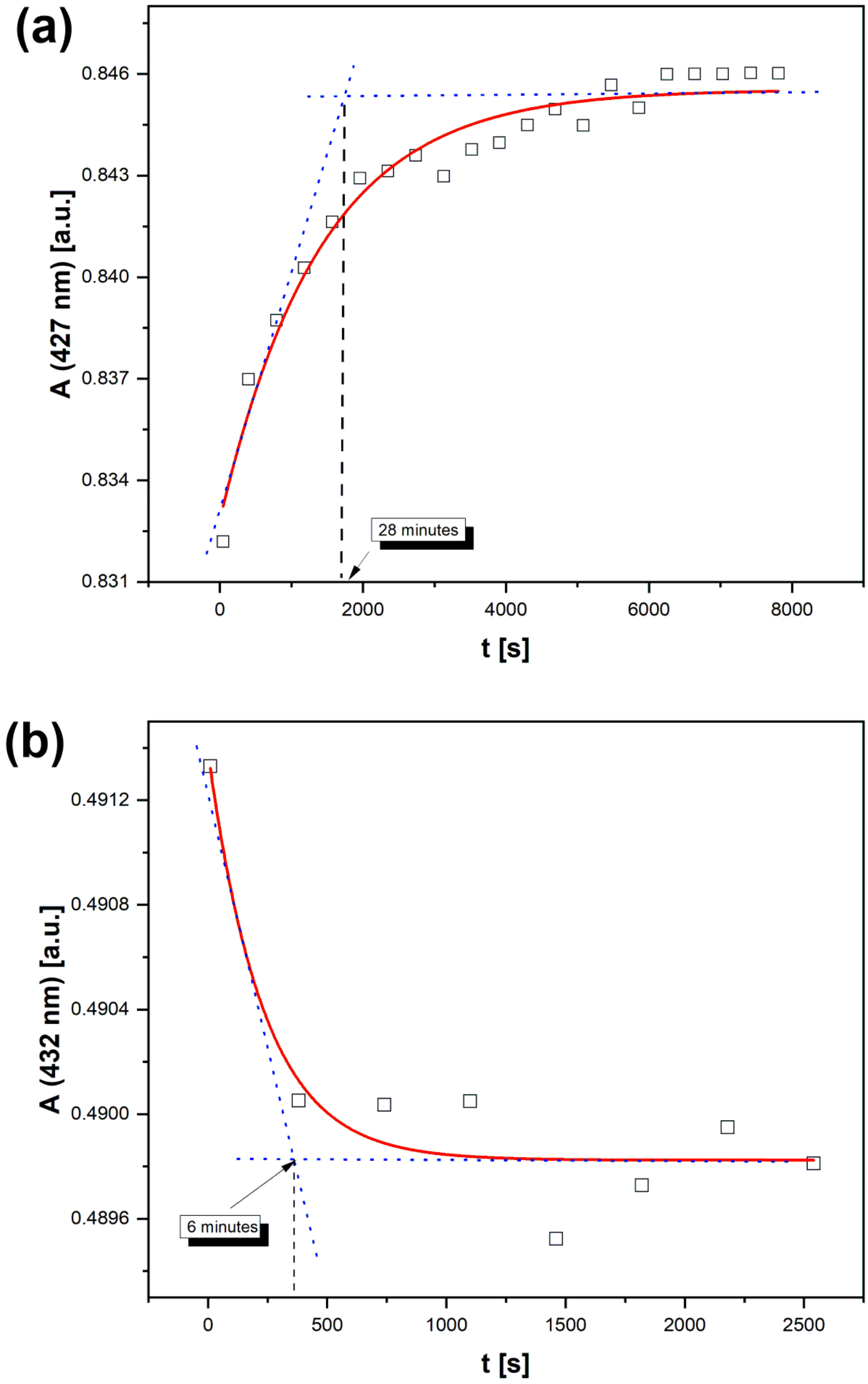

Figure S12. The relationship spectrophotometrically established for the step's time detection of CT-DNA 20 μL dose interaction with IKE9 (a) and IKE14 (b), respectively.

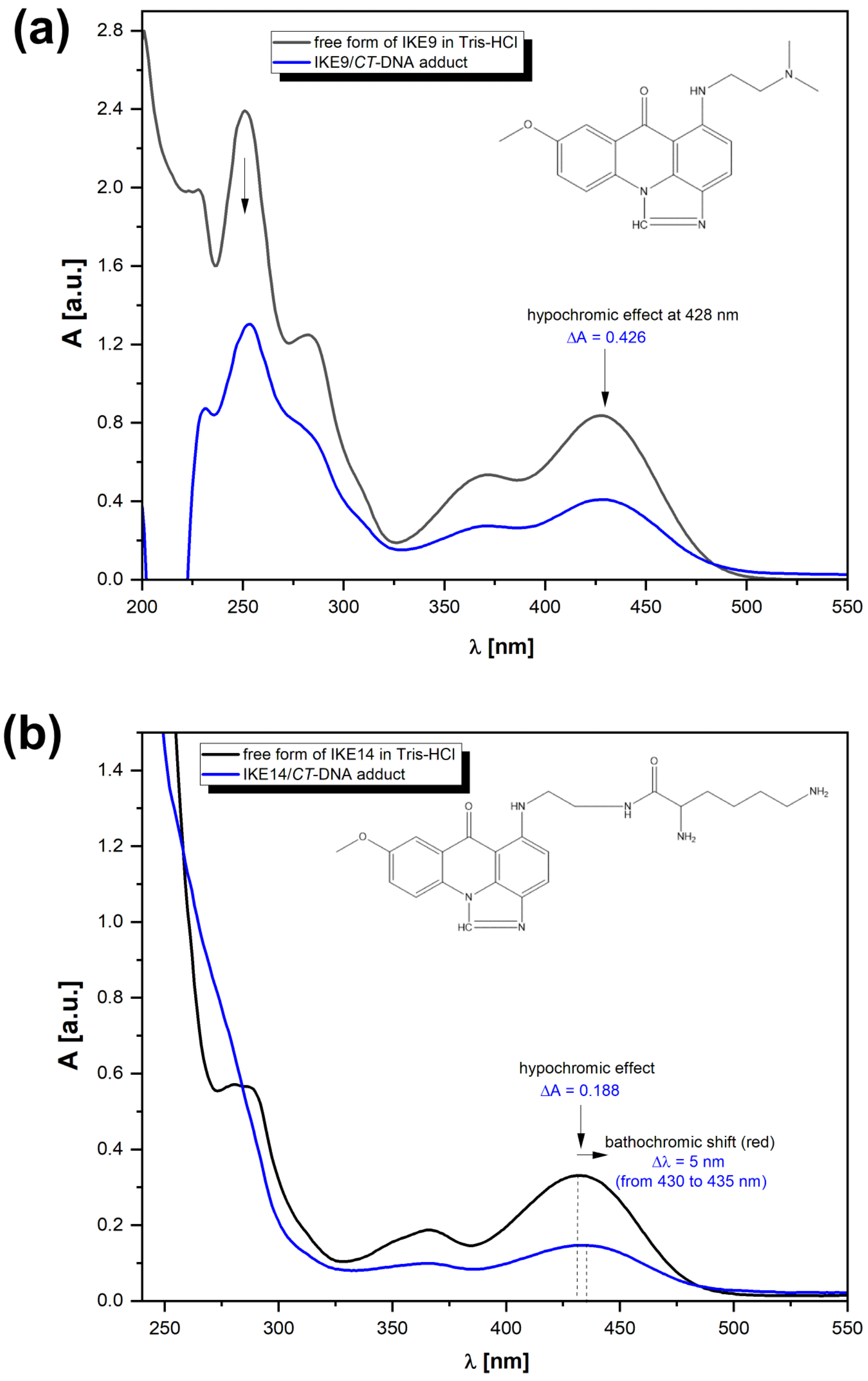

Figure S13. UV-Vis spectra of Tris-HCl buffer solutions obtained for IKE9 free state (black) and IKE9/CT-DNA adduct formed (blue) (a); IKE14 free form (black) and IKE14/*CT*-DNA adduct formed (blue) (b) together with parameters for spectroscopic effects observed: intensity changes and/or chromic shifts.

Table S1 . Values of the observed changes in the absorption bands' positions between the free form of acridness (IKEs) and their adducts formed as a result of interaction with DNA.

**Compound acronyms**	**Hypochromic effects (ΔA)**	**Bathochromic shifts (Δλ [nm])**
**IKE1**	0.245 (λ = 419 nm)	3 (419 nm → 422 nm)
**IKE8**	0.254 (λ = 422 nm)	5 (422 nm → 427 nm)
**IKE3**	0.228 (λ = 425 nm)	ND*
**IKE7**	0.103 (λ = 428 nm)	ND*
**IKE9**	0.426 (λ = 428 nm)	ND*
**IKE14**	0.188 (λ = 430 nm)	5 (430 nm → 435 nm)

*ND = not detected

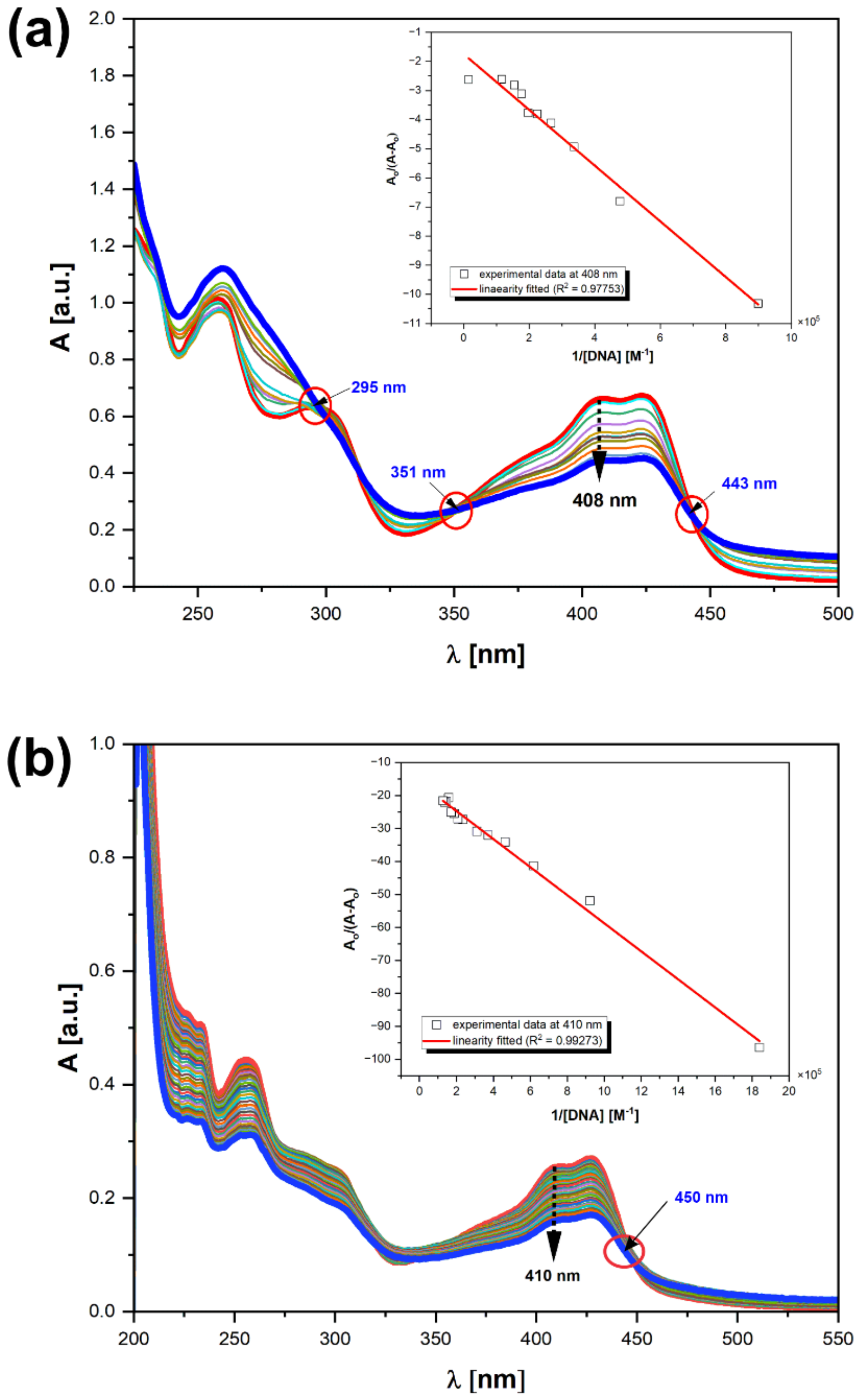

Figure S14. Absorption spectra of IKE3 (a) and IKE7 (b) in the presence of increasing amounts of *CT*-DNA. Arrows indicate that absorbance changes upon increasing *CT*-DNA concentrations. Inset: plot of Ao/(A-Ao) = f(1/[DNA]) established as a result of both compounds' interactions through titration with *CT*-DNA in Tris-HCl buffer (5 mM/50 mM NaCl; pH 7.43).

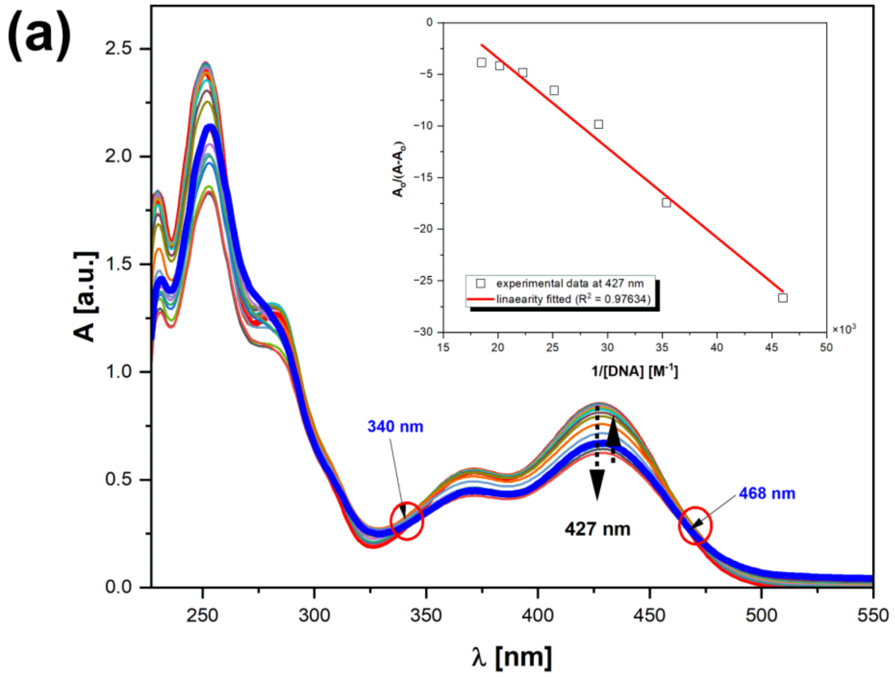

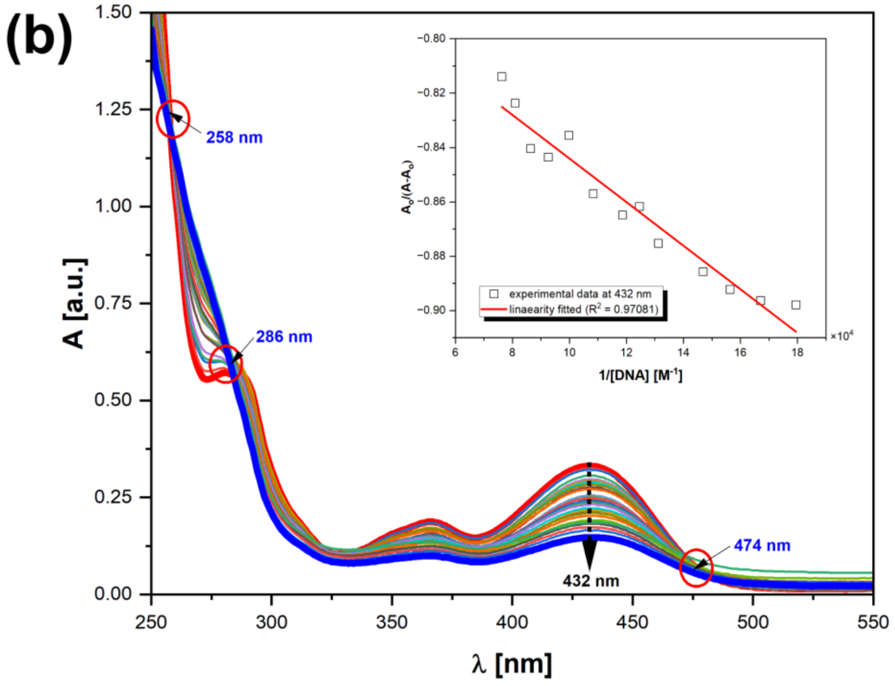

Figure S15. Absorption spectra of IKE9 (a) and IKE14 (b) in the presence of increasing amounts of *CT*-DNA. Arrows indicate that absorbance changes upon increasing *CT*-DNA concentrations. Inset: plot of Ao/(A-Ao) = f(1/[DNA]) established as a result of both compounds' interactions through titration with *CT*-DNA in Tris-HCl buffer (5 mM/50 mM NaCl; pH 7.43).

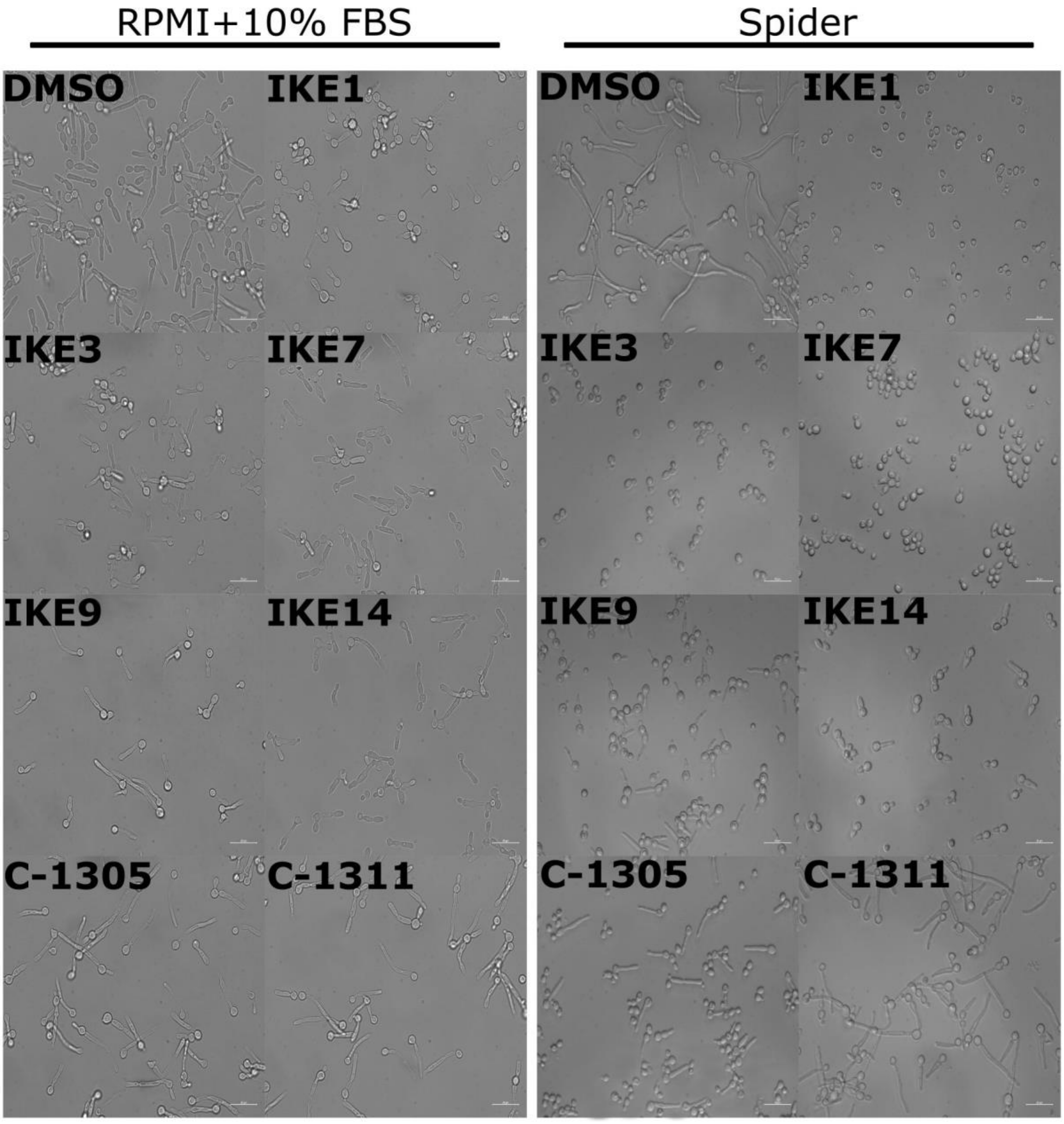

Figure S16. Microscopy analysis of changes in the morphology of *C. albicans* ATCC 10231 cells following treatment with compounds. The cells were subjected to light microscopy, and the scale bar represents 20 μm. The analysis was conducted after a 3-hour incubation period in either RPMI + 10% FBS or Spider medium.

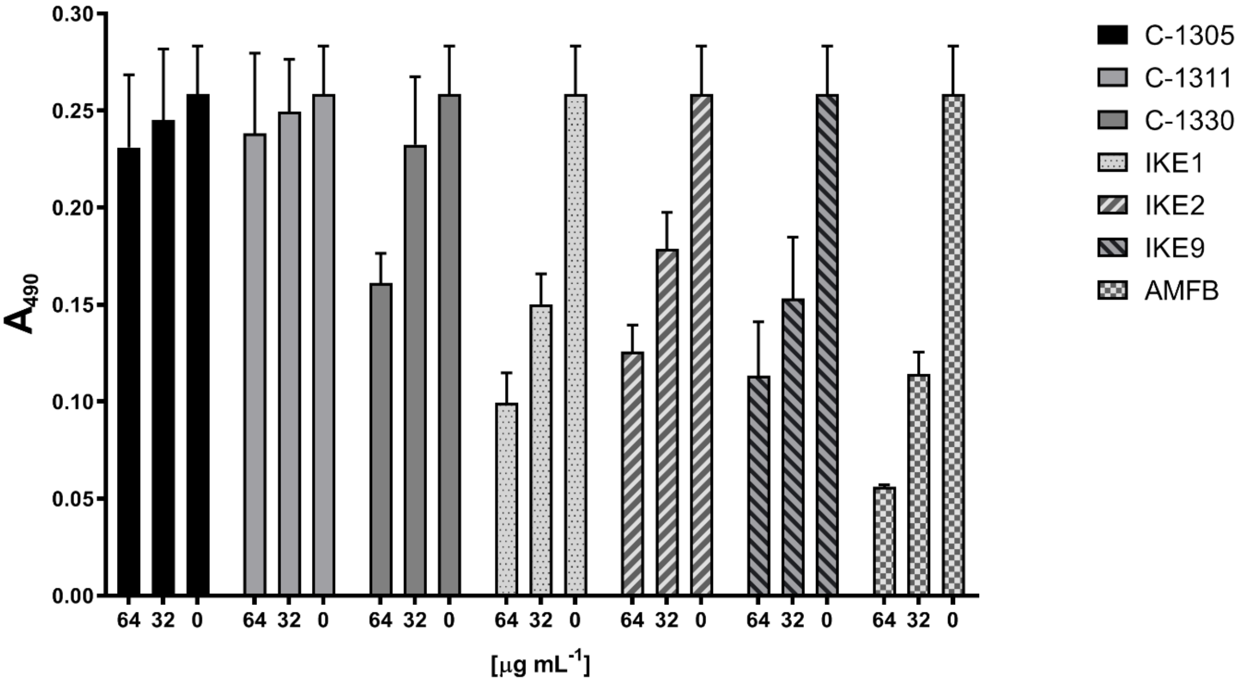

Figure S17. Antibiofilm activity of C-1305 and C-1311 and their novel derivatives. The metabolic activity of *C. albicans* ATCC 10231 biofilms was performed by XTT colorimetric method as described in the Material and Methods section. The experiments were performed at least in five replicates (A_{490nm} ± SEM); AMFB Amphotericin B.
